# Risk of spontaneous preterm birth and fetal growth associates with fetal *SLIT2*

**DOI:** 10.1371/journal.pgen.1008107

**Published:** 2019-06-13

**Authors:** Heli Tiensuu, Antti M. Haapalainen, Minna K. Karjalainen, Anu Pasanen, Johanna M. Huusko, Riitta Marttila, Marja Ojaniemi, Louis J. Muglia, Mikko Hallman, Mika Rämet

**Affiliations:** 1 PEDEGO Research Unit, Medical Research Center Oulu, University of Oulu, and Department of Children and Adolescents, Oulu University Hospital, Oulu, Finland; 2 Division of Human Genetics, Center for Prevention of Preterm Birth, Perinatal Institute, Cincinnati Children's Hospital Medical Center, Department of Pediatrics, University of Cincinnati College of Medicine, March of Dimes Prematurity Research Center Ohio Collaborative, Cincinnati, Ohio, United States of America; 3 Faculty of Medicine and Health Technology, Tampere University, Tampere, Finland; Stanford University School of Medicine, UNITED STATES

## Abstract

Spontaneous preterm birth (SPTB) is the leading cause of neonatal death and morbidity worldwide. Both maternal and fetal genetic factors likely contribute to SPTB. We performed a genome-wide association study (GWAS) on a population of Finnish origin that included 247 infants with SPTB (gestational age [GA] < 36 weeks) and 419 term controls (GA 38–41 weeks). The strongest signal came within the gene encoding slit guidance ligand 2 (*SLIT2*; rs116461311, minor allele frequency 0.05, *p* = 1.6×10^−6^). Pathway analysis revealed the top-ranking pathway was axon guidance, which includes *SLIT2*. In 172 very preterm-born infants (GA <32 weeks), rs116461311 was clearly overrepresented (odds ratio 4.06, *p* = 1.55×10^−7^). *SLIT2* variants were associated with SPTB in another European population that comprised 260 very preterm infants and 9,630 controls. To gain functional insight, we used immunohistochemistry to visualize SLIT2 and its receptor ROBO1 in placentas from spontaneous preterm and term births. Both SLIT2 and ROBO1 were located in villous and decidual trophoblasts of embryonic origin. Based on qRT-PCR, the mRNA levels of *SLIT2* and *ROBO1* were higher in the basal plate of SPTB placentas compared to those from term or elective preterm deliveries. In addition, in spontaneous term and preterm births, placental *SLIT2* expression was correlated with variations in fetal growth. Knockdown of *ROBO1* in trophoblast-derived HTR8/SVneo cells by siRNA indicated that it regulate expression of several pregnancy-specific beta-1-glycoprotein (*PSG*) genes and genes involved in inflammation. Our results show that the fetal *SLIT2* variant and both *SLIT2* and *ROBO1* expression in placenta and trophoblast cells may be correlated with susceptibility to SPTB. SLIT2-ROBO1 signaling was linked with regulation of genes involved in inflammation, *PSG* genes, decidualization and fetal growth. We propose that this receptor-ligand couple is a component of the signaling network that promotes SPTB.

## Author summary

Worldwide, more than 10% of babies are born prematurely without effective means of prevention. Premature birth is associated with mortality and lifelong comorbidities. Aggregation of spontaneous preterm birth in certain families suggests that there are underlying genetic factors that predispose to preterm birth. Both maternal and fetal genomes likely affect susceptibility. We set out to identify fetal genetic variants that predispose infants to premature birth in a population of Finnish origin. Our results from a genome-wide association study indicate that a variant of slit guidance ligand 2 (*SLIT2*) is associated with the risk of spontaneous preterm birth. Furthermore, *SLIT2* and its receptor roundabout guidance receptor 1 *(ROBO1)* are expressed in placental cells, and their mRNA levels are higher in placentas from spontaneous preterm deliveries compared to term controls. Based on gene knockdown experiments in cultured placental tissue–derived cells, ROBO1 regulates expression of pregnancy-specific beta-1-glycoprotein (*PSG*) genes and genes involved in inflammation. Thus, our results indicate that the fetal *SLIT2* variant and expression of both *SLIT2* and *ROBO1* in placental cells are correlated with susceptibility to spontaneous preterm birth. We propose that this receptor–ligand pair is a component of the signaling network that promotes spontaneous preterm birth.

## Introduction

Preterm live births that take place before 37 completed weeks of gestation and even as early as 22–24 weeks are a global problem. Up to 11.1% (15 million babies) of all births worldwide occur prematurely, and approximately 45–50% of them are idiopathic or spontaneous [1–3]. Complications caused by preterm birth are the most common cause of neonatal deaths and the largest direct cause of deaths of children <5 years of age [1,3]. The research focusing on spontaneous preterm birth (SPTB) has been complicated by etiological, pathophysiological, and genetic heterogeneities. Multiple events are associated with SPTB, either independently or in concert [4]. These include intrauterine inflammation, called chorioamnionitis, preterm premature rupture of fetal membranes (PPROM) and abnormal fetal growth relative to uterine size [5,6]. It is important to find new biomarkers for early detection of SPTB. Currently, our understanding of the early molecular pathways leading to SPTB is incomplete and there are no effective means to prevent SPTB. Knowledge of how maternal and fetal genomes contribute to the risk of SPTB could provide more personalized tools to prevent it [7].

Epidemiological studies have shown that both fetal and maternal genes affect fetal growth, birth weight, birth length, head circumference, and gestational age (GA) [8–11]. A recent study indicated that variants of the fetal and maternal genome independently affect normal variations in birth weight [12]. In addition, maternal and fetal genomes are also considered to affect the susceptibility to preterm birth and duration of pregnancy in general [13–15]. The intrauterine environment influences fetal growth, and adverse intrauterine events affect pregnancy length not only in elective preterm pregnancies but also in SPTB [11]. Genetic analysis of 244,000 Swedish births resulting in twins, full siblings, and half-siblings revealed that 13% and 21% of the variation in birth timing is explained by fetal and maternal genetic factors, respectively [9]. Overall, preterm birth is a phenotype with contributions from both, maternal and fetal genomes that may have separate contributions and together with environmental factors, interactively determine the outcome [7,13,16].

A recent study that included a population of > 40,000 women and replication cohorts of > 8000 women identified several common variants in *EBF1*, *EEFSEC*, and *AGTR2* that showed associations with preterm birth at a genome-wide significance level [13]. In addition, other genome-wide association studies (GWAS) and SPTB genetic studies focused on mother [17–19] or infant [19–21] genomic signals have discovered genetic loci associated with preterm birth and gestational length. Studies that focused on fetal genomes have not revealed replicable associations between fetal genetic factors and SPTB.

Many pathways and cellular processes are reported to be associated with SPTB, including response to infection, regulation of inflammation, stress, and other immunologically mediated processes [3]. According to our current understanding, inflammatory pathways also have roles in the initiation of spontaneous term birth, as normal labor starts when there is a shift in signaling between anti-inflammatory and proinflammatory pathways in the myometrium. This shift appears to involve many chemokines such as interleukin 8 (IL8), cytokines such as IL1 and IL6, and contraction-associated proteins such as oxytocin receptor (OXTR), connexin 43 (CX43), and prostaglandin receptors [22]. Therefore, it is likely that changes in inflammation-associated pathways also contribute to preterm birth. Evidence from candidate gene studies supports the role of inflammation-related factors in SPTB. For example, polymorphisms of the genes encoding TLR4, TNF, IL1B, interferon gamma (IFNγ), IL6, and matrix metalloproteinases may be associated with increased risk of SPTB [5].

The aim of the present study was to use a GWAS to investigate fetal genetic variants that may predispose infants to SPTB in a homogeneous population of Finnish origin. A variant of slit guidance ligand 2 (*SLIT2*) had the most suggestive association with SPTB in the GWAS and in a genetic pathway analysis. Therefore, we characterized SPTB-associated expression of *SLIT2* and its receptor *ROBO1* in the placenta and subsequently conducted experiments with relevant placenta-associated cells.

## Results

### GWAS association signals for spontaneous preterm birth

**Fig 1:**
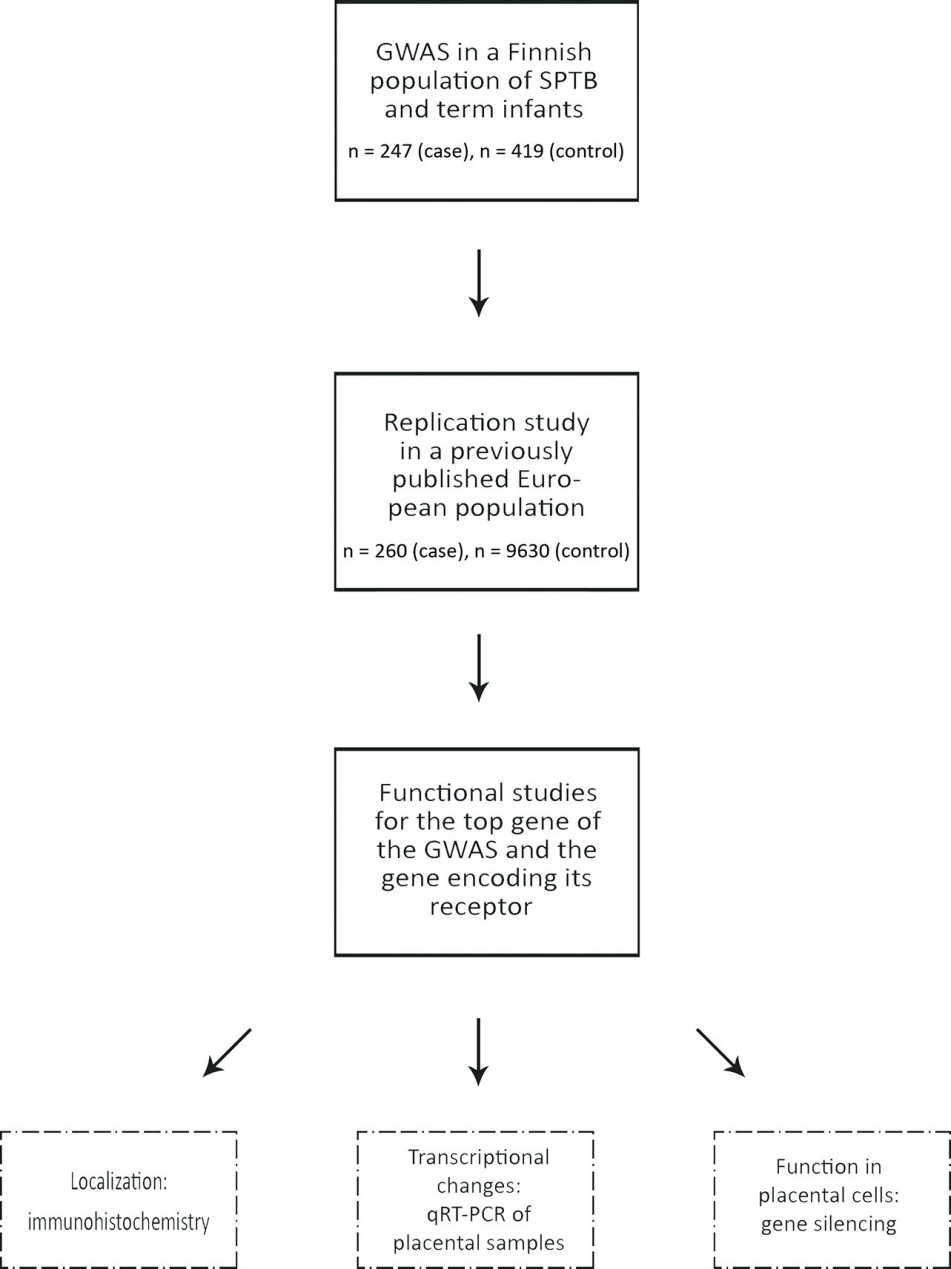
Overview of the study workflow.

In order to find fetal genetic factors associated with predisposition to SPTB, we analyzed polymorphisms encompassing the entire genome for associations with SPTB. After quality control, 247 infants born spontaneously preterm and 419 infants born at term remained for inclusion in the GWAS. We performed the analysis for both GA (quantitative trait) and SPTB (dichotomous setting). However, due to sample collection bias resulting in skewed GA distribution, we acknowledge that the results of the quantitative trait analysis should be interpreted with caution. Fig 1 summarizes the study workflow.

**Fig 2:**
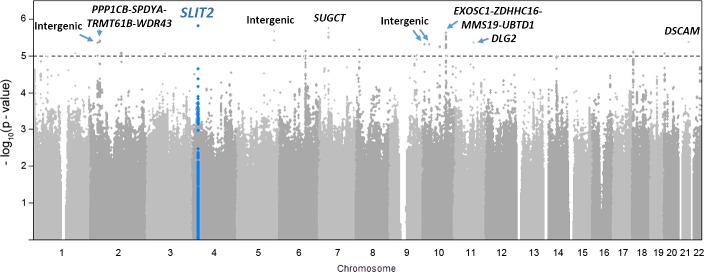
Manhattan plot of GWAS of SPTB. Each dot represents −log_10_(*p*) value of a single SNP in association analysis. Dashed line denotes level of suggestive significance (−log_10_(*p*) > 5). *SLIT2*, *SUGCT*, and *EXOSC1-ZDHHC16-MMS19-UBTD1* were among the best associating loci.

We detected several suggestive associations (*p* < 10^−5^) in the GWAS (Fig 2, Table 1). The two most promising regions were within the genes encoding *SLIT2* (rs116461311, *p* = 1.6 × 10^−6^) and succinyl-CoA:glutarate-CoA transferase (*SUGCT*; rs57670997, *p* = 1.8 × 10^−6^). We also detected suggestive associations for GA as a quantitative trait (S2 Table, S1 Fig). SNP rs116461311 within the *SLIT2* gene showed the most significant signal for GA (*p* = 3.1 × 10^−7^, S1 Fig). In addition to *SLIT2*, four regions showed suggestive signals both in the primary setting and in the GWAS of GA; these signals were within *SUGCT*, an intergenic region in chromosome 6 (nearest loci *LOC105377949* and *LOC107986634*), and within the genes encoding anaplastic lymphoma receptor tyrosine kinase (*ALK*) and DLC1 Rho GTPase activating protein (*DLC1*).

**Table 1:** Single-nucleotide polymorphisms suggestively associated with spontaneous preterm birth.

Chr	Locus^a^	SNP^b^	Reference allele	Allele frequency	Odds ratio (95% confidence interval)	*p*
4	*SLIT2*	rs116461311	C	0.05	3.43 (2.01–5.86)	1.58E-6
7	*SUGCT*	rs57670997	Del	0.68	0.56 (0.44–0.71)	1.79E-6
9	Intergenic (*LOC105376188*, *LOC105376190*)	rs35464758	Del	0.05	2.82 (1.67–4.80)	1.82E-6
5	Intergenic (*LOC100128898*, *LOC100130177*)	rs4704916	A	0.19	1.91 (1.45–2.52)	2.22E-6
10	*EXOSC1-ZDHHC16-MMS19-UBTD1*^c^	rs10678727	Ins	0.58	1.77 (1.40–2.23)	2.39E-6
2	Intergenic (*THUMPD2*, *SLC8A1*)	rs17480505	C	0.35	1.72 (1.36–2.17)	3.88E-6
21	*DSCAM*	rs9974083	A	0.24	1.73 (1.34–2.24)	4.32E-6
2	*PPP1CB-SPDYA-TRMT61B-WDR43*^c^	rs200334508	G	0.87	0.51 (0.37–0.71)	4.44E-6
11	*DLG2*	rs202170665	Ins	0.08	0.40 (0.25–0.65)	4.59E-6
10	Intergenic *(RBM17*, *LOC1019280803)*	rs10905856	T	0.08	2.28 (1.53–3.41)	4.87E-6
10	*ABI1*	rs72385215	Del	0.33	0.56 (0.44–0.72)	4.89E-6
10	*ADAMTS14*	rs12765664	A	0.93	0.40 (0.26–0.61)	5.95E-6
8	*DLC1*	rs7006225	A	0.77	0.56 (0.43–0.72)	6.99E-6
6	Intergenic (*LOC105377949*, *LOC107986634*)	rs1418269	C	0.69	1.73 (1.34–2.22)	7.51E-6
18	*DLGAP1*	rs610269	G	0.54	0.61 (0.49–0.76)	7.97E-6
2	*NCKAP5*	rs1966628	T	0.45	1.64 (1.31–2.05)	8.29E-6
20	*HSPA12B*	rs58505239	T	0.20	1.75 (1.34–2.29)	8.66E-6
3	Intergenic (*LOC105377173*, *ROBO1*)	rs115723230	T	0.03	3.33 (1.70–6.54)	8.70E-6
1	*EIF1P3*	rs539974331	Del	0.04	3.57 (1.95–6.55)	8.92E-6
9	*SVEP1*	rs199638820	Del	0.56	1.61 (1.29–2.02)	9.56E-6
2	*ALK*	rs7608573	T	0.20	0.52 (0.38–0.70)	9.99E-6

^a^Respective gene shown for SNPs within genes; two nearest genes shown in parentheses for intergenic SNPs.

^b^SNP with the most significant signal shown for each locus.

^c^Association signal spans the indicated genes.

We further analyzed very preterm and moderate-to-late preterm SPTB infants separately against term-born controls (S3 and S4 Tables). Three regions showed suggestive signals (*p* < 10^−5^) both in the primary setting and in the analysis of very preterm birth (S3 Table). These signals were within *SLIT2*, in an intergenic region on chromosome 2 (nearest genes *THUMPD2* and *SLC8A1*), and within the region encompassing the *EXOSC1*, *ZDHHC16*, *MMS19*, and *UBTD1* genes, which encode exosome component 1; zinc finger DHHC-type containing 16; MS19 homolog, cytosolic iron-sulfur assembly component; and ubiquitin domain containing 1, respectively. The minor allele of *SLIT2*, SNP rs116461311, was overrepresented (OR 4.06, *p* = 1.55 × 10^−7^) in very preterm-born infants (GA < 32 weeks) compared to term-born infants. In moderate-to-late preterm infants (GA 32–36 weeks), two regions showed suggestive signals (S4 Table) that were also evident in the primary analysis: an intergenic region on chromosome 3 (nearest loci *LOC105377173* and *ROBO1*) and within *ADAMTS14*, which encodes ADAM metallopeptidase with thrombospondin type 1 motif 14.

We also studied associations with SPTB within the contexts of PPROM and no PPROM. There were separate suggestive associations with SPTB-PPROM and with SPTB without PPROM (S5 and S6 Tables). For the SLIT2 region, the effects were similar for infants born after PPROM (rs116461311, OR = 3.5, *p* = 3.0 × 10^−5^) and for those born after spontaneous onset of labor with intact fetal membranes (rs116461311, OR = 3.6, *p* = 1.3 × 10^−5^).

To investigate potential maternal transmission of the minor allele of SPTB-associated rs116461311 (in *SLIT2*), we checked the MAF of the variant in maternal samples. The frequency of C-rs116461311 was 0.059 in SPTB mothers (*n* = 230) and 0.035 in mothers with term delivery (*n* = 378). Transmission analysis was not feasible because of the low minor allele counts.

### Replication of *SLIT2* association in a European population

SNPs with suggestive association signals in the region of the top GWAS gene (*SLIT2*) were examined for association with SPTB in a European population (*n* = 9890) [20]. In a European replication population of very preterm and term-born controls, rs12503652 and rs79034379, which correlate with the best GWAS SNP of the *SLIT2* region (rs116461311), were associated with SPTB (Table 2). The two SNPs were in high LD with one another (*r*^2^ = 0.85, D′ = 0.95) in the Finnish individuals in 1000 Genomes phase3 data.

**Table 2:** Two SNPs that correlated with the top GWAS SNP rs116461311 in *SLIT2* showed association with SPTB in European replication data.

	R^2^ in 1000 Genomes FIN		Discovery GWAS		European replicate	
SNP	rs116461311	Reference allele	Odds ratio	*P*	Odds ratio	*p*
**rs116461311**	**1**	C	3.43	1.58E-6	*NA*	*NA*
**rs12503652**	0.58	G	2.11	3.83E-4	1.47	0.023
**rs79034379**	0.61	G	2.02	3.49E-4	1.46	0.032

### Functional annotation of the most promising genetic variants

To investigate if the SNPs within regions with the most promising signals in the GWAS (*p* < 10^−5^) have functional consequences, we screened the GTEx data to examine whether any of the suggestively associated SNPs colocalize with *cis* eQTLs; that is, whether they correlate with mRNA levels in the analyzed tissues. In the GTEx data, 26 of the SNPs with *p* < 10^−5^ for association with SPTB overlapped with significant eQTLs (Table 3). These SNPs were located within five different regions; the majority were in the region encompassing *EXOSC1*, *ZDHHC16*, *MMS19*, and *UBTD1* (Tables 1, S3 and S5). These SNPs correlated with mRNA levels of the following genes in different tissues: *ZDHHC16*, *MMS19*, *FRAT1*, *ANKRD2*, *UBTD1*, and *RRP12*. Associated SNPs within the top GWAS region, *SLIT2*, were not associated with mRNA levels. According to the GWAS catalog, none of the suggestively associated SNPs had been significantly associated with any phenotype.

**Table 3:** Suggestively associated SNPs that overlap with *cis* eQTLs.

SNP information			*cis*-eQTL information		
GWAS region	SNP^a^	*p* in GWAS	eGene	eQTL *p*^b^	Tissue
*EXOSC1-ZDHHC16-MMS19-UBTD1*	rs7897727^c^	2.03E-06	*ZDHHC16* *MMS19* *FRAT1*	2.0E-18 1.7E-07 2.0E-05 2.6E-06 5.0E-06	Testis Adipose–Subcutaneous Breast–Mammary tissue Lung Cells–Transformed fibroblasts
*DSCAM*	rs9974083	4.32E-06	*PCP4*	2.3E-07	Artery—Tibial
Intergenic, chr6	rs1418269	7.51E-06	*RP1-142L7*.*5*	3.4E-05	Esophagus—Muscularis
*NCKAP5*	rs1966628^d^	8.29E-06	*NCKAP5*	1.3E-05	Testis
*HSPA12B*	rs58505239	8.66E-06	*CDC25B* *ADAM33*	1.8E-07 1.3E-06 2.9E-06	Adipose–Subcutaneous Artery—Tibial Esophagus—Muscularis

^a^Top eQTL SNP shown for each region.

^b^eQTL data from GTEx Portal V7.

^c^ Altogether, 15 suggestively associated SNPs within *EXOSC1-ZDHHC16-MMS19-UBTD1* region colocalized with eQTLs; these SNPs are eQTLs for *ZDHHC16*, *MMS19*, *FRAT1*, *ANKRD2*, *UBTD1*, and *RRP12*

^d^Altogether, eight suggestively associated SNPs within *NCKAP5* region are eQTLs for *NCKAP5*.

We further functionally annotated the SNPs (*p* < 10^−4^) within the *SLIT2* region with HaploReg, v 4.1. Some of the most promising SNPs within *SLIT2* (including the best associating variant rs116461311, as well as rs60126904, rs115707845, and rs16869667) were located within regions that contain histone marks and DNase-hypersensitive sites in several tissues. Furthermore, *SLIT2* SNPs (rs60126904 and rs115707845) mapped to predicted enhancers in several cell types and tissues, including neuronal cells, different cells in the brain, lung, spleen, adipose cells, colon and duodenum smooth muscle cells, fetal lung and kidney, and fetal membranes (amnion). Thus, there is some evidence of the putative regulatory effects of *SLIT2* SNPs in several tissues, including fetal tissues such as placenta.

### Biological pathways genetically associated with spontaneous preterm birth

To identify biological pathways associated with SPTB, we performed pathway analysis of the GWAS data to search for gene set enrichment. SPTB was associated with 16 Kyoto Encyclopedia of Genes and Genomes (KEGG) pathways [corrected (S7 Table) and FDR adjusted *p* < 0.05, Table 4]. The most significant pathways were axon guidance (*p* = 8.6 × 10^−10^), focal adhesion (*p* = 6.6 × 10^−7^), and vascular smooth muscle contraction (*p* = 1.4 × 10^−6^). Axon guidance, the most significant pathway, included *SLIT2* and the gene encoding its receptor, *ROBO1*. A Gene Ontology (GO) search revealed 35 GO terms associated with SPTB, with a false-discovery rate (FDR) of <0.05 (S8 Table). GO sets that included *SLIT2* and *ROBO1* are listed in S9 Table. The three most significant GO sets that included *SLIT2* were retinal ganglion cell axon guidance, telencephalon development, and negative chemotaxis. The three most significant GO sets that included *ROBO1* were telencephalon development, neuron recognition, and negative chemotaxis. These results led us to a more-detailed investigation of the roles of *SLIT2* and *ROBO1* in SPTB.

**Table 4:** Pathways associated with SPTB.

Pathway	Pathway name	*p*	Corrected *p*^a^	Top genes in pathway^b^
hsa04360	Axon guidance	8.6E-10	1.6E-07	***SLIT2***,*PAK6*,*NRP1*,*SEMA3E*,*ABLIM2*
hsa04510	Focal adhesion	6.6E-07	6.2E-05	*PPP1CB*,*AKT3*,*PAK6*,*EGFR*,*PARVB*
hsa04270	Vascular smooth muscle contraction	1.4E-06	8.9E-05	*PPP1CB*,*PLA2G4A*,*KCNMA1*,*ADRA1B*,*PRKG1*
hsa04720	Long-term potentiation	2.3E-05	1.1E-03	*PPP1CB*,*GRIN2B*,*GRM5*,*CAMK2G*,*PLCB1*
hsa04730	Long-term depression	7.0E-05	2.6E-03	*PLA2G4A*,*GRM5*,*PRKG1*,*LYN*,*PLCB1*
hsa04012	ErbB signaling pathway	4.1E-04	0.01	*AKT3*,*PAK6*,*EGFR*,*PLCG1*,*NRG2*
hsa04514	Cell adhesion molecules (CAMs)	4.4E-04	0.01	*HLA-DOA*,*NLGN1*,*CDH4*,*ITGAM*,*NCAM2*
hsa05412	Arrhythmogenic right ventricular cardiomyopathy (ARVC)	4.7E-04	0.01	*CACNA2D3*, *CACNB2*,*CACNA1D*,*ACTN1*

^a^KEGG pathways with corrected and FDR adjusted *p* < 0.05 shown.

^b^Top five genes or all genes with SNPs with *p* < 1E-02 in GWAS shown.

### SLIT2 and its receptor ROBO1 are localized in villous and decidual trophoblasts

**Fig 3:**
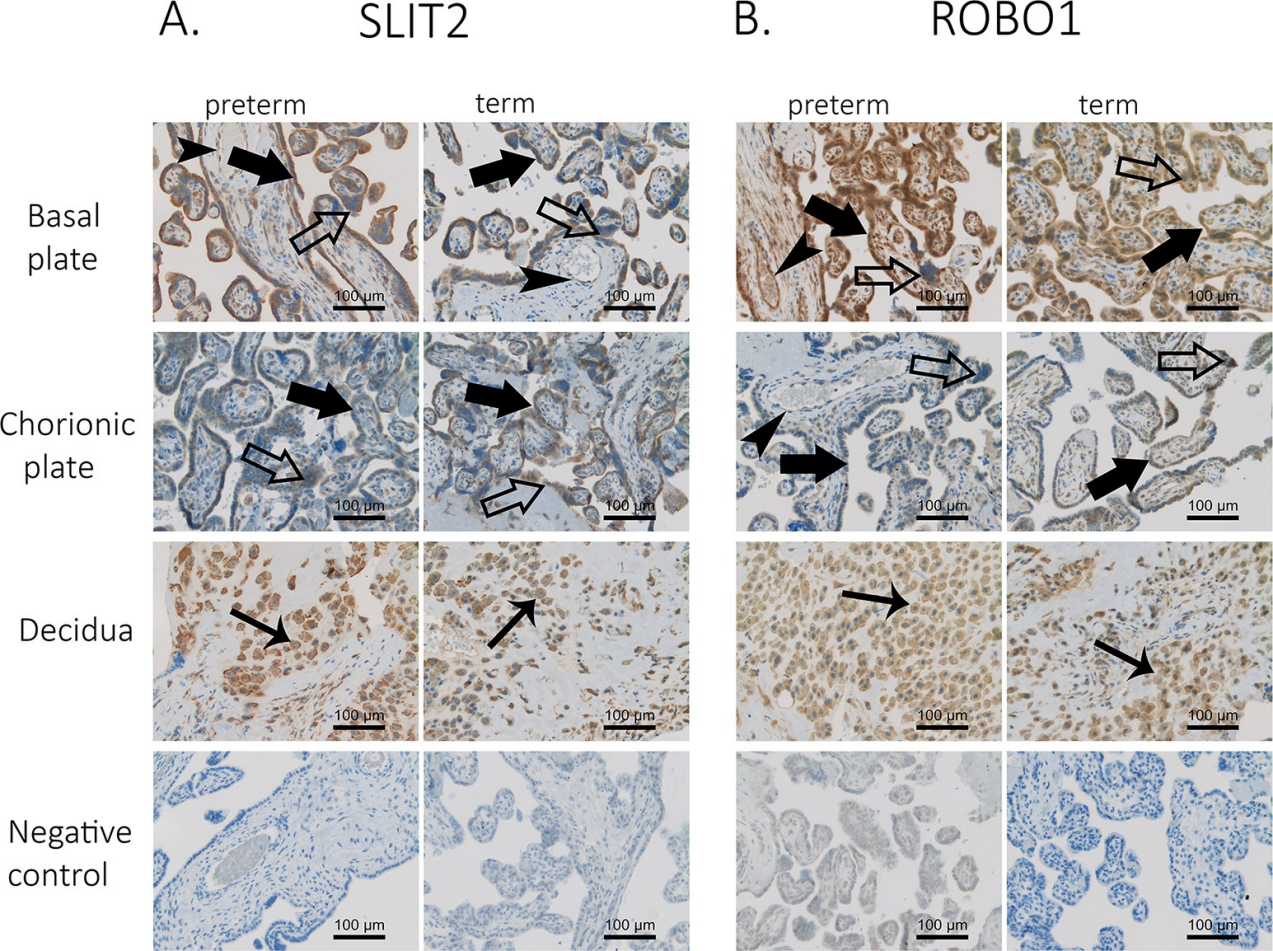
Placental localization of SLIT2 and ROBO1 in spontaneous preterm and term births. Samples from 18 placentas were immunostained with anti‐human SLIT1 (A) and ROBO1 (B) antibodies. Twelve of the placentas were from SPTB deliveries, and six were from spontaneous term deliveries. Two samples from each placenta were stained; one sample from the basal plate (maternal side) and the other from the chorionic plate (fetal side). Immunostaining is indicated by filled large arrows in cytotrophoblasts, unfilled large arrows in syncytiotrophoblasts, filled small arrows in decidual trophoblasts, and filled arrowheads in capillary endothelial cells. Original magnification is 20× in all figures. Control represents isotype controls for immunostaining. Scale bar, 100 μm.

We detected suggestive association signals for SNPs in a region that encompasses *SLIT2* and in a region downstream of *ROBO1*. Protein Slit2 binds to Robo proteins specifically and with high affinity [23,24]. Therefore, we analyzed the localization of SLIT2 and its receptor ROBO1 in human placenta by immunohistochemical staining of placentas from SPTB and spontaneous term birth (STB) with anti‐human SLIT2 and ROBO1 antibodies (Fig 3). Both SLIT2 and ROBO1 localized to cytotrophoblasts, syncytiotrophoblasts, and decidual trophoblasts. In addition, we observed strong ROBO1 and faint SLIT2 staining in capillary endothelial cells. We also detected both proteins in the basal and chorionic plates of the placenta. We did not see apparent differences in staining intensities or cellular localization between placentas from SPTB and STB. This indicates that *SLIT2* and *ROBO1* are expressed in the placenta at the interface between mother and fetus during pregnancy.

### *SLIT2* and *ROBO1* mRNA level elevation in SPTB placentas

Immunohistochemistry demonstrated SLIT2 and ROBO1 in different types of trophoblasts in human placenta (Fig 3). To obtain more quantitative data about placental expression of these proteins, we analyzed *SLIT2* and *ROBO1* mRNA levels by qRT-PCR in samples collected from the basal and chorionic plates of placentas from SPTB (*n* = 23), STB (*n* = 23), and elective preterm birth (EPTB) (*n* = 34).

**Fig 4:**
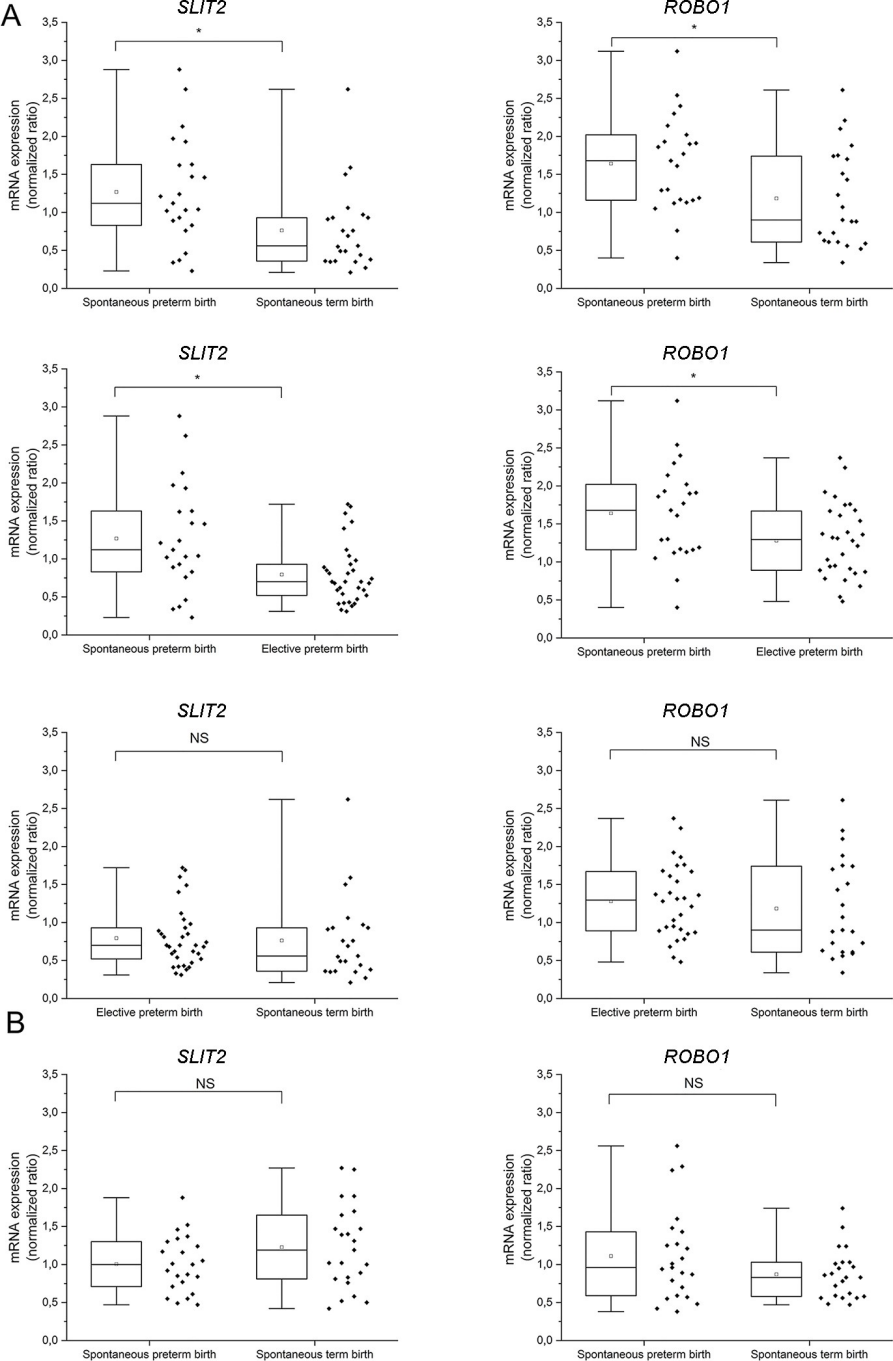
mRNA levels of *SLIT2* and *ROBO1* in spontaneous preterm, spontaneous term, and elective term placentas. Relative mRNA levels from the basal (A) or chorionic (B) plates were normalized to mRNA levels of the housekeeping gene *CYC1*. Differences were analyzed with nonparametric Mann–Whitney *U*‐test. Asterisk indicates statistically significant changes. Box and whiskers display quartiles. Band inside the box is the median, and ends of whiskers show the minimum and maximum values; square inside the box represents the mean value.

We first compared *SLIT2* and *ROBO1* expression levels between SPTB (*n* = 23) and STB (*n* = 23) placentas (Fig 4). Both *SLIT2* and *ROBO1* mRNA levels were higher in the basal plate of SPTB placentas (*SLIT2* fold change [FC] = 1.679, SD = 0.667; *ROBO1* FC = 1.387, SD = 0.670) compared to those of STB (*SLIT2 p* = 0.004, *ROBO1 p* = 0.013; Fig 4A). There were no differences in mRNA levels of *SLIT2* (*p* = 0.173) and *ROBO1* (*p* = 0.297) between the chorionic plates of SPTB and STB placentas (Fig 4B).

To explore the effects of mode of delivery and GA on *SLIT2* and *ROBO1* mRNA levels, we compared SPTB with EPTB placentas. *SLIT2* and *ROBO1* mRNA levels were significantly higher for SPTB (*SLIT2* FC = 1.595, SD = 0.580; *ROBO1* FC = 1.282, SD = 0.577) compared to EPTB (*SLIT2 p* = 0.005, *ROBO1 p* = 0.031) in basal plate samples (Fig 4A). There were no significant differences in these levels in EPTB and STB placentas (*SLIT2 p* = 0.216, *ROBO1 p* = 0.328; Fig 4A). These results suggest that higher *SLIT2* and *ROBO1* expression levels are associated with SPTB.

### Association of *SLIT2* expression with fetal growth

SLIT/ROBO are involved in many processes that involve cell migration, including axon guidance; thus, they could affect trophoblast cell invasion and decidualization. To this end, we looked at whether *SLIT2* or *ROBO1* expression in the basal plate of the placenta is associated with fetal growth. We compared mRNA levels with birth weight-for-GA Z-scores (weight Z-score), which included age and gender standardization of the infants. Deliveries with intrauterine growth restriction or other growth disorders were excluded. *SLIT2* mRNA levels correlated with Z-scores (*p* = 0.023, r_s_ = 0.351) in term and preterm fetuses delivered after spontaneous onset of labor (SPTB and STB samples together, S3 Fig). This suggests that *SLIT2* expression is associated with variations in fetal growth.

### siRNA-induced knockdown of *ROBO1* causes changes in expression levels of inflammation related factors

**Fig 5:**
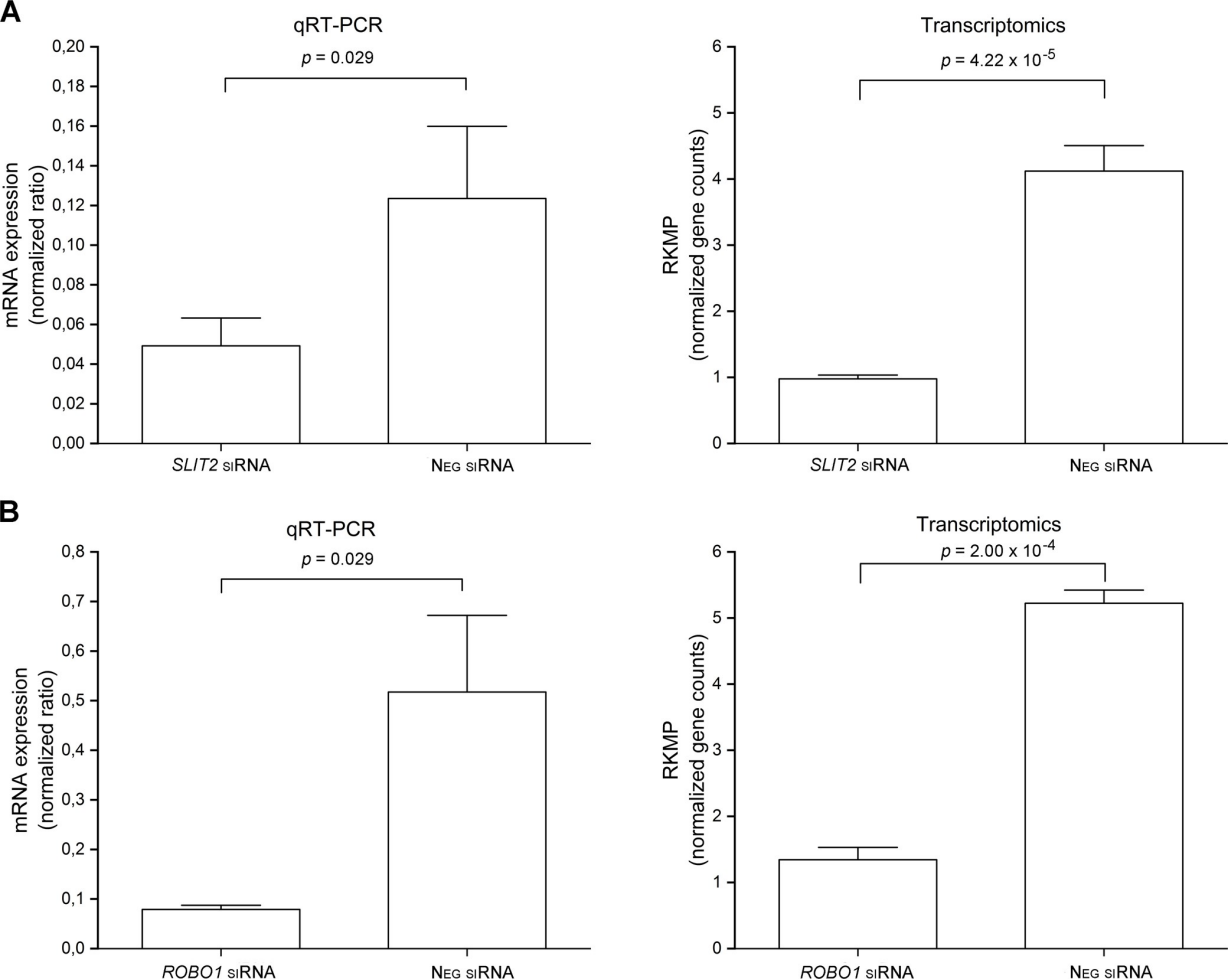
mRNA expression level changes after gene knockdown. *SLIT2* (A) and *ROBO1* (B) were post-transcriptionally knocked down with siRNA in human placental trophoblast continuous cell line HTR8/SVneo. mRNA levels of cells in which *SLIT2* or *ROBO1* were knocked down was compared with mRNA levels from siRNA negative treated control cells. Expression levels of the genes were first determined by qRT-PCR (levels normalized against housekeeping gene *CYC1* mRNA levels). According to qRT-PCR, silencing percentage of *SLIT2* was 60% and silencing percentage of *ROBO1* was 85%. Gene expression levels also determined with high-throughput RNA sequencing. Reads per kilobase of exon per million reads mapped (RKPM) is a normalized gene count value determined in transcriptomic analysis. According to transcriptomics data, silencing percentages were 75% for SLIT2 and 74% for ROBO1. Columns represent median and SD values.

To investigate potential functions of SLIT2 and ROBO1 in placental cells, we silenced *SLIT2* and *ROBO1* expression separately in the HTR‐8/SVneo human trophoblast cell line with small interfering RNAs (siRNAs) (Fig 5). qRT-PCR revealed that silencing percentages were 60% and 85%, for *SLIT2* and *ROBO1*, respectively. Corresponding percentages revealed by RNA sequencing were 75% and 74% for *SLIT2* and *ROBO1*, respectively. Next, we characterized the transcriptomes of trophoblasts in which *SLIT2* or *ROBO1* was silenced, as well as of cells treated with siRNA Universal Negative Control #1. Transcriptomic data analysis identified 14 upregulated (S10 Table) and 12 downregulated (S11 Table) genes after *SLIT2* knockdown compared to samples treated with siRNA Universal Negative Control. The threshold was an FDR-adjusted *p* value of ≤0.01 and an FC of ≥2.0. By the same criteria, there were 216 upregulated (S12 Table) and 610 downregulated (S13 Table) genes after *ROBO1* knockdown. KEGG pathway database analyses (S14 Table, S15 Table) identified the top pathways affected after *SLIT2* and *ROBO1* knockdown as inflammation-related pathways such as cytokine-cytokine receptor interaction (KEGG.ID 4060).

Far fewer genes were affected by *SLIT2* knockdown than by *ROBO1* knockdown, probably because transfection reagent alone upregulated *SLIT2* mRNA levels up to 4-fold (*p* = 0.002) compared to untreated cells. Therefore, knockdown of *SLIT2* expression brought *SLIT2* mRNA levels close to the levels of intact cells. Transfection reagent by itself seemed to activate inflammation-related pathways. The *ROBO1* expression level was not affected by transfection reagent.

*ROBO1* knockdown particularly affected genes encoding membrane receptors and other membrane proteins. KEGG pathway analysis revealed that hematopoietic cell lineage (KEGG ID 4640) had the lowest *p* value (6.57 × 10^−8^) (S15 Table). *ROBO1* knockdown affected 14 of the 42 genes in this pathway: *KIT*, *IL7R*, *IL1R1*, *HLA-DRB1*, *IL1A*, *ITGB3*, *TFRC*, *ANPEP*, *IL1B*, *CD22*, *CD24*, *KITLG*, *CSF3*, and *CD14*. *SLIT2* (FC = 2.0, *p* = 0.005) was among the genes upregulated after *ROBO1* knockdown (S12 Table). One of the gene families highly affected by *ROBO1* knockdown was pregnancy-specific glycoproteins (*PSG*), a complex gene family that regulates maternal–fetal interactions [25]. Of the ten protein-coding human *PSG* genes, six were upregulated after *ROBO1* knockdown. These data indicate that ROBO1 is an important regulator of the HTR8/SVneo cell transcriptome and suggest a role for ROBO1 in modulation of *PSG* gene expression. In addition, ROBO1 appears to have immunomodulatory functions in trophoblast-derived cells.

To verify our suggestive findings from the RNA sequencing data, we used qRT-PCR to analyze the effect of *ROBO1* knockdown on expression levels of selected genes from a larger number of specimens. Because many inflammation‐related pathways were involved, we investigated *TGFA*, *CXCL6*, and total *PSG* expression levels (Table 5). *TGFA* was downregulated after *ROBO1* knockdown, while *CXCL6* was downregulated by both *ROBO1* and *SLIT2* knockdown. Select members of the *PSG* family were upregulated when *ROBO1* was silenced (Table 5): *PSG1*, *PSG2*, *PSG4*, *PSG6*, *PSG7*, and *PSG9*. To verify, we measured total mRNA expression of different *PSG*s (*PSG1*, *PSG2*, *PSG3*, *PSG4*, *PSG5*, *PSG6*, *PSG7*, *PSG8*, *PSG9*, and *PSG11*) at the same time in one qRT-PCR reaction, as described previously [26]. We also tested the effect of knockdown on *IL6*, *TNFA*, and *SRGAP3* (Table 5).

**Table 5:** Comparison of RNA sequencing and qRT-PCR of *ROBO1* and *SLIT2* genes affected by knockdown. Gene expression changes after *SLIT2/ROBO1* knockdown were verified in a larger number of specimens (*n* = 6) by qRT-PCR. SLIT2 and ROBO1 were silenced in HTR8/SVneo commercial cell line with siRNAs. mRNA levels of SLIT2/ROBO1-silenced cells were compared with mRNA levels of cells treated with negative siRNA. In addition to genes affected by gene knockdown according to RNA sequencing data, we tested the effect of knockdown on mRNA expression of well-known cytokines *IL-6* and *TNF-A*.

Gene		RNA seq: ROBO1 knockdown		RNA-seq: SLIT2 knockdown		qRT-PCR: ROBO1 knockdown		qRT-PCR: SLIT2 knockdown	
ID	Description	FC^a^	*p*–value^b^	FC^a^	*p*–value^b^	FC^a^	*p*–value^c^	FC^a^	*p*–value^c^
***ROBO1***	roundabout guidance receptor 1	-3.83	<0.001	1.15	0.0607	-7.56	0.002	-1.08	0.699
***SLIT2***	slit guidance ligand 2	2.05	0.004	-4.00	<0.001	1.03	0.699	-3.99	0.002
***TGF-A***	transforming growth factor alpha	-2.07	0.004	1.35	0.014	-1.81	0.041	1.24	0.132
***CXCL-6***	C-X-C motif chemokine ligand 6	-2.30	<0.001	-2.41	0.010	-2.74	0.002	-2.94	0.002
***SRGAP3***	SLIT-ROBO Rho GTPase activating protein 3	-2.19	0.004	-1.03	0.883	-1.73	0.065	-1.13	0.589
***PSG1***	pregnancy specific beta-1-glycoprotein 1	5.05	<0.001	1.30	0.436				
***PSG2***	pregnancy specific beta-1-glycoprotein 2	5.90	0.005	-1.54	0.266				
***PSG4***	pregnancy specific beta-1-glycoprotein 4	2.26	0.004	-1.06	0.526				
***PSG5***	pregnancy specific beta-1-glycoprotein 5	1.40	0.009	-1.01	0.963				
***PSG6***	pregnancy specific beta-1-glycoprotein 6	10.34	0.004	1.32	0.576				
***PSG7***	pregnancy specific beta-1-glycoprotein 7	2.65	0.005	1.45	0.128				
***PSG9***	pregnancy specific beta-1-glycoprotein 9	2.18	0.004	-1.30	0.261				
***total PSG***	total expression of pregnancy specific beta-1-glycoproteins					1.71	0.015	-1.14	0.310
***IL-6***	interleukin 6	-1.16	0.072	-1.54	0.014	-1.38	0.041	-1.53	0.002
***TNF-A***	tumor necrosis factor					-4.44	0.002	-1.06	0.818

^a^Expression ratio (fold-change) between compared sample groups. Comparison between ROBO1/*SLIT2-*silenced cells and siRNA negative–treated control cells.

^b^FDR-adjusted *p* value for comparisons between sample groups (*ROBO1/SLIT2*-silenced and negative-control cells).

The results of qRT-PCR were generally in line with those of transcriptome sequencing (Table 5). Knockdown of *ROBO1* downregulated mRNA expression of *TGFA* (S4A Fig). Knockdown of either *ROBO1* or *SLIT2* downregulated *CXCL6*, whereas *PSG*s were upregulated by *ROBO1* knockdown, similar to the results of RNA sequencing. Inflammatory cytokines *IL6* and *TNFA* were both downregulated after *ROBO1* knockdown (S4B Fig). *IL6* was also downregulated after *SLIT2* knockdown. There was a trend toward downregulation of *SRGAP3* by *ROBO1* knockdown. Thus, the RNA sequencing and qRT-PCR results are in accordance and confirm that *ROBO1* knockdown affects the expression of immune response–modifying genes in a cell culture model.

## Discussion

Both maternal and fetal genetic factors contribute to SPTB. Nevertheless, variants in the fetal genome associated with SPTB predisposition are not well known. Our aim was to identify fetal genetic factors associated with SPTB. To this end, we performed a case–control GWAS study in a Finnish population that is known to be relatively genetically homogeneous. Based on these results, we identified a *SLIT2* variant as a plausible factor for SPTB susceptibility. This led to further investigations to define high-risk populations and characterize *SLIT2* and *ROBO1* expression in the placenta and in trophoblast cells.

The association of the *SLIT2* variant with SPTB was strongest in the population of very premature births (<32 weeks gestation), and this association was replicated in a European population with SPTB fetuses from 24 to 30 weeks gestation. We detected association signals for SNPs in a region that encompassed *SLIT2* and for SNPs in a region downstream of *ROBO1*, which encodes the receptor for SLIT2. *SLIT2* and *ROBO1* encode proteins of the SLT2-ROBO1 signaling pathway; previous studies have indicated that this pathway is associated with different types of pregnancy complications, including preeclampsia [27,28], impaired placentation of missed and threatened miscarriage in early pregnancy [29], trophoblast invasion, and vascular remodeling during ectopic tubal pregnancy [29,30]. Our hypothesis-free GWAS study provides evidence that these genes have a role in another pregnancy complication, SPTB.

We did not detect associations that would reach the stringent level of genome-wide significance (*p*<10^**−8**^). This may be due to a relatively small sample size in the GWAS, which was one of the limitations in the study. However, we did identify signals below the generally used threshold of suggestive association (*p* <10^**−5**^). Moreover, the SPTB-associated SNPs in the *SLIT2* locus showed association in an independent data set. In the future, there is a need to validate our findings in larger data sets to detect signals that may have gone undetected in the current sample size.

Previous study presented that fetal *de novo* mutations in genes that are involved in brain development are associated with preterm birth [31]. In line with this notion, SLIT2-ROBO1 signaling has a well-documented role in axon guidance during the development of the nervous system [32]. Therefore, it appears that at least in part the same fetal genetic factors are involved both in the onset of preterm birth and in brain development. It is known that preterm birth increases the risk of compromised brain development [33,34]. How much of the risk can be explained by shared genetic risk factors remains to be determined.

KEGG pathway analysis of GWAS data showed that the top-ranking pathway was SLIT2/ROBO1 signaling–regulated axon guidance. Previous studies have indicated that brain and placental development may share common pathways [35–37]. Although SLIT and ROBO were originally identified as axon guidance cues, they interact in many other cellular processes, including regulation of cell migration, cell death, and angiogenesis. As such, they have an essential role in the development of tissues such as lung, liver, kidney, breast, and tissues of the reproductive system [38,39].

Trophoblasts cover a large portion of the placenta and have multiple roles in the maintenance of pregnancy. Invading trophoblasts have a critical function in biogenesis of the placenta [40,41]. Later during pregnancy, decidual trophoblasts may have a role in silencing immune cells in the decidua. The lining of placental villae consists of the syncytiotrophoblast layer and cytotrophoblasts, which have roles ranging from immune protection to uptake of nutrients from maternal blood [42]. In addition to their functions in biogenesis of the placenta and maintenance of pregnancy, trophoblasts may participate in labor induction [43]. Two‐thirds of early pregnancy failures may present with reduced trophoblast invasion [29]. SLIT-ROBO signaling may play autocrine and/or paracrine roles in trophoblast functions, such as differentiation and invasion, by influencing the migration of trophoblastic cells [29,44]. Thus, SLIT2-ROBO1 signaling may be involved in the pathogenesis of pregnancy failures *via* their effect on trophoblastic cell functions. Indeed, our immunohistochemical experiments demonstrated that villous and decidual trophoblasts from preterm and term placentas were strongly positive for SLIT2 and ROBO1. These results are in line with those of a previous study, which demonstrated that villous syncytiotrophoblasts express high levels of SLIT2 and ROBO1. The study also found that trophoblastic endothelial cells highly coexpress multiple SLIT ligands (SLIT2, SLIT3) and ROBO receptors (ROBO1, ROBO2, and ROBO4) in full-term placenta. Thus, SLIT-ROBO signaling may also have an important role in the regulation of normal placental functions [27].

An earlier study found that levels of several *SLIT*/*ROBO* mRNAs and proteins are higher in preeclamptic placentas compared to normal controls [27]. Our data indicate that mean *SLIT2* and *ROBO1* mRNA levels were higher in SPTB placentas compared to placentas from spontaneous term deliveries and to placentas from elective preterm births. Consequently, increased levels of *SLIT2* and *ROBO1* mRNAs were associated with SPTB. In addition, *SLIT2* may have a role in term spontaneous labor, as *SLIT2* mRNA and protein expression are decreased in the myometrium after spontaneous term labor [45].

In addition to the associations of both the *SLIT2* variant and mRNA expression of *SLIT2* and *ROBO1* in basal plate of placenta with SPTB, *SLIT2* mRNA levels in placentas were associated with the birth weight of fetuses born after spontaneous labor. A GWAS of beef cattle identified *SLIT2* as a candidate gene that affects the weight of internal organs [46]. As the fetal growth and intrauterine distention negatively associates the duration of pregnancy [47] influence of *SLIT2* on fetal size is a potential mechanism that remains to be studied as a cause of SPTB.

To understand more about the function of *SLIT2* and *ROBO1* in trophoblast cells, we silenced their expression separately in immortalized extravillous invading trophoblasts. Altogether, 26 and 826 genes were affected by *SLIT2* or *ROBO1* siRNA knockdown, respectively. The low number of genes affected by *SLIT2* knockdown was probably because the transfection reagent alone upregulated *SLIT2* mRNA levels compared to untreated cells. Consequently, knockdown of *SLIT2* expression only brought *SLIT2* mRNA levels back to the levels of control cells. However, the mRNA expression level of *ROBO1* was not affected by the transfection reagent. Genes affected by ROBO1 knockdown were mostly related to infection, inflammation, and immune response. These results correspond with those of previous studies, suggested that members of the SLIT and ROBO families act as regulators of the inflammatory response [45,48]. Both pro‐inflammatory [45,49,50] and anti‐inflammatory [48,51] functions have been reported. Our results from invading trophoblast cells support a proinflammatory role for SLIT2-ROBO1 signaling, since the genes downregulated by *ROBO1* knockdown included proinflammatory cytokines and chemokines such as *IL1A*, *IL1B*, *CXCL8*, *CCL2*, and *CXCL6*. It is widely acknowledged that IL1 in particular, as well as other proinflammatory cytokines and chemokines, is associated with preterm labor [52–54]. IL1B is a primary secretory product of the inflammasome and as such is thought to have central roles in initiation of preterm labor, such as in the induction of prostaglandin synthesis. Both polymorphisms of *IL1A* and *IL1B*, as well as increased levels of IL1B, are associated with preterm birth [19,54–56]. CXCL6 is increased in amniotic fluid from patients with preterm labor complicated by intra-amniotic inflammation and from patients with SPTB without intra-amniotic infection/inflammation [57]. We propose that in trophoblast cells *ROBO1* has a role in regulation of proinflammatory mediators. Genes involved in vascular formation (vasculogenesis) or development (angiogenesis) were not affected by *SLIT2* or *ROBO1* knockdown in trophoblasts.

The PSG family was one of the immune response–associated gene families affected by *ROBO1* knockdown. PSGs include ten placental trophoblast–synthetized glycoproteins that belong to the immunoglobulin superfamily [58]. Of the six *PSG*s upregulated by siRNA-induced knockdown, *PSG1* was ranked among the top three upregulated genes (S12 Table). PSGs are essential in the maintenance of normal pregnancy [58]; thus, altered *PSG* expression patterns could influence pregnancy complications. Over the years, complications such as abortion, preeclampsia, intrauterine growth retardation, fetal distress, and preterm delivery have all been linked to low PSG levels [58–63]. As *ROBO1* was upregulated in SPTB placentas and knockdown of *ROBO1* upregulated expression of *PSG* genes, we propose that ROBO1 signaling is important in downregulation of the expression of *PSG*s. In addition, PSG1 activates TGF-B 1 and TGFB2 [25,64,65]; *TGFB1* suggestively associated with SPTB [13] and TGFB2 prevented preterm birth in experimental inflammatory stress [66].

The innate immune response and inflammation contribute to labor and delivery, particularly in preterm pregnancies [3,22,67,68]. Upregulation of proinflammatory cytokines stimulates and potentiates uterine contractions in the myometrium [69–71]. In preterm labor and delivery, it is mostly inflammatory signals that spread to the placenta, fetal membranes, and fetal compartment. It is plausible that *SLIT2* and *ROBO1* expressed by trophoblasts are associated with SPTB via regulation of inflammation-related factors. *SLIT2*-*ROBO1*–guided activation and propagation of inflammatory mediators throughout the fetal–maternal trophoblast interface of the uterine wall would likely influence the tissues actively involved in labor and delivery. As knockdown of *ROBO1* downregulated many of the genes that encode cytokines and chemokines, it is probable that upregulation of *ROBO1* in SPTB placentas compared to term placentas would also affect expression of these genes. There is both epidemiological and experimental evidence that untimely expression of cytokines and chemokines by either fetal or maternal tissues upregulates the activity of mediators, which leads to premature initiation of the parturition process [72].

In conclusion, the GWAS detected fetal association signals for SPTB and duration of pregnancy in the vicinity of *SLIT2* and *ROBO1*. *SLIT2* and *ROBO1* were upregulated in SPTB placentas, and further functional studies confirmed that this signaling pathway has a role in regulation of the pathways associated with infection, inflammation, and immune response in trophoblast-derived cells. These results suggest that *SLIT2* and *ROBO1* play specific roles in increasing susceptibility to SPTB. SLIT2-ROBO1 signaling is associated with complications in early pregnancy and it is possible that it influences invading trophoblasts during placentation. Based on the currently available evidence, we propose that activation of *SLIT2-ROBO1* expression and signaling in trophoblast cells contributes to inflammatory and immune activation, which in turn leads to early labor and preterm birth.

## Materials and methods

### Ethical approval

The present studies received ethical approval from the participating centers (Oulu University Hospital 79/2003, 14/2010, and 73/2013). Informed consent was obtained from study participants or their parents.

### Study populations in genetic studies

Characteristics of the Finnish study populations are summarized in S1 Table. The discovery GWAS study population consisted of singleton SPTB and term infants sampled in Oulu and Tampere University Hospitals. The study subjects were recruited prospectively during 2004–2014 and retrospectively from the 1973–2003 birth diaries of Oulu University Hospital. For replication, we downloaded the summary statistics of a European population described in a recent study by Rappoport *et al*. [20] through ImmPort (http://www.immport.org/: SDY1205, DOI: 10.21430/M37N6PJEQT). This population includes 260 SPTB cases (139 male and 121 female infants) and 9,630 controls (4,055 males and 5,575 females). The cases were very preterm infants born between 25 and 30 weeks of gestation and were clinically defined as SPTB in 2005–2008. The control population consisted of adults, originally from the Health and Retirement Study (HRS) [73], who were matched for ethnicity with the European cases.

In the Finnish cohorts, SPTB was defined as birth occurring after spontaneous onset of labor at <36 completed weeks + 1 day of gestation. All medically indicated preterm births and deliveries that included known major risk factors were excluded. These criteria led to exclusion of preterm deliveries that involved the following conditions or characteristics: multiple gestation, preeclampsia, intrauterine growth restriction, placental abruption, polyhydramnios, fetuses with anomalies, clinical chorioamnionitis or acute septic infection in the mother, diseases in the mother that could influence timing of delivery, alcohol/narcotic use, and accidents. Term birth was defined as birth occurring at 38–41 weeks (38 wk + 0 d to 41 wk + 6 d) of gestation. The following conditions were used as exclusion criteria for the control population: multiple gestation, intrauterine growth restriction, placental abruption, polyhydramnios, fetuses with anomalies, and requirements for special care of the newborn. All control infants were from families with at least two term deliveries without any preterm deliveries in the family.

### DNA sample preparation, genotyping, and SNP inclusion criteria

Umbilical cord blood, umbilical cord tissue, or saliva was obtained from the study subjects. Commercial kits were used to extract genomic DNA from blood (UltraClean Blood DNA Isolation Kit; MO BIO Laboratories, Inc., Carlsbad, CA, USA or Puregene Blood Core Kit; Qiagen, Hilden, Germany) and cord tissue (Gentra Puregene Tissue Kit, Qiagen). OraGene DNA collection kits (DNA Genotek, Ontario, Canada) were used for collecting saliva, and DNA was extracted with the prepIT-L2P kit (DNA Genotek). Genome-wide SNP genotyping was performed with the Infinium HumanCoreExome BeadChip (Illumina, San Diego, CA, USA) by the Technology Centre, Institute for Molecular Medicine Finland (FIMM), University of Helsinki.

### Processing of genome-wide SNP data

Genome-wide SNP data were processed with PLINK, v. 1.9 [74]. SNPs with minor allele frequency (MAF) < 0.01, genotyping rate < 0.9, or deviation from Hardy–Weinberg equilibrium (*p* < 0.0001) were excluded. Individuals with > 0.1 missing genotypes were excluded. Identical by descent (IBD) clustering and multidimensional scaling (MDS) analyses were performed with a linkage disequilibrium–pruned SNP set; population outliers and close relatives (pihat > 0.2) were excluded. Prephasing of genotypes was performed with SHAPEIT2 [75], followed by statistical imputation with IMPUTE2 [76] using the 1000 Genomes Phase 3 variant set (October 2014) as the reference panel. Before association analysis, SNPs with impute info score < 0.8 or MAF < 0.05 in cases or controls were excluded. Altogether 6,778,521 SNPs or short insertions/deletions remained for analysis after these quality control steps.

### Statistical analyses in genetic studies

Associations between SPTB or GA and SNPs were assessed with the frequentist test under the additive model with SNPtest, v. 2.5.2 [77]. After the primary analysis, the following subgroups of SPTB infants were assessed: (1) very preterm infants (GA 23–31 wk + 6 d), (2) moderate-to-late SPTB infants (GA 32 wk + 0 d to 36 wk + 0 d), (3) PPROM before onset of labor, and (4) no PPROM before onset of labor. To account for population substructure in the GWAS, the first two MDS dimensions were included as covariates. In the GWAS, the effect of population stratification was minimal (λ = 1.03). Gene set analysis (GSA)-SNP was used to search for gene set enrichment in pathway analysis [78]. We included only genotyped SNPs located within genes in this analysis to avoid the complicating effects of SNPs in linkage disequilibrium. R, v. 3.2.2 (https://www.r-project.org) was used to create Manhattan plots. LocusZoom [79] was used to create regional association plots.

### Functional annotation of SNPs

We annotated SNPs with three approaches: (1) We used Genotype-Tissue Expression (GTEx) data to analyze whether the SNPs overlap with *cis* expression quantitative trait loci (eQTLs) [80]; 2) we screened whether the SNPs had been associated with any phenotypes in previous GWA studies using the GWAS catalog[81]; and (3) we assessed whether the SNPs were located within putative regulatory regions using HaploReg, v. 4.1 [82].

### Placental samples for immunohistochemistry and qRT-PCR

Samples from human placenta were collected at Oulu University Hospital during 2010–2016 as described [16]. The placental samples used in immunohistochemical staining and in qRT-PCR analysis of *SLIT2* and *ROBO1* expression were subject to similar inclusion criteria as the samples used in GWAS. The inclusion criteria of gestational age for preterm placental samples was from 25 weeks to 36 weeks+ 6 days and 39 weeks to 41 weeks + 6 days for term samples. The same conditions (multiple gestation, intrauterine growth restriction, placental abruption, polyhydramnios, fetuses with anomalies or requirements for special care of the newborn) as in GWAS were used as exclusion criteria for term controls. Spontaneous preterm samples had almost the same exclusion criteria except the population included few cases with chorioamnionitis or oligohydramnion. The control group of elective preterm samples included cases with various pregnancy complications like IUGR or pre-eclampsia resulting in elective preterm delivery without labor.

Specifically, in total, 18 placental samples were analyzed by immunohistochemistry. Twelve samples were from SPTB deliveries (GA from 25 wk + 2 d to 35 wk + 2 d), and six were from spontaneous term deliveries (GA from 39 wk + 4 d to 41 wk +1 d). Samples from both basal and chorionic plates were included in the study.

RT-qPCR was performed with 23 placental samples from SPTB (GA from 25 wk + 2 d to 36 wk + 0 d), 34 from elective preterm birth (EPTB) (GA from 25 wk + 1 d to 36 wk + 6 d), and 23 from spontaneous term birth (STB) (GA from 39 wk + 1 d to 41 wk + 6 d).

### Sample preparation and immunohistochemical staining

Localization of encoded proteins was visualized in placental tissues by immunohistochemical staining. Samples were embedded in paraffin and cut into 4-μm slices, deparaffinized, and rehydrated. Antigen retrieval was done in Tris-EDTA buffer. Endogenous peroxidase activity was blocked in blocking solution (Agilent, Santa Clara, CA, USA). Samples from the chorionic plate were incubated with mouse anti-human SLIT2 antibody (1:4000 dilution, PA5-3113; ThermoFisher Scientific, Waltham, Massachusetts, USA) or mouse anti-human ROBO1 antibody (1:2000, PA5-34931; ThermoFisher Scientific). Samples from the basal plate of the placenta were incubated in a 1:5000 dilution of mouse anti-human SLIT2 antibody and 1:1000 dilution of mouse anti-human ROBO1 antibody. Bound antibodies were detected with the Envision kit (Agilent).

### Quantitative PCR

Tissue samples were homogenized, RNA was isolated with the RNeasy Mini Kit (Qiagen), and cDNA was synthetized as described previously [16]. After the RT-PCR, cDNA samples were diluted 1:2 using Rnase-free H_2_0.

*SLIT2* and *ROBO1* were relatively quantified by intron spanning assays with Light-Cycler96 (Roche Diagnostics, Risch-Rotkreuz, Switzerland) and cytochrome c-1 (*CYC1*) as a reference gene. *CYC1* was chosen as a reference gene because it is one of the most stably expressed genes in the placenta [16,83–85]. Primers and probes were: forward 5′-CTTCCAGAGACCATCACAGAAA-3′ and reverse 5′-CGTCTAAGCTTTTTATATGGTGAGAA-3′ for *SLIT2* (with UPL probe #79), forward 5′-CGCAGAGAAACCTACACAGATG-3′ and reverse 5′-GGATTGGGCAGTAGGTGACT-3′ for *ROBO1* (with UPL probe #31), and forward 5′-ATAAAGCGGCACAAGTGGTCA-3′ and reverse 5′-GATGGCTCTTGGGCTTGAGG-3′ for *CYC1* (with UPL probe #47). Probes were from the Universal Probe library (UPL) Set (Roche Diagnostics). Each qPCR measurement was done in triplicate. Levels of *SLIT2* and *ROBO1* were normalized against the *CYC1* level, and relative quantifications were then assessed with the ΔΔ cycle threshold method. A few randomly chosen qPCR products were also verified by agarose gel electrophoresis and Sanger sequencing.

Primers and probes genes for transforming growth factor alpha (*TGFA*), C-X-C motif chemokine ligand 6 (*CXCL6*), and SLIT-ROBO Rho GTPase activating protein 3 (*SRGAP3*) *w*ere: forward 5′-CCCTGGCTGTCCTTATCATC-3′ and reverse 5′-GGCACCACTCACAGTGTTTTC-3′ for *TGFA* (with UPL probe #74), forward 5′-CCAGAAAATTTTGGACAGTGG-3′ and reverse 5′-GGGATCTCCAGAAAACTGCTC-3′ for *CXCL6* (with UPL probe #61), and forward 5′-GAAGGGCACTCGATGAGGT-3′ and reverse 5′-GCTCATGGTCTTCTCGATGTC-3′ for *SRGAP3* (with UPL probe #66).

Total mRNA expression of different *PSG*s was measured with PCR primers: forward 5′-CCTCTCAGCCCCTCCCTG-3′ and reverse 5′-GGCAAATTGTGGACAAGTAGAAGA-3′ (with UPL probe #15), which are complementary to sequences conserved in all but two *PSG* transcript variants that both lack the N domain [26]. *IL6* and *TNFA* were analyzed with primers forward 5′-GCCCAGCTATGAACTCCTTCT-3′ and reverse 5′-GCGGCTACATCTTTGGAATC-3′ for *IL-6* (with UPL probe #43) and forward 5′-CAGCCTCTTCTCCTTCCGAT-3′ and reverse 5′-GCCAGAGGGCTGATTAGAGA-3′ for *TNFA* (with UPL probe #40).

### Gene knockdown of *SLIT2* and *ROBO1* by transfection with small interfering RNAs (siRNAs)

Human placental trophoblast cells HTR-8/SVneo (CRL-3271™; ATCC, Manassas, Virginia, USA). were grown in RPMI-1640 culture media (Thermo Fisher Scientific) supplemented with 10% fetal bovine serum (FBS; Sigma-Aldrich, St. Louis, MO, USA) and 1× penicillin/streptomycin (Sigma-Aldrich). Cells were cultured under standard culturing conditions (37°C, 5% CO_2_, humidified atmosphere), and subculturing was performed with 0.05% trypsin/0.02% EDTA.

siRNAs targeting *SLIT2* (s GUCAUAUCAAGAACUGUGAdTdT, a UCACAGUUCUUGAUAUGACdTdT) and *ROBO1* (s CAUACCUAUGGCUACAUUUdTdT, a AAAUGUAGCCAUAGGUAUGdTdT) (Sigma-Aldrich) were reverse transfected and then forward transfected in HTR-8/SVneo cells with Lipofectamine 3000 reagent (Invitrogen, Carlsbad, CA, USA) [86]. MISSION siRNA Universal Negative Control #1 (Sigma-Aldrich) was used as a negative control for siRNA transfection and was transfected in the same manner as siRNAs targeting *SLIT2* and *ROBO1*. In the reverse transfection, the cells (70,000 cells/well) were incubated with siRNAs at a final concentration of 30 nM. The cells were transfected again after 24 h of incubation. The second transfection was done as a forward transfection in the presence of 40 nM siRNAs. Cells were incubated with siRNAs for 24 h after the second transfection, and then fresh medium was added and cells were incubated for an additional 24 h. Cells were harvested with 1× Trypsin-EDTA (Sigma-Aldrich).

### Transcriptomic analysis of *SLIT2-* and *ROBO1*-silenced HTR8/SVneo cells

Cells were disrupted with a 25 G needle and 1 ml syringe, and RNA was isolated in accordance with the manufacturer’s instructions (RNeasy Micro Kit, Qiagen). The quality of isolated RNA was checked with an Agilent 2100 Bioanalyzer system at the Biocenter Oulu Sequencing Center, Finland.

Samples containing total RNA were sent for transcriptomic analysis to the Finnish Functional Genomics Centre (FFGC; Turku, Finland), where transcriptional profiles of *SLIT2* (*n* = 3) and *ROBO1* (*n* = 3) -silenced cells and negative-control cells (n = 3) were detected with the Illumina HiSeq high‐throughput sequencing system. Whole cell RNA sequencing data was analyzed by the Bioinformatics Unit Core Service at the Turku Centre for Biotechnology, Finland. The transcriptomics data have been deposited in NCBI’s Gene Expression Omnibus [87] and are accessible through GEO Series accession number GSE119101 (https://www.ncbi.nlm.nih.gov/geo/query/acc.cgi?acc=GSE119101)

### qRT-PCR verification of transcriptomic data from *SLIT2*- and *ROBO1*-silenced HTR8/SVneo cells

Knockdown of *SLIT2* and *ROBO1* with siRNAs, total RNA isolation, cDNA synthesis, and qPCR were done as described above, except 30 nM siRNAs were used in the forward transfection instead of 40 nM.

### Statistical analysis of RT-qPCR results

Differences in mRNA expression levels among the phenotypes were assessed by nonparametric Mann–Whitney *U*-test with SPSS Statistics 20.0 (IBM Corporation).

## Supporting information

S1 Fig Manhattan plot showing the results of genome-wide association study of gestational age. Each dot represents the–log_10_(*p*) value of a single SNP in association analysis. Blue line denotes the level of suggestive significance (–log_10_(*p*) > 5). Loci are indicated for regions with suggestive significance.
(TIF)

S2 Fig **Regional association plots for *SLIT2* region in GWAS of SPTB (A) and gestational age (B).** Each dot represents −log_10_(*p*) value of a single SNP in association analysis. Blue line denotes level of suggestive significance (−log_10_(*p*) > 5).
(TIF)

S3 Fig *SLIT2* mRNA expression correlation with Z-score. *SLIT2* mRNA expression in the basal plate of the placenta compared to birthweight-for-gestational age Z-scores of spontaneously delivered infants (SPTB and STB samples together). *SLIT2* mRNA levels correlated with Z-scores (*p* = 0.023).
(TIF)

S4 Fig Gene expression changes after *SLIT2* and *ROBO1* silencing verified by qRT-PCR. (A) Four genes/gene families affected by *ROBO1* silencing according to RNA sequencing were verified by qRT-PCR. (B) Expression changes of inflammation-associated cytokines *IL-6* and *TNF-A* after *SLIT2* and *ROBO1* silencing were also measured with qRT-PCR. *SLIT2* and *ROBO1* were post-transcriptionally silenced in HTR8/SVneo cell line by siRNA. mRNA levels of selected genes compared between *SLIT2-* or *ROBO1*-silenced cells and mRNA levels of untreated control cells. All mRNA levels normalized against mRNA levels of housekeeping gene *CYC1*. Columns represent median and SD values of the sample groups.
(TIF)

S1 Table Clinical characteristics of discovery GWAS study population. (DOCX)

S2 Table Suggestive association signals in GWAS of gestational age. (DOCX)

S3 Table Suggestive association signals in GWAS of very preterm birth. (DOCX)

S4 Table Suggestive association signals in GWAS of moderate-to-late preterm birth. (DOCX)

S5 Table Suggestive association signals in GWAS of infants born after SPTB with PPROM. (DOCX)

S6 Table Suggestive association signals in GWAS of infants born after onset of preterm labor with intact fetal membranes. (DOCX)

S7 Table Pathways associated with spontaneous preterm birth. (DOCX)

S8 Table Gene ontology terms associated with SPTB. (DOCX)

S9 Table Significant gene ontology terms that include *SLIT2* and *ROBO1* genes. (DOCX)

S10 Table Upregulated genes after *SLIT2* silencing in HTR8/SVneo cells. *SLIT2* silenced in HTR8/SVneo commercial cell line by siRNA. Transcriptome of these cells compared with transcriptome of cells treated with negative siRNA. Differentially expressed genes ranked based on FDR-adjusted *p* value and fold change (FC). Threshold of fold change was > 2.0, and threshold of FDR-adjusted *p* value was <0.05.
(DOCX)

S11 Table Downregulated genes after *SLIT2* silencing in HTR8/SVneo cells. *SLIT2* silenced in HTR8/SVneo commercial cell line by siRNA. Transcriptome of these cells compared with transcriptome of cells treated with negative siRNA. Differentially expressed genes ranked based on FDR-adjusted *p* value and fold change. Threshold of fold change was > 2.0, and threshold of FDR-adjusted *p* value was <0.05.
(DOCX)

S12 Table Upregulated genes after *ROBO1* silencing in HTR8/SVneo cells. *ROBO1* silenced in HTR8/SVneo cell line by siRNA. Transcriptome of these cells compared with transcriptome of cells treated with negative siRNA. Differentially expressed genes ranked based on FDR-adjusted *p* value and fold change. Threshold of fold change was > 2.0, and threshold of FDR-adjusted *p* value was <0.05.
(DOCX)

S13 Table Downregulated genes after *ROBO1* silencing in HTR8/SVneo cells. *ROBO1* silenced in HTR8/SVneo cell line by siRNA. Transcriptome of these cells compared with transcriptome of cells treated with negative siRNA. Differentially expressed genes ranked based on FDR-adjusted *p* value and fold change. Threshold of fold change was > 2.0, and threshold of FDR-adjusted *p* value was <0.01.
(DOCX)

S14 Table Biological pathways affected by *SLIT2* silencing in HTR8/SVneo cells. (DOCX)

S15 Table Biological pathways affected by *ROBO1* silencing in HTR8/SVneo cells. (DOCX)
