# Supplementary figures and images for: Risk of spontaneous preterm birth and fetal growth associates with fetal SLIT2
Source: PLoS Genet. 2019 Jun 13;15(6):e1008107. doi: 10.1371/journal.pgen.1008107 (PMC6563950; doi:10.1371/journal.pgen.1008107)

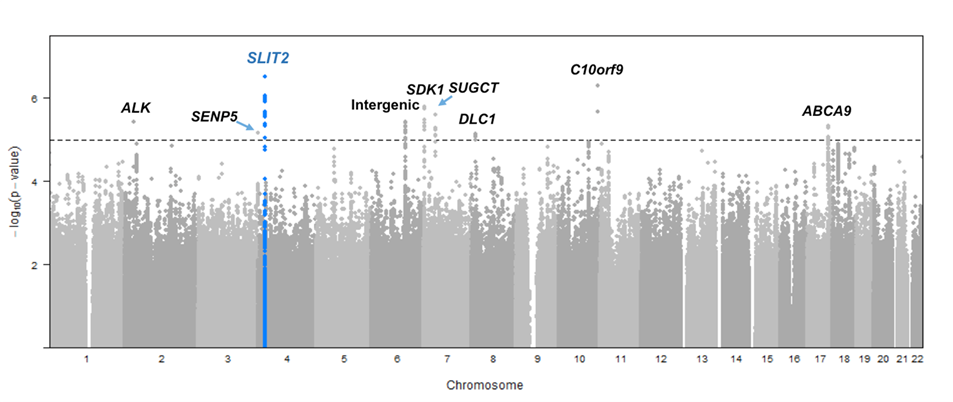

Supplement: S1 Fig — Each dot represents the–log10(p) value of a single SNP in association analysis. Blue line denotes the level of suggestive significance (–log10(p) > 5). Loci are indicated for regions with suggestive significance. (TIF) [file pgen.1008107.s001.tif]

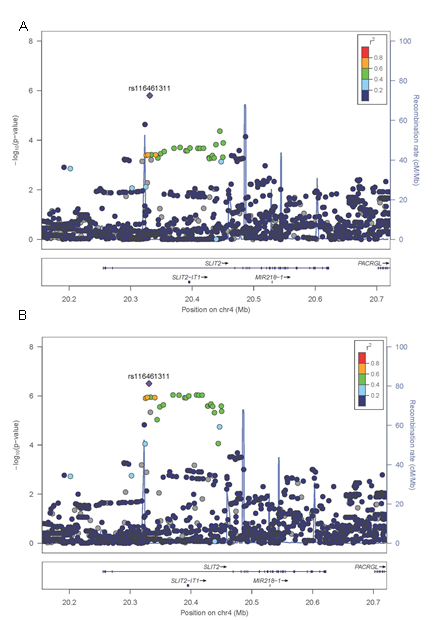

Supplement: S2 Fig — Regional association plots for SLIT2 region in GWAS of SPTB (A) and gestational age (B). Each dot represents −log10(p) value of a single SNP in association analysis. Blue line denotes level of suggestive significance (−log10(p) > 5). (TIF) [file pgen.1008107.s002.tif]

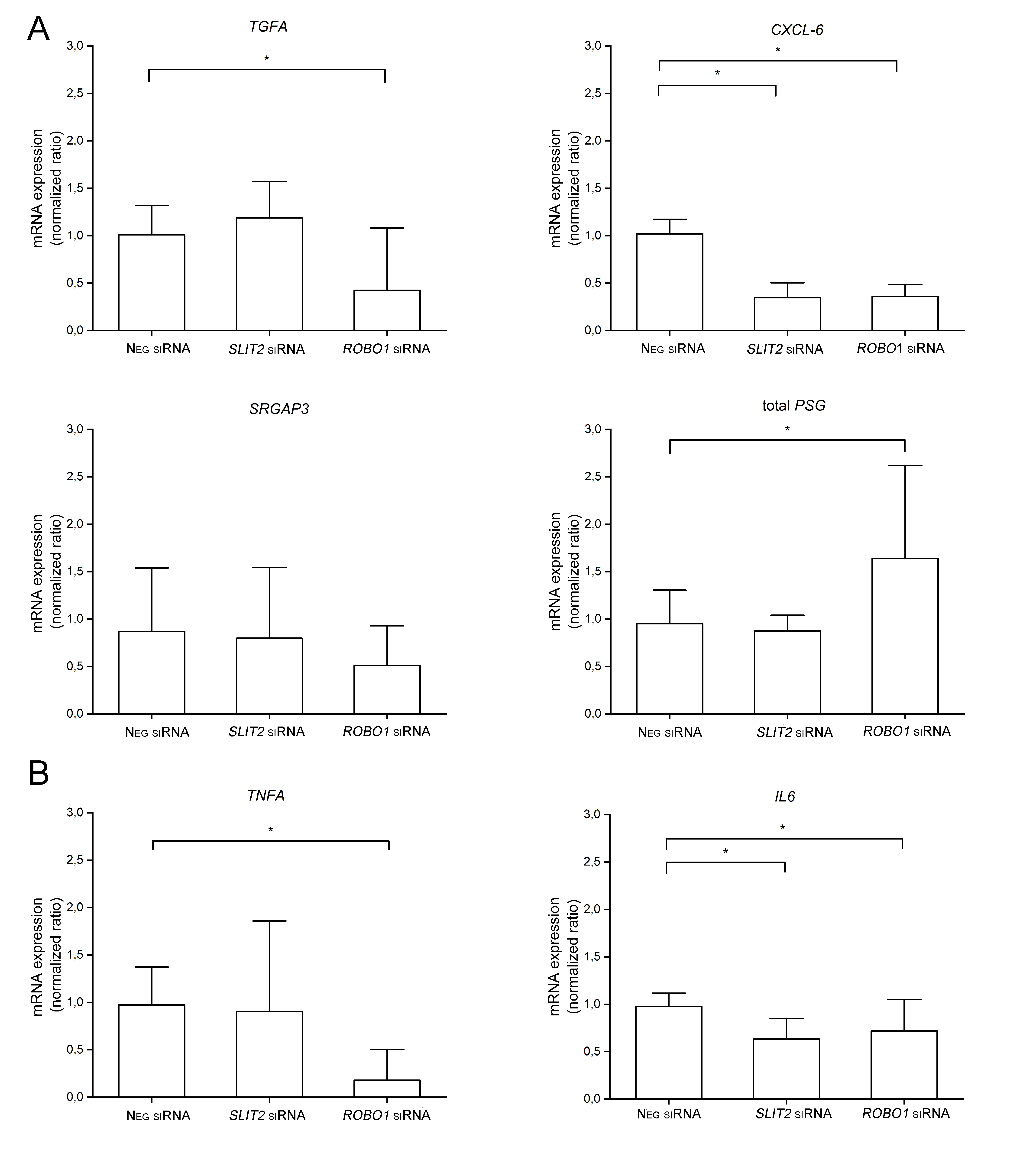

Supplement: S4 Fig — (A) Four genes/gene families affected by ROBO1 silencing according to RNA sequencing were verified by qRT-PCR. (B) Expression changes of inflammation-associated cytokines IL-6 and TNF-A after SLIT2 and ROBO1 silencing were also measured with qRT-PCR. SLIT2 and ROBO1 were post-transcriptionally silenced in HTR8/SVneo cell line by siRNA. mRNA levels of selected genes compared between SLIT2- or ROBO1-silenced cells and mRNA levels of untreated control cells. All mRNA levels normalized against mRNA levels of housekeeping gene CYC1. Columns represent median and SD values of the sample groups. (TIF) [file pgen.1008107.s004.tif]
