# Supplementary material for: Risk of spontaneous preterm birth and fetal growth associates with fetal SLIT2
Source: PLoS Genet. 2019 Jun 13;15(6):e1008107. doi: 10.1371/journal.pgen.1008107 (PMC6563950; doi:10.1371/journal.pgen.1008107)
Supplement: S1 Table — (DOCX) [file pgen.1008107.s005.docx]

| **Characteristic** | **Discovery GWAS^a^** | |
| --- | --- | --- |
|  | **Cases** | **Controls** |
| *n* | 247 | 419 |
| Male / female*, n* | 131 / 116 | 216 / 203 |
| Single preterm delivery in family, *n* | 178^b^ |  |
| Multiple preterm deliveries in family, *n*  2 preterm deliveries, *n*  3-6 preterm deliveries, *n* | 32^b^  19  13 |  |
| Very preterm infants (GA 23-31 wk) | 172 |  |
| Moderate-to-late preterm infants (GA 32-36 wk) | 75 |  |
| Gestational age, weeks, mean (range) | 30.3  (24.6-36.1) | 40.1  (38.0-41.9) |
| Birthweight, g, mean (range) | 1,603  (538-3,274) | 3,690  (2,534-5,140) |
| PPROM^c^, yes / no, *n* | 133 / 101^d^ |  |

^a^Infants born after spontaneous onset of preterm (cases) and term (controls) delivery; sampled in Oulu and Tampere University Hospitals.

^b^Information about total number of preterm deliveries in family not available for 37 infants.

^c^PPROM defined as rupture of fetal membranes >1 h before onset of contractions.

^d^Definition of PPROM unclear for 13 cases.
