# Supplementary material for: Risk of spontaneous preterm birth and fetal growth associates with fetal SLIT2
Source: PLoS Genet. 2019 Jun 13;15(6):e1008107. doi: 10.1371/journal.pgen.1008107 (PMC6563950; doi:10.1371/journal.pgen.1008107)
Supplement: S2 Table — (DOCX) [file pgen.1008107.s006.docx]

| **Chr** | **Gene**^a^ | **SNP**^a^ | **Reference allele** | **Effect^b^** | ***p*** |
| --- | --- | --- | --- | --- | --- |
| 4 | *SLIT2* | rs116461311 | C | -3.75 | 3.15E-07 |
| 10 | *C10orf90* | rs35113467 | C | -1.88 | 5.11E-07 |
| 7 | *SDK1* | rs8180800 | C | -1.56 | 1.61E-06 |
| 7 | *SUGCT* | rs117058392 | A | -2.19 | 2.59E-06 |
| 6 | Intergenic (*LOC105377949, LOC107986634*) | rs34514373 | Del | -1.58 | 3.72E-06 |
| 2 | *ALK* | rs7608573 | T | 1.78 | 3.80E-06 |
| 17 | *ABCA9* | rs11077860 | T | 1.43 | 4.74E-06 |
| 3 | *SENP5* | rs4916578 | A | -3.15 | 6.88E-06 |
| 8 | *DLC1* | rs7006225 | A | 1.60 | 7.42E-06 |

^a^Top SNP shown for each region.

^b^Regression coefficient of reference allele for gestational age in weeks.
