# Supplementary material for: Risk of spontaneous preterm birth and fetal growth associates with fetal SLIT2
Source: PLoS Genet. 2019 Jun 13;15(6):e1008107. doi: 10.1371/journal.pgen.1008107 (PMC6563950; doi:10.1371/journal.pgen.1008107)
Supplement: S3 Table — (DOCX) [file pgen.1008107.s007.docx]

| **Chr** | **Gene**^a^ | **SNP**^b^ | **Reference allele** | **Odds ratio** | ***p*** |
| --- | --- | --- | --- | --- | --- |
| 4 | *SLIT2* | rs116461311 | C | 4.06 | 1.55E-07 |
| 10 | *C10orf90* | rs35113467 | C | 1.87 | 2.20E-07 |
| 20 | *STK35* | rs72545460 | Ins | 1.72 | 1.18E-06 |
| 1 | *COL8A2* | rs146427742 | Ins | 2.85 | 2.00E-06 |
| 11 | Intergenic (*CCKBR, CNGA4)* | rs10839537 | C | 2.18 | 2.11E-06 |
| 17 | *SLC39A11* | rs28677989 | C | 2.60 | 2.51E-06 |
| 10 | *EXOSC1-ZDHHC16-MMS19-UBTD12* | rs10678727 | Ins | 1.90 | 2.81E-06 |
| 2 | Intergenic *(THUMPD2, SLC8A1)* | rs17480505 | C | 1.82 | 3.45E-06 |
| 13 | Intergenic *(ARHGEF, LINC00431)* | rs11490380 | C | 2.16 | 3.74E-06 |
| 2 | *MEIS1* | rs138191925 | C | 3.25 | 4.51E-06 |
| 14 | Intergenic *(TBPL2, KTN1)* | rs2181743 | T | 0.46 | 5.04E-06 |
| 17 | Intergenic (*C17orf67, DGKE*) | rs73323006 | A | 3.19 | 8.23E-06 |
| 9 | *TOPORS* | rs112527210 | A | 2.57 | 8.64E-06 |

^a^Two nearest loci shown in parentheses for intergenic SNPs.

^b^Top SNP shown for each region.
