# Supplementary material for: Risk of spontaneous preterm birth and fetal growth associates with fetal SLIT2
Source: PLoS Genet. 2019 Jun 13;15(6):e1008107. doi: 10.1371/journal.pgen.1008107 (PMC6563950; doi:10.1371/journal.pgen.1008107)
Supplement: S4 Table — (DOCX) [file pgen.1008107.s008.docx]

| **Chr** | **Gene**^a^ | **SNP**^b^ | **Reference allele** | **Odds ratio** | ***p*** |
| --- | --- | --- | --- | --- | --- |
| 22 | *KIAA1644* | rs737263 | A | 2.12 | 9.23E-8 |
| 14 | Intergenic (*OTX2, EXOCS5)* | rs2146858 | A | 2.44 | 1.53E-7 |
| 9 | *LINC01239* | rs687184 | C | 0.28 | 1.99E-7 |
| 17 | *TBCD* | rs111873142 | T | 3.61 | 1.18E-6 |
| 3 | *VGLL4* | rs73814923 | A | 5.27 | 1.27E-6 |
| 5 | *CDKL3* | rs112349722 | C | 4.77 | 1.66E-6 |
| 2 | *MYT1L* | rs72767375 | T | 4.24 | 1.76E-6 |
| 3 | Intergenic (*LOC105377173, ROBO1*) | rs115723230 | T | 4.88 | 2.34E-6 |
| 1 | *NMNAT2* | rs79033343 | T | 3.00 | 3.01E-6 |
| 7 | *SDK1* | rs56309858 | T | 2.20 | 3.07E-6 |
| 6 | Intergenic *(LOC107986540)* | rs200390855 | T | 3.01 | 3.17E-6 |
| 3 | *KALRN* | rs75292075 | C | 3.61 | 3.96E-6 |
| 8 | Intergenic *(EXTL3, TNTS9)* | rs62504329 | A | 0.39 | 4.14E-6 |
| 18 | Intergenic *(LOC105372151, NFEL23P1)* | rs112843209 | Ins | 2.72 | 4.16E-6 |
| 5 | Intergenic *(EFNA5, FBXL17)* | rs72134357 | G | 2.23 | 4.20E-6 |
| 10 | *ADAMTS14* | rs7093347 | T | 0.29 | 4.27E-6 |
| 9 | Intergenic *(PTPRD ,LOC105375974)* | rs143057876 | T | 4.31 | 4.35E-6 |
| 9 | Intergenic (*LOC107987084, TLE1*) | rs192868041 | G | 4.44 | 4.36E-6 |
| 1 | *KAZN* | rs55889542 | A | 3.85 | 4.81E-6 |
| 19 | Intergenic (*LINC01532, UQCRSF1*) | rs17716275 | A | 5.09 | 5.02E-6 |
| 7 | *HDAC9* | rs2106507 | C | 2.81 | 6.17E-6 |
| 14 | *NPAS3* | rs4627235 | A | 2.32 | 6.35E-6 |
| 10 | *KIAA1217* | rs75495909 | T | 3.76 | 6.56E-6 |
| 9 | *SLC28A3* | rs10735568 | C | 0.46 | 7.31E-6 |

^a^Two nearest loci shown for intergenic SNPs.

^b^Top SNP shown for each region.
