# Supplementary material for: Risk of spontaneous preterm birth and fetal growth associates with fetal SLIT2
Source: PLoS Genet. 2019 Jun 13;15(6):e1008107. doi: 10.1371/journal.pgen.1008107 (PMC6563950; doi:10.1371/journal.pgen.1008107)
Supplement: S5 Table — (DOCX) [file pgen.1008107.s009.docx]

| **Chr** | **Gene**^a^ | **SNP**^b^ | **Reference allele** | **Odds ratio** | ***p*** |
| --- | --- | --- | --- | --- | --- |
| 8 | Intergenic *(LOC102467222, LOC286114*) | rs117023642 | G | 5.85 | 3.63E-7 |
| 9 | Intergenic *(LOC105376108, SPATA31D4)* | rs11788387 | T | 2.96 | 5.92E-7 |
| 10 | *EXOSC1-ZDHHC16-MMS19-UBTD12* | rs4919105 | G | 2.02 | 1.60E-6 |
| 3 | *SUCLG2* | rs201608472 | G | 2.15 | 1.99E-6 |
| 12 | *KSR2* | rs642846 | A | 1.94 | 2.13E-6 |
| 6 | *NKAIN2* | rs17766756 | G | 3.78 | 3.06E-6 |
| 3 | *VGLL4* | rs2616554 | G | 1.95 | 3.89E-6 |
| 2 | Intergenic *(GALNT3, LOC105373730)* | rs34288836 | A | 2.52 | 4.03E-6 |
| 4 | Intergenic *(FSTL5, LOC101928052)* | rs202013048 | G | 3.78 | 4.20E-6 |
| 1 | Intergenic *(LOC105378769, LINC00466)* | rs7537459 | C | 0.37 | 4.59E-6 |
| 10 | *ADAMTS14* | rs12765664 | A | 0.34 | 4.66E-6 |
| 11 | Intergenic *(BARX2, TMEM45B)* | rs6590390 | A | 2.13 | 4.97E-6 |
| 18 | *FHOD3* | rs7231406 | A | 2.30 | 6.36E-6 |
| 13 | *FNDC3A* | rs118112762 | G | 3.25 | 6.57E-6 |
| 11 | *LGR4* | rs61887835 | C | 4.70 | 6.80E-6 |
| 14 | Intergenic *(LOC728755, LINC00645)* | rs2807820 | A | 0.51 | 7.23E-6 |
| 5 | Intergenic *(LOC105377677, LOC107986466)* | rs4704916 | A | 2.13 | 7.97E-6 |
| 17 | *MSI1* | rs113018921 | G | 2.82 | 8.15E-6 |

^a^Two nearest loci shown for intergenic SNPs.

^b^Top SNP shown for each region.
