# Supplementary material for: Risk of spontaneous preterm birth and fetal growth associates with fetal SLIT2
Source: PLoS Genet. 2019 Jun 13;15(6):e1008107. doi: 10.1371/journal.pgen.1008107 (PMC6563950; doi:10.1371/journal.pgen.1008107)
Supplement: S6 Table — (DOCX) [file pgen.1008107.s010.docx]

| **Chr** | **Gene**^a^ | **SNP**^b^ | **Reference allele** | **Odds ratio** | ***p*** |
| --- | --- | --- | --- | --- | --- |
| 3 | Intergenic (*LOC105377114, PTPRG*) | rs2001841 | A | 0.40 | 6.03E-7 |
| 3 | *LOC101928583* | rs34635471 | Del | 2.56 | 9.35E-7 |
| 18 | *TMEM241-RIOK3* | rs140162370 | C | 3.38 | 1.02E-6 |
| 13 | Intergenic (*LOC105370150, RXFP2*) | rs61953951 | A | 1.89 | 1.20E-6 |
| 1 | *EIF1P3* | rs539974331 | Del | 4.82 | 1.27E-6 |
| 18 | Intergenic (*LOC105372151, NFE2L3P1*) | rs10871774 | A | 0.38 | 1.71E-6 |
| 1 | Intergenic (*SLC7A14, LOC101926964*) | rs12085223 | G | 2.49 | 1.86E-6 |
| 21 | *DSCAM* | rs2410212 | T | 3.17 | 2.03E-6 |
| 6 | *NHSL1* | rs9389588 | A | 0.33 | 2.38E-6 |
| 12 | *NEUROD4* | rs188928429 | C | 3.78 | 2.78E-6 |
| 5 | *LVRN* | rs2115040 | A | 0.43 | 3.57E-6 |
| 10 | *GRID1* | rs572482355 | G | 3.78 | 3.77E-6 |
| 6 | Intergenic (*LOC101928842, HTR1E*) | rs9342062 | C | 2.85 | 4.05E-6 |
| 15 | *GABRB3* | rs112945849 | Ins | 2.42 | 5.12E-6 |
| 2 | Intergenic (*PDCL3, LOC105373507*) | rs68147667 | C | 2.78 | 5.36E-6 |
| 4 | Intergenic (*CENPE, TACR3*) | rs1289231 | C | 3.29 | 6.34E-6 |
| 10 | *STK32C* | rs1000535 | A | 2.22 | 6.63E-6 |
| 20 | *HSPA12B* | rs58505239 | T | 2.05 | 6.79E-6 |
| 17 | *TBX2* | rs59172073 | Del | 0.37 | 6.83E-6 |
| 9 | *SVEP1* | rs74514462 | A | 0.44 | 7.65E-6 |
| 13 | *KLHL1* | rs73212551 | G | 2.87 | 8.62E-6 |
| 4 | Intergenic (*LOC105377505, GUCY1A3*) | rs1965937 | A | 0.23 | 9.24E-6 |

^a^Two nearest loci shown for intergenic SNPs.

^b^Top SNP shown for each region.
