# Supplementary material for: Risk of spontaneous preterm birth and fetal growth associates with fetal SLIT2
Source: PLoS Genet. 2019 Jun 13;15(6):e1008107. doi: 10.1371/journal.pgen.1008107 (PMC6563950; doi:10.1371/journal.pgen.1008107)
Supplement: S7 Table — (DOCX) [file pgen.1008107.s011.docx]

| **Pathway** | **Pathway name** | ***p*** | **Corrected *p*^a^** | | **FDR** | | **Top genes in pathway**^b^ |
| --- | --- | --- | --- | --- | --- | --- | --- |
| hsa04360 | Axon guidance | 8.6E-10 | | 1.6E-07 | | 0 | ***SLIT2****,PAK6,NRP1,SEMA3E,ABLIM2* |
| hsa04510 | Focal adhesion | 6.6E-07 | | 6.2E-05 | | 0 | *PPP1CB,AKT3,PAK6,EGFR,PARVB* |
| hsa04270 | Vascular smooth muscle contraction | 1.4E-06 | | 8.9E-05 | | 0 | *PPP1CB,PLA2G4A,KCNMA1,ADRA1B,PRKG1* |
| hsa04720 | Long-term potentiation | 2.3E-05 | | 1.1E-03 | | 0 | *PPP1CB,GRIN2B,GRM5,CAMK2G,PLCB1* |
| hsa04730 | Long-term depression | 7.0E-05 | | 2.6E-03 | | 0 | *PLA2G4A,GRM5,PRKG1,LYN,PLCB1* |
| hsa04012 | ErbB signaling pathway | 4.1E-04 | | 0.01 | | 0 | *AKT3,PAK6,EGFR,PLCG1,NRG2* |
| hsa04514 | Cell adhesion molecules (CAMs) | 4.4E-04 | | 0.01 | | 0 | *HLA-DOA,NLGN1,CDH4,ITGAM,NCAM2* |
| hsa05412 | Arrhythmogenic right ventricular cardiomyopathy (ARVC) | 4.7E-04 | | 0.01 | | 0 | *CACNA2D3, CACNB2,CACNA1D,ACTN1* |
| hsa04512 | ECM-receptor interaction | 7.5E-04 | | 0.02 | | 0.13 | *LAMC3,COL4A1,LAMC2* |
| hsa05414 | Dilated cardiomyopathy | 1.4E-03 | | 0.03 | | 0.22 | *CACNA2D3, CACNB2,CACNA1D* |
| hsa04666 | Fc gamma R-mediated phagocytosis | 1.5E-03 | | 0.03 | | 0.20 | *AKT3,PLA2G4A,PLCG1,WASF1,LYN* |
| hsa04810 | Regulation of actin cytoskeleton | 1.6E-03 | | 0.03 | | 0.18 | *PPP1CB,RRAS,PAK6,EGFR,CYFIP1* |
| hsa04971 | Gastric acid secretion | 2.3E-03 | | 0.03 | | 0.17 | *CAMK2G,PLCB11,KCNQ1,SLC9A4,MYLK* |
| hsa04020 | Calcium signaling pathway | 2.8E-03 | | 0.04 | | 0.15 | *EGFR,PLCG1,ADRA1B,GRM5,ERBB4* |
| hsa05146 | Amoebiasis | 3.1E-03 | | 0.04 | | 0.14 | *ITGAM,IL1R2,LAMC3,PLCB1,ACTN1* |
| hsa05223 | Non-small cell lung cancer | 4.3E-03 | | 0.05 | | 0.13 | *AKT3,FHIT,EGFR,PLCG1* |

^a^KEGG pathways with corrected *p* < 0.05 shown.

^b^Top five genes or all genes with SNPs with *p* < 1E-02 in GWAS shown.
