# Supplementary material for: Risk of spontaneous preterm birth and fetal growth associates with fetal SLIT2
Source: PLoS Genet. 2019 Jun 13;15(6):e1008107. doi: 10.1371/journal.pgen.1008107 (PMC6563950; doi:10.1371/journal.pgen.1008107)
Supplement: S8 Table — (DOCX) [file pgen.1008107.s012.docx]

| **Gene ontology** | ***p*** | **Corrected *p*** | **FDR**^a^ | **Top genes^b^** |
| --- | --- | --- | --- | --- |
| synaptic membrane | 2.13E-09 | 9.25E-06 | 0 | *DLGAP1,FAIM2,GRM8,DLG2,GRIN2B* |
| dendritic spine | 4.60E-09 | 9.99E-06 | 0 | *DLGAP1,DLG2,GRIN2B,KCND2,NLGN1* |
| neuron spine | 4.60E-09 | 9.99E-06 | 0 | *DLGAP1,DLG2,GRIN2B,KCND2,NLGN1* |
| glutamate receptor activity | 6.41E-09 | 9.99E-06 | 0 | *GRM8,GRIN2B,GRM6,GRIK1,GRM5* |
| semaphorin-plexin signaling pathway | 2.31E-08 | 2.01E-05 | 0 | *NRP1,PLXNA4,SEMA3E,SEMA3A,NRP2* |
| voltage-gated cation channel activity | 2.39E-08 | 2.01E-05 | 0 | *CACNA2D3,KCND2,KCNMA1,KCNK1,GRIK1* |
| transmembrane receptor protein tyrosine kinase activity | 3.91E-08 | 2.43E-05 | 0 | *NRP1,CRIM1,EGFR,ROR2,ALK* |
| postsynaptic density | 9.59E-08 | 5.21E-05 | 0 | *DLGAP1,DLG2,GRIN2B,KCND2,NLGN1* |
| dendritic spine head | 9.59E-08 | 5.21E-05 | 0 | *DLGAP1,DLG2,GRIN2B,KCND2,NLGN1* |
| postsynaptic membrane | 1.25E-07 | 5.42E-05 | 0 | *DLGAP1,FAIM2,DLG2,GRIN2B,SYNE1* |
| voltage-gated ion channel activity | 2.07E-07 | 8.16E-05 | 0 | *CACNA2D3,KCND2,KCNMA1,KCNK1,GRIK1* |
| voltage-gated channel activity | 2.07E-07 | 8.16E-05 | 0 | *CACNA2D3,KCND2,KCNMA1,KCNK1,GRIK1* |
| actin filament binding | 3.68E-07 | 1.23E-04 | 0 | *EGFR,CORO1C,ANTXR1,CYFIP1,GAS7* |
| transmembrane receptor protein kinase activity | 4.09E-07 | 1.27E-04 | 0 | *NRP1,CRIM1,EGFR,ROR2,ALK* |
| extracellular matrix part | 4.80E-07 | 1.39E-04 | 0 | *ANG,GRIN2B,WDR33,ACAN,EMILIN2* |
| retinal ganglion cell axon guidance | 6.87E-07 | 1.87E-04 | 0 | ***SLIT2****,EFNA5,EPHB1,BMPR1B,ROBO2* |
| actin filament polymerization | 7.82E-07 | 2.00E-04 | 0 | *ANG,GAS7,WASF1,WASF3,JAK2* |
| telencephalon development | 1.32E-06 | 3.19E-04 | 0 | ***SLIT2****,NCOR2,BCBL11B,LMX1A,GRIN2B* |
| ion channel complex | 1.60E-06 | 3.67E-04 | 0 | *ANO4,KCBD2,KCNMA1,KCNK1,GABRB3* |
| neuron recognition | 2.45E-06 | 5.32E-04 | 0 | *SEMA3A,NTM,APP,CNTN4,EPHA3* |
| negative chemotaxis | 4.53E-06 | 9.37E-04 | 0 | ***SLIT2****,SEMA3A,NRP2,EPHA7,ROBO1* |
| cargo receptor activity | 8.05E-06 | 1.3E-03 | 0.04 | *CORIN,ENPP3,CD163L1,LRP6,HHIPL1* |
| learning | 7.84E-06 | 1.3E-03 | 0.04 | *GRIN2B,GRM6,GRIK1,GRM5,APP* |
| glutamate receptor signaling pathway | 7.50E-06 | 1.3E-03 | 0.04 | *GRIN2B,GRM6,GRIK1,GRM5,APP* |
| cation channel complex | 7.33E-06 | 1.3E-03 | 0.04 | *KCND2,KCNMA1,KCNK1,CACNB2,KCNT1* |
| membrane raft | 7.20E-06 | 1.3E-03 | 0.04 | *FAIM2,EGFR,KCNMA1,DPP4,NPC1* |
| basement membrane | 7.15E-06 | 1.3E-03 | 0.04 | *ANG,ACAN,COL15A1,LAMC3,COL4A1* |
| cell junction organization | 6.68E-06 | 1.3E-03 | 0.05 | *FERMT2,CDH6,MTDH,CDH4,CADM2* |
| heparin binding | 2.49E-05 | 2.5E-03 | 0.05 | ***SLIT2****,ANG,NRP1,F11,COL13A1* |
| voltage-gated potassium channel activity | 6.29E-06 | 1.2E-03 | 0.05 | *KCND2,KCNMA1,KCNK1,KCNT1* |
| protein tyrosine kinase activity | 2.44E-05 | 2.5E-03 | 0.05 | *NRP1,CRIM1,EGFR,TEC,ROR2* |
| regulation of phospholipase C activity | 1.02E-04 | 6.9E-03 | 0.05 | *ANG,EGFR,PLCG1,DLC1,PRKCE* |
| cadherin binding | 9.90E-05 | 6.9E-03 | 0.05 | *PTPRT,CDH13,TBC1D2,CTNNA2* |
| calcium-dependent phospholipid binding | 2.43E-05 | 2.5E-03 | 0.05 | *PLA2G4A,ANXA13,SYT6,ANXA6,DYSF* |
| Ras guanyl-nucleotide exchange factor activity | 9.36E-05 | 6.6E-03 | 0.05 | *FGD5,RASGEF1B,TIAM2,ARHGEF7,RLG1* |

^a^Gene ontologies with FDR < 0.05 shown.

^b^Top five genes linked to gene ontology shown.
