# Supplementary material for: Risk of spontaneous preterm birth and fetal growth associates with fetal SLIT2
Source: PLoS Genet. 2019 Jun 13;15(6):e1008107. doi: 10.1371/journal.pgen.1008107 (PMC6563950; doi:10.1371/journal.pgen.1008107)
Supplement: S9 Table — (DOCX) [file pgen.1008107.s013.docx]

| **Gene ontology**^a^ | ***SLIT2*** | ***ROBO1*** |
| --- | --- | --- |
| retinal ganglion cell axon guidance | X |  |
| telencephalon development | X | X |
| neuron recognition |  | X |
| negative chemotaxis | X | X |
| heparin binding | X |  |
| pattern binding | X |  |
| polysaccharide binding | X |  |
| glycosaminoglycan binding | X |  |
| regulation of neuron projection development | X | X |
| regulation of cell projection organization | X | X |
| positive regulation of cell development | X | X |
| forebrain development | X | X |
| central nervous system projection neuron axonogenesis | X |  |
| sprouting angiogenesis | X | X |
| axon part |  | X |
| regulation of dendrite development |  | X |
| cell recognition |  | X |
| pallium development | X | X |
| negative regulation of chemotaxis | X | X |
| positive regulation of cell projection organization | X | X |
| positive regulation of neurogenesis | X | X |
| regulation of endothelial cell migration | X |  |
| telencephalon cell migration | X | X |
| main axon |  | X |
| olfactory lobe development | X | X |
| regulation of cell morphogenesis involved in differentiation | X | X |
| olfactory bulb development | X | X |
| negative regulation of behavior | X | X |
| forebrain cell migration | X | X |

^a^Gene ontologies with corrected *p* < 0.05 including *SLIT2* and *ROBO1* genes shown. GOs shown in order of significance.
