# Supplementary material for: Risk of spontaneous preterm birth and fetal growth associates with fetal SLIT2
Source: PLoS Genet. 2019 Jun 13;15(6):e1008107. doi: 10.1371/journal.pgen.1008107 (PMC6563950; doi:10.1371/journal.pgen.1008107)
Supplement: S10 Table — SLIT2 silenced in HTR8/SVneo commercial cell line by siRNA. Transcriptome of these cells compared with transcriptome of cells treated with negative siRNA. Differentially expressed genes ranked based on FDR-adjusted p value and fold change (FC). Threshold of fold change was > 2.0, and threshold of FDR-adjusted p value was <0.05. (DOCX) [file pgen.1008107.s014.docx]

| Gene name | FC^a^ | p-value^b^ | adj. p-value^c^ | EntrezID | Description |
| --- | --- | --- | --- | --- | --- |
| *DNAH3* | 4.705 | 5.895E-06 | 0.000 | 55567 | dynein axonemal heavy chain 3 |
| *DCDC2* | 4.422 | 7.167E-05 | 0.000 | 51473 | doublecortin domain containing 2 |
| *VSTM1* | 3.701 | 1.011E-04 | 0.000 | 284415 | V-set and transmembrane domain containing 1 |
| *ITGB2* | 3.806 | 1.424E-04 | 0.000 | 3689 | integrin subunit beta 2 |
| *KRTAP2-3* | 3.329 | 1.483E-04 | 0.000 | 730755 | keratin associated protein 2-3 |
| *MIR4482* | 3.183 | 1.613E-04 | 0.000 | 100616323 | microRNA 4482 |
| *HES7* | 2.723 | 1.188E-04 | 0.000 | 84667 | hes family bHLH transcription factor 7 |
| *CLDN12* | 2.760 | 1.802E-05 | 0.000 | 9069 | claudin 12 |
| *TMEM64* | 2.310 | 1.306E-04 | 0.000 | 169200 | transmembrane protein 64 |
| *CHST11* | 2.314 | 1.967E-04 | 0.000 | 50515 | carbohydrate sulfotransferase 11 |
| *KLHL2* | 2.265 | 4.809E-05 | 0.000 | 11275 | kelch like family member 2 |
| *EIF4EBP2* | 2.138 | 8.936E-05 | 0.000 | 1979 | eukaryotic translation initiation factor 4E binding protein 2 |
| *IL15RA* | 2.063 | 1.908E-04 | 0.000 | 3601 | interleukin 15 receptor subunit alpha |
| *SETD7* | 2.038 | 7.757E-05 | 0.000 | 80854 | SET domain containing lysine methyltransferase 7 |

^a^Expression ratio (fold change) between compared sample groups. Comparison between *SLIT2-*silenced cells and negative control cells.

^b^*t*-test *p* value for comparisons between sample groups (*SLIT2*-silenced and negative-control cells).

^c^FDR-adjusted *p* value.
