# Supplementary material for: Risk of spontaneous preterm birth and fetal growth associates with fetal SLIT2
Source: PLoS Genet. 2019 Jun 13;15(6):e1008107. doi: 10.1371/journal.pgen.1008107 (PMC6563950; doi:10.1371/journal.pgen.1008107)
Supplement: S11 Table — SLIT2 silenced in HTR8/SVneo commercial cell line by siRNA. Transcriptome of these cells compared with transcriptome of cells treated with negative siRNA. Differentially expressed genes ranked based on FDR-adjusted p value and fold change. Threshold of fold change was > 2.0, and threshold of FDR-adjusted p value was <0.05. (DOCX) [file pgen.1008107.s015.docx]

| Gene name | FC^a^ | p-value^b^ | adj. p-value^c^ | EntrezID | Description |
| --- | --- | --- | --- | --- | --- |
| *NUCB1-AS1* | -6.413 | 2.391E-05 | 0.000 | 100874085 | NUCB1 antisense RNA 1 |
| *SCG2* | -4.375 | 3.630E-05 | 0.000 | 7857 | secretogranin II |
| *SLIT2* | -3.998 | 4.220E-05 | 0.000 | 9353 | slit guidance ligand 2 |
| *SPINK6* | -3.758 | 8.346E-05 | 0.000 | 404203 | serine peptidase inhibitor. Kazal type 6 |
| *KIAA1217* | -3.494 | 1.070E-04 | 0.000 | 56243 | KIAA1217 |
| *MATN3* | -3.159 | 5.399E-05 | 0.000 | 4148 | matrilin 3 |
| *BDKRB2* | -2.740 | 1.129E-04 | 0.000 | 624 | bradykinin receptor B2 |
| *LCTL* | -2.704 | 5.988E-05 | 0.000 | 197021 | lactase like |
| *MVP* | -2.445 | 1.247E-04 | 0.000 | 9961 | major vault protein |
| *CXCL1* | -2.314 | 9.525E-05 | 0.000 | 2919 | C-X-C motif chemokine ligand 1 |
| *GFPT2* | -2.290 | 1.790E-04 | 0.000 | 9945 | glutamine-fructose-6-phosphate transaminase 2 |
| *SFRP1* | -2.185 | 2.099E-04 | 0.000 | 6422 | secreted frizzled related protein 1 |

^a^Expression ratio (fold change) between compared sample groups. Comparison between *SLIT2-*silenced cells and negative-control cells.

^b^*t*-test *p* value for comparison between sample groups (*SLIT2*-silenced and negative-control cells).

^c^FDR-adjusted *p* value
