# Supplementary material for: Risk of spontaneous preterm birth and fetal growth associates with fetal SLIT2
Source: PLoS Genet. 2019 Jun 13;15(6):e1008107. doi: 10.1371/journal.pgen.1008107 (PMC6563950; doi:10.1371/journal.pgen.1008107)
Supplement: S12 Table — ROBO1 silenced in HTR8/SVneo cell line by siRNA. Transcriptome of these cells compared with transcriptome of cells treated with negative siRNA. Differentially expressed genes ranked based on FDR-adjusted p value and fold change. Threshold of fold change was > 2.0, and threshold of FDR-adjusted p value was <0.05. (DOCX) [file pgen.1008107.s016.docx]

| Gene name | FC^a^ | p-value^b^ | adj. p-value^c^ | EntrezID | Description |
| --- | --- | --- | --- | --- | --- |
| *LOC100289650* | 8.613 | 8.580E-04 | 0.000 | 100289650 | uncharacterized LOC100289650 |
| *PTCSC3* | 6.133 | 2.479E-04 | 0.000 | 100886964 | papillary thyroid carcinoma susceptibility candidate 3 (non-protein coding) |
| *SPX* | 5.869 | 7.991E-04 | 0.000 | 80763 | spexin hormone |
| *PSG1* | 5.054 | 8.403E-04 | 0.000 | 5669 | pregnancy specific beta-1-glycoprotein 1 |
| *NR4A2* | 4.225 | 4.660E-04 | 0.000 | 4929 | nuclear receptor subfamily 4 group A member 2 |
| *PLCE1-AS1* | 6.758 | 1.324E-03 | 0.000 | 100128054 | PLCE1 antisense RNA 1 |
| *SFTA1P* | 5.343 | 4.716E-05 | 0.000 | 207107 | surfactant associated 1. pseudogene |
| *TYRP1* | 3.871 | 9.523E-04 | 0.000 | 7306 | tyrosinase related protein 1 |
| *KCNU1* | 4.009 | 1.053E-03 | 0.000 | 157855 | potassium calcium-activated channel subfamily U member 1 |
| *HIST1H4L* | 5.706 | 7.074E-05 | 0.000 | 8368 | histone cluster 1 H4 family member l |
| *MMP3* | 9.532 | 2.656E-04 | 0.000 | 4314 | matrix metallopeptidase 3 |
| *SPANXN4* | 4.219 | 5.895E-05 | 0.000 | 441525 | SPANX family member N4 |
| *PLEKHG7* | 6.644 | 4.248E-04 | 0.000 | 440107 | pleckstrin homology and RhoGEF domain containing G7 |
| *ANKRD1* | 3.482 | 6.484E-05 | 0.000 | 27063 | ankyrin repeat domain 1 |
| *VSTM1* | 3.446 | 3.537E-05 | 0.000 | 284415 | V-set and transmembrane domain containing 1 |
| *PTPRR* | 3.376 | 3.835E-04 | 0.000 | 5801 | protein tyrosine phosphatase. receptor type R |
| *IL7R* | 3.394 | 1.356E-04 | 0.000 | 3575 | interleukin 7 receptor |
| *PMAIP1* | 3.126 | 1.179E-05 | 0.000 | 5366 | phorbol-12-myristate-13-acetate-induced protein 1 |
| *IL24* | 3.658 | 8.698E-04 | 0.000 | 11009 | interleukin 24 |
| *SCN3B* | 3.021 | 1.242E-03 | 0.000 | 55800 | sodium voltage-gated channel beta subunit 3 |
| *MYEOV* | 2.974 | 3.717E-04 | 0.000 | 26579 | myeloma overexpressed |
| *ADAMTS1* | 3.099 | 1.277E-03 | 0.000 | 9510 | ADAM metallopeptidase with thrombospondin type 1 motif 1 |
| *KRT34* | 3.453 | 2.774E-04 | 0.000 | 3885 | keratin 34 |
| *PPP1R15A* | 2.739 | 2.184E-04 | 0.000 | 23645 | protein phosphatase 1 regulatory subunit 15A |
| *ANXA2P3* | 2.949 | 1.295E-03 | 0.000 | 305 | annexin A2 pseudogene 3 |
| *VGF* | 2.816 | 6.205E-04 | 0.000 | 7425 | VGF nerve growth factor inducible |
| *WTAPP1* | 3.74 | 1.942E-03 | 0.003 | 100288077 | Wilms tumor 1 associated protein pseudogene 1 |
| *MMP1* | 2.923 | 3.010E-04 | 0.000 | 4312 | matrix metallopeptidase 1 |
| *SCG5* | 2.779 | 1.360E-03 | 0.000 | 6447 | secretogranin V |
| *IFRD1* | 2.671 | 2.361E-04 | 0.000 | 3475 | interferon related developmental regulator 1 |
| *C12orf73* | 2.669 | 3.894E-04 | 0.000 | 728568 | chromosome 12 open reading frame 73 |
| *SGK1* | 2.61 | 8.285E-04 | 0.000 | 6446 | serum/glucocorticoid regulated kinase 1 |
| *RND3* | 2.711 | 6.926E-04 | 0.000 | 390 | Rho family GTPase 3 |
| *CD163L1* | 2.598 | 4.071E-04 | 0.000 | 283316 | CD163 molecule like 1 |
| *ANXA2* | 2.442 | 1.772E-04 | 0.000 | 302 | annexin A2 |
| *LURAP1L-AS1* | 3.732 | 4.923E-03 | 0.004 | 101929467 | LURAP1L antisense RNA 1 |
| *ADAMTS5* | 2.739 | 1.212E-03 | 0.000 | 11096 | ADAM metallopeptidase with thrombospondin type 1 motif 5 |
| *PPME1* | 2.692 | 3.069E-04 | 0.000 | 51400 | protein phosphatase methylesterase 1 |
| *FST* | 2.603 | 1.301E-03 | 0.000 | 10468 | follistatin |
| *BEST3* | 3.079 | 3.452E-03 | 0.004 | 144453 | bestrophin 3 |
| *ADRB2* | 2.487 | 8.168E-04 | 0.000 | 154 | adrenoceptor beta 2 |
| *ANKRD30B* | 3.43 | 4.622E-03 | 0.004 | 374860 | ankyrin repeat domain 30B |
| *RGS7* | 2.507 | 1.179E-04 | 0.000 | 6000 | regulator of G-protein signaling 7 |
| *ANXA2P2* | 3.252 | 7.487E-03 | 0.004 | 304 | annexin A2 pseudogene 2 |
| *SH2D5* | 2.457 | 1.082E-03 | 0.000 | 400745 | SH2 domain containing 5 |
| *TFRC* | 2.296 | 1.595E-04 | 0.000 | 7037 | transferrin receptor |
| *FMN1* | 2.274 | 1.135E-03 | 0.000 | 342184 | formin 1 |
| *AADACP1* | 2.364 | 4.189E-04 | 0.000 | 201651 | arylacetamide deacetylase pseudogene 1 |
| *DAW1* | 2.562 | 1.788E-03 | 0.003 | 164781 | dynein assembly factor with WD repeats 1 |
| *TGFB2-AS1* | 2.819 | 9.823E-03 | 0.004 | 728463 | TGFB2 antisense RNA 1 (head to head) |
| *STXBP6* | 2.223 | 1.224E-03 | 0.000 | 29091 | syntaxin binding protein 6 |
| *ADM* | 2.243 | 8.050E-04 | 0.000 | 133 | adrenomedullin |
| *DDIT3* | 2.287 | 2.951E-04 | 0.000 | 1649 | DNA damage inducible transcript 3 |
| *ZNF542P* | 2.339 | 1.289E-03 | 0.000 | 147947 | zinc finger protein 542. pseudogene |
| *ARHGEF28* | 2.194 | 3.776E-04 | 0.000 | 64283 | Rho guanine nucleotide exchange factor 28 |
| *RELN* | 2.441 | 2.087E-03 | 0.003 | 5649 | reelin |
| *GBX2* | 2.181 | 6.330E-04 | 0.000 | 2637 | gastrulation brain homeobox 2 |
| *NLRP10* | 2.918 | 7.010E-03 | 0.004 | 338322 | NLR family pyrin domain containing 10 |
| *CARNMT1* | 2.349 | 1.741E-03 | 0.003 | 138199 | carnosine N-methyltransferase 1 |
| *IL12A* | 2.342 | 2.039E-03 | 0.003 | 3592 | interleukin 12A |
| *ODC1* | 2.217 | 6.808E-04 | 0.000 | 4953 | ornithine decarboxylase 1 |
| *TRMT61A* | 2.141 | 3.540E-04 | 0.000 | 115708 | tRNA methyltransferase 61A |
| *SAT1* | 2.19 | 1.165E-03 | 0.000 | 6303 | spermidine/spermine N1-acetyltransferase 1 |
| *CITED2* | 2.104 | 9.170E-04 | 0.000 | 10370 | Cbp/p300 interacting transactivator with Glu/Asp rich carboxy-terminal domain 2 |
| *OGFRL1* | 2.105 | 9.111E-04 | 0.000 | 79627 | opioid growth factor receptor like 1 |
| *NEDD9* | 2.119 | 4.542E-04 | 0.000 | 4739 | neural precursor cell expressed. developmentally down-regulated 9 |
| *YRDC* | 2.139 | 1.170E-03 | 0.000 | 79693 | yrdC N6-threonylcarbamoyltransferase domain containing |
| *KRTAP2-3* | 2.28 | 1.486E-03 | 0.003 | 730755 | keratin associated protein 2-3 |
| *SOX3* | 2.688 | 1.005E-02 | 0.004 | 6658 | SRY-box 3 |
| *ARHGAP18* | 2.154 | 7.162E-04 | 0.000 | 93663 | Rho GTPase activating protein 18 |
| *C6orf99* | 2.865 | 6.573E-03 | 0.004 | 100130967 | chromosome 6 open reading frame 99 |
| *SNAPC1* | 2.164 | 1.041E-03 | 0.000 | 6617 | small nuclear RNA activating complex polypeptide 1 |
| *SLC7A11-AS1* | 4.801 | 3.098E-03 | 0.004 | 641364 | SLC7A11 antisense RNA 1 |
| *MYL12B* | 2.208 | 1.468E-03 | 0.003 | 103910 | myosin light chain 12B |
| *COL8A1* | 2.085 | 9.464E-04 | 0.000 | 1295 | collagen type VIII alpha 1 chain |
| *EPHB1* | 3.086 | 3.416E-03 | 0.004 | 2047 | EPH receptor B1 |
| *FLRT2* | 2.39 | 4.675E-03 | 0.004 | 23768 | fibronectin leucine rich transmembrane protein 2 |
| *TFPI2* | 2.074 | 1.366E-03 | 0.000 | 7980 | tissue factor pathway inhibitor 2 |
| *RITA1* | 2.145 | 1.925E-03 | 0.003 | 84934 | RBPJ interacting and tubulin associated 1 |
| *VPS9D1-AS1* | 2.102 | 1.931E-03 | 0.003 | 100128881 | VPS9D1 antisense RNA 1 |
| *LINC01085* | 3.539 | 4.138E-03 | 0.004 | 152742 | long intergenic non-protein coding RNA 1085 |
| *MITF* | 2.046 | 7.457E-04 | 0.000 | 4286 | melanogenesis associated transcription factor |
| *EDN1* | 2.54 | 4.162E-03 | 0.004 | 1906 | endothelin 1 |
| *MPP4* | 2.144 | 1.948E-03 | 0.003 | 58538 | membrane palmitoylated protein 4 |
| *LINC01204* | 4.032 | 2.693E-03 | 0.004 | 101927528 | long intergenic non-protein coding RNA 1204 |
| *HBE1* | 17.451 | 4.782E-03 | 0.004 | 3046 | hemoglobin subunit epsilon 1 |
| *SEMA3E* | 4.479 | 6.383E-03 | 0.004 | 9723 | semaphorin 3E |
| *SNHG17* | 2.084 | 4.837E-04 | 0.000 | 388796 | small nucleolar RNA host gene 17 |
| *SLC7A11* | 2.245 | 2.472E-03 | 0.004 | 23657 | solute carrier family 7 member 11 |
| *CLDN12* | 2.003 | 5.144E-04 | 0.000 | 9069 | claudin 12 |
| *BTG4* | 3.301 | 5.426E-03 | 0.004 | 54766 | BTG anti-proliferation factor 4 |
| *LEAP2* | 2.313 | 8.097E-03 | 0.004 | 116842 | liver enriched antimicrobial peptide 2 |
| *PITRM1-AS1* | 2.295 | 3.711E-03 | 0.004 | 100507034 | PITRM1 antisense RNA 1 |
| *MIR181A2HG* | 3.388 | 6.019E-03 | 0.004 | 100379345 | MIR181A2 host gene |
| *MYPN* | 2.02 | 8.993E-04 | 0.000 | 84665 | myopalladin |
| *ZNF582* | 2.635 | 4.386E-03 | 0.004 | 147948 | zinc finger protein 582 |
| *HES7* | 2.379 | 7.867E-03 | 0.004 | 84667 | hes family bHLH transcription factor 7 |
| *PSG6* | 10.342 | 8.591E-03 | 0.004 | 5675 | pregnancy specific beta-1-glycoprotein 6 |
| *GAS5* | 2.157 | 4.740E-03 | 0.004 | 60674 | growth arrest specific 5 (non-protein coding) |
| *USP36* | 2.014 | 2.105E-03 | 0.003 | 57602 | ubiquitin specific peptidase 36 |
| *HRK* | 3.114 | 4.633E-03 | 0.004 | 8739 | harakiri. BCL2 interacting protein |
| *PHF21B* | 2.533 | 4.463E-03 | 0.004 | 112885 | PHD finger protein 21B |
| *MAPK13* | 2.053 | 4.404E-03 | 0.004 | 5603 | mitogen-activated protein kinase 13 |
| *REN* | 2.282 | 9.456E-03 | 0.004 | 5972 | renin |
| *PSG9* | 2.177 | 9.325E-03 | 0.004 | 5678 | pregnancy specific beta-1-glycoprotein 9 |
| *EBF2* | 3.636 | 2.813E-03 | 0.004 | 64641 | early B-cell factor 2 |
| *BACH1-IT2* | 3.49 | 7.375E-03 | 0.004 | 100874322 | BACH1 intronic transcript 2 |
| *CGA* | 2.516 | 3.523E-03 | 0.004 | 1081 | glycoprotein hormones. alpha polypeptide |
| *LOC100506178* | 3.166 | 7.631E-03 | 0.004 | 100506178 | uncharacterized LOC100506178 |
| *USP2-AS1* | 2.592 | 1.004E-02 | 0.004 | 100499227 | USP2 antisense RNA 1 (head to head) |
| *ADTRP* | 2.447 | 4.215E-03 | 0.004 | 84830 | androgen dependent TFPI regulating protein |
| *SGIP1* | 2.681 | 5.266E-03 | 0.004 | 84251 | SH3 domain GRB2 like endophilin interacting protein 1 |
| *AOC2* | 2.359 | 4.321E-03 | 0.004 | 314 | amine oxidase. copper containing 2 |
| *PSG4* | 2.258 | 2.449E-03 | 0.004 | 5672 | pregnancy specific beta-1-glycoprotein 4 |
| *SLIT2* | 2.046 | 4.882E-03 | 0.004 | 9353 | slit guidance ligand 2 |
| *PSG2* | 5.895 | 1.056E-02 | 0.005 | 5670 | pregnancy specific beta-1-glycoprotein 2 |
| *KCND3* | 2.932 | 3.676E-03 | 0.004 | 3752 | potassium voltage-gated channel subfamily D member 3 |
| *PIK3R6* | 3.17 | 6.389E-03 | 0.004 | 146850 | phosphoinositide-3-kinase regulatory subunit 6 |
| *MYCT1* | 7.56 | 1.101E-02 | 0.005 | 80177 | myc target 1 |
| *KCNH1* | 2.108 | 5.877E-03 | 0.004 | 3756 | potassium voltage-gated channel subfamily H member 1 |
| *TMCC1-AS1* | 2.309 | 5.325E-03 | 0.004 | 100507032 | TMCC1 antisense RNA 1 (head to head) |
| *CPLX1* | 2.207 | 9.211E-03 | 0.004 | 10815 | complexin 1 |
| *SNORD121A* | 4.541 | 1.146E-02 | 0.005 | 100113379 | small nucleolar RNA. C/D box 121A |
| *LOC100507065* | 2.896 | 3.605E-03 | 0.004 | 100507065 | uncharacterized LOC100507065 |
| *SP140* | 2.788 | 4.864E-03 | 0.004 | 11262 | SP140 nuclear body protein |
| *SIK1* | 2.354 | 2.266E-03 | 0.004 | 150094 | salt inducible kinase 1 |
| *SMPD3* | 2.234 | 3.340E-03 | 0.004 | 55512 | sphingomyelin phosphodiesterase 3 |
| *SPP1* | 2.514 | 8.526E-03 | 0.004 | 6696 | secreted phosphoprotein 1 |
| *LOC100507639* | 3.082 | 1.140E-02 | 0.005 | 100507639 | uncharacterized LOC100507639 |
| *SLC6A15* | 2.207 | 5.520E-03 | 0.004 | 55117 | solute carrier family 6 member 15 |
| *CD177* | 3.095 | 1.155E-02 | 0.005 | 57126 | CD177 molecule |
| *HRAT17* | 2.615 | 1.017E-02 | 0.005 | 101928036 | heart tissue-associated transcript 17 |
| *EMCN* | 5.433 | 1.225E-02 | 0.005 | 51705 | endomucin |
| *CRHBP* | 2.704 | 1.107E-02 | 0.005 | 1393 | corticotropin releasing hormone binding protein |
| *RSC1A1* | 2.072 | 2.581E-03 | 0.004 | 6248 | regulatory solute carrier protein. family 1. member 1 |
| *KCNK2* | 2.183 | 7.251E-03 | 0.004 | 3776 | potassium two pore domain channel subfamily K member 2 |
| *LINC01186* | 2.219 | 7.240E-03 | 0.004 | 101927574 | long intergenic non-protein coding RNA 1186 |
| *TRPC6* | 3.169 | 1.175E-02 | 0.005 | 7225 | transient receptor potential cation channel subfamily C member 6 |
| *KITLG* | 2.085 | 3.552E-03 | 0.004 | 4254 | KIT ligand |
| *FOXG1-AS1* | 4.579 | 1.288E-02 | 0.005 | 103695363 | FOXG1 antisense RNA 1 (head to head) |
| *ZNF781* | 2.806 | 1.160E-02 | 0.005 | 163115 | zinc finger protein 781 |
| *KBTBD8* | 2.252 | 9.509E-03 | 0.004 | 84541 | kelch repeat and BTB domain containing 8 |
| *SLC16A1-AS1* | 2.168 | 6.114E-03 | 0.004 | 100506392 | SLC16A1 antisense RNA 1 |
| *IGF2BP2-AS1* | 2.56 | 1.130E-02 | 0.005 | 646600 | IGF2BP2 antisense RNA 1 |
| *NGF* | 2.07 | 3.298E-03 | 0.004 | 4803 | nerve growth factor |
| *NABP1* | 2.012 | 4.339E-03 | 0.004 | 64859 | nucleic acid binding protein 1 |
| *VGLL3* | 2.11 | 2.295E-03 | 0.004 | 389136 | vestigial like family member 3 |
| *LOC284344* | 6.58 | 1.431E-02 | 0.006 | 284344 | uncharacterized LOC284344 |
| *PSG7* | 2.648 | 1.196E-02 | 0.005 | 5676 | pregnancy specific beta-1-glycoprotein 7 (gene/pseudogene) |
| *PRR9* | 3.147 | 1.374E-02 | 0.005 | 574414 | proline rich 9 |
| *LOC101928304* | 4.789 | 1.459E-02 | 0.006 | 101928304 | uncharacterized LOC101928304 |
| *ZNF699* | 2.075 | 5.437E-03 | 0.004 | 374879 | zinc finger protein 699 |
| *SH3BGRL2* | 2.013 | 3.965E-03 | 0.004 | 83699 | SH3 domain binding glutamate rich protein like 2 |
| *DNAJB13* | 4.347 | 1.587E-02 | 0.006 | 374407 | DnaJ heat shock protein family (Hsp40) member B13 |
| *CFP* | 2.732 | 1.310E-02 | 0.005 | 5199 | complement factor properdin |
| *SNHG1* | 2.06 | 2.278E-03 | 0.004 | 23642 | small nucleolar RNA host gene 1 |
| *MIR589* | 2.099 | 7.832E-03 | 0.004 | 693174 | microRNA 589 |
| *HIST1H4H* | 2.543 | 1.301E-02 | 0.005 | 8365 | histone cluster 1 H4 family member h |
| *AGTR1* | 2.038 | 5.414E-03 | 0.004 | 185 | angiotensin II receptor type 1 |
| *TEX15* | 2.095 | 1.128E-02 | 0.005 | 56154 | testis expressed 15 |
| *IL13RA2* | 2.05 | 4.433E-03 | 0.004 | 3598 | interleukin 13 receptor subunit alpha 2 |
| *ATP8A1* | 2.141 | 1.155E-02 | 0.005 | 10396 | ATPase phospholipid transporting 8A1 |
| *KRTAP4-7* | 8.444 | 1.625E-02 | 0.006 | 100132476 | keratin associated protein 4-7 |
| *SRRM5* | 2.288 | 1.250E-02 | 0.005 | 100170229 | serine/arginine repetitive matrix 5 |
| *MIR7111* | 2.274 | 1.371E-02 | 0.005 | 102465668 | microRNA 7111 |
| *SLC3A1* | 2.619 | 1.488E-02 | 0.006 | 6519 | solute carrier family 3 member 1 |
| *AOC3* | 2.452 | 1.451E-02 | 0.006 | 8639 | amine oxidase. copper containing 3 |
| *KRTAP1-5* | 2.457 | 1.465E-02 | 0.006 | 83895 | keratin associated protein 1-5 |
| *RNVU1-14* | 7.92 | 1.849E-02 | 0.006 | 101954266 | RNA. variant U1 small nuclear 14 |
| *IDI2-AS1* | 2.913 | 1.782E-02 | 0.006 | 55853 | IDI2 antisense RNA 1 |
| *LINC00702* | 2.364 | 1.528E-02 | 0.006 | 100652988 | long intergenic non-protein coding RNA 702 |
| *LOC100133091* | 2.028 | 1.281E-02 | 0.005 | 100133091 | uncharacterized LOC100133091 |
| *SLC1A3* | 2.061 | 1.261E-02 | 0.005 | 6507 | solute carrier family 1 member 3 |
| *PADI1* | 2.243 | 1.444E-02 | 0.006 | 29943 | peptidyl arginine deiminase 1 |
| *BMP6* | 2.037 | 1.246E-02 | 0.005 | 654 | bone morphogenetic protein 6 |
| *SLC28A3* | 2.021 | 1.279E-02 | 0.005 | 64078 | solute carrier family 28 member 3 |
| *LOC440028* | 2.39 | 1.621E-02 | 0.006 | 440028 | uncharacterized LOC440028 |
| *SNORA55* | 2.945 | 1.914E-02 | 0.007 | 677834 | small nucleolar RNA. H/ACA box 55 |
| *CARD18* | 2.347 | 1.669E-02 | 0.006 | 59082 | caspase recruitment domain family member 18 |
| *DCDC2* | 3.126 | 1.981E-02 | 0.007 | 51473 | doublecortin domain containing 2 |
| *CELF6* | 2.513 | 1.856E-02 | 0.006 | 60677 | CUGBP. Elav-like family member 6 |
| *HIF1A-AS2* | 3.893 | 2.145E-02 | 0.007 | 100750247 | HIF1A antisense RNA 2 |
| *SNORA23* | 2.399 | 1.659E-02 | 0.006 | 677808 | small nucleolar RNA. H/ACA box 23 |
| *CPA2* | 2.243 | 1.806E-02 | 0.006 | 1358 | carboxypeptidase A2 |
| *RPPH1* | 4.696 | 2.135E-02 | 0.007 | 85495 | ribonuclease P RNA component H1 |
| *DIRC3* | 2.121 | 1.792E-02 | 0.006 | 729582 | disrupted in renal carcinoma 3 |
| *HIST1H1E* | 2.222 | 1.801E-02 | 0.006 | 3008 | histone cluster 1 H1 family member e |
| *KRTAP4-9* | 10.594 | 2.264E-02 | 0.007 | 100132386 | keratin associated protein 4-9 |
| *SNORD3A* | 4.426 | 2.263E-02 | 0.007 | 780851 | small nucleolar RNA. C/D box 3A |
| *GSG1* | 2.71 | 2.049E-02 | 0.007 | 83445 | germ cell associated 1 |
| *FOLR3* | 3.954 | 2.294E-02 | 0.007 | 2352 | folate receptor 3 |
| *SNORD9* | 4.813 | 2.329E-02 | 0.008 | 692053 | small nucleolar RNA. C/D box 9 |
| *CFLAR-AS1* | 2.573 | 2.214E-02 | 0.000 | 65072 | CFLAR antisense RNA 1 |
| *SLFNL1-AS1* | 2.828 | 2.212E-02 | 0.000 | 100507178 | SLFNL1 antisense RNA 1 |
| *KRTAP4-12* | 2.758 | 2.040E-02 | 0.000 | 83755 | keratin associated protein 4-12 |
| *LOC102724084* | 4.498 | 2.431E-02 | 0.000 | 102724084 | uncharacterized LOC102724084 |
| *EDN2* | 3.317 | 2.364E-02 | 0.000 | 1907 | endothelin 2 |
| *MIR17HG* | 2.827 | 2.306E-02 | 0.000 | 407975 | miR-17-92a-1 cluster host gene |
| *LOC101927780* | 2.19 | 2.151E-02 | 0.000 | 101927780 | uncharacterized LOC101927780 |
| *RNVU1-15* | 2.532 | 2.340E-02 | 0.000 | 101954267 | RNA. variant U1 small nuclear 15 |
| *CPA3* | 2.107 | 2.159E-02 | 0.000 | 1359 | carboxypeptidase A3 |
| *CASS4* | 2.093 | 2.207E-02 | 0.000 | 57091 | Cas scaffolding protein family member 4 |
| *CAPSL* | 2.502 | 2.396E-02 | 0.000 | 133690 | calcyphosine like |
| *SAMD3* | 2.007 | 1.981E-02 | 0.000 | 154075 | sterile alpha motif domain containing 3 |
| *SNORA5B* | 2.684 | 2.527E-02 | 0.000 | 677795 | small nucleolar RNA. H/ACA box 5B |
| *DIO3* | 2.537 | 2.457E-02 | 0.000 | 1735 | deiodinase. iodothyronine type III |
| *XIRP1* | 2.146 | 2.259E-02 | 0.000 | 165904 | xin actin binding repeat containing 1 |
| *LINC00452* | 2.098 | 2.124E-02 | 0.000 | 643365 | long intergenic non-protein coding RNA 452 |
| *GOLT1A* | 3.896 | 2.870E-02 | 0.000 | 127845 | golgi transport 1A |
| *LINC00882* | 2.088 | 2.301E-02 | 0.000 | 100302640 | long intergenic non-protein coding RNA 882 |
| *PGM5P2* | 2.573 | 2.683E-02 | 0.000 | 595135 | phosphoglucomutase 5 pseudogene 2 |
| *LOC101926963* | 2.021 | 2.391E-02 | 0.000 | 101926963 | uncharacterized LOC101926963 |
| *ARRDC3-AS1* | 2.174 | 2.618E-02 | 0.000 | 100129716 | ARRDC3 antisense RNA 1 |
| *ANKRD20A5P* | 2.302 | 2.821E-02 | 0.000 | 440482 | ankyrin repeat domain 20 family member A5. pseudogene |
| *MIR3176* | 2.217 | 2.899E-02 | 0.000 | 100423037 | microRNA 3176 |
| *MIR1204* | 2.001 | 2.729E-02 | 0.000 | 100302185 | microRNA 1204 |

^a^Expression ratio (fold change) between compared sample groups. Comparison between *ROBO1-*silenced cells and negative-control cells.

^b^*t*-test *p* value for comparison between sample groups (*ROBO1-*silenced and negative-control cells).

^c^FDR-adjusted *p* value.
