# Supplementary material for: Risk of spontaneous preterm birth and fetal growth associates with fetal SLIT2
Source: PLoS Genet. 2019 Jun 13;15(6):e1008107. doi: 10.1371/journal.pgen.1008107 (PMC6563950; doi:10.1371/journal.pgen.1008107)
Supplement: S13 Table — ROBO1 silenced in HTR8/SVneo cell line by siRNA. Transcriptome of these cells compared with transcriptome of cells treated with negative siRNA. Differentially expressed genes ranked based on FDR-adjusted p value and fold change. Threshold of fold change was > 2.0, and threshold of FDR-adjusted p value was <0.01. (DOCX) [file pgen.1008107.s017.docx]

| Gene  name | FC^a^ | p-value^b^ | adj. p-value^c^ | EntrezID | Description |
| --- | --- | --- | --- | --- | --- |
| *CFI* | -6.371 | 7.398E-04 | 0.000 | 3426 | complement factor I |
| *IFIT1* | -5.431 | 2.715E-04 | 0.000 | 3434 | interferon induced protein with tetratricopeptide repeats 1 |
| *C1QTNF6* | -4.826 | 1.120E-04 | 0.000 | 114904 | C1q and tumor necrosis factor related protein 6 |
| *KIF12* | -7.081 | 1.147E-03 | 0.000 | 113220 | kinesin family member 12 |
| *RAB7B* | -5.588 | 8.757E-04 | 0.000 | 338382 | RAB7B. member RAS oncogene family |
| *C3* | -6.002 | 4.483E-04 | 0.000 | 718 | complement C3 |
| *RARRES3* | -8.194 | 5.733E-04 | 0.000 | 5920 | retinoic acid receptor responder 3 |
| *TNFRSF9* | -16.029 | 1.768E-05 | 0.000 | 3604 | TNF receptor superfamily member 9 |
| *ANXA10* | -5.316 | 1.713E-04 | 0.000 | 11199 | annexin A10 |
| *LCTL* | -4.274 | 1.002E-04 | 0.000 | 197021 | lactase like |
| *BDKRB2* | -6.184 | 2.947E-05 | 0.000 | 624 | bradykinin receptor B2 |
| *MMP2* | -4.317 | 3.422E-04 | 0.000 | 4313 | matrix metallopeptidase 2 |
| *IFI44* | -5.530 | 1.238E-04 | 0.000 | 10561 | interferon induced protein 44 |
| *ALDH3B1* | -3.808 | 1.890E-04 | 0.000 | 221 | aldehyde dehydrogenase 3 family member B1 |
| *DHRS3* | -7.516 | 4.012E-04 | 0.000 | 9249 | dehydrogenase/reductase 3 |
| *ROBO1* | -3.834 | 1.949E-04 | 0.000 | 6091 | roundabout guidance receptor 1 |
| *BDKRB1* | -4.496 | 7.663E-05 | 0.000 | 623 | bradykinin receptor B1 |
| *PTGES* | -3.479 | 6.028E-04 | 0.000 | 9536 | prostaglandin E synthase |
| *C10orf10* | -3.696 | 5.305E-05 | 0.000 | 11067 | chromosome 10 open reading frame 10 |
| *MPZL2* | -3.607 | 2.892E-04 | 0.000 | 10205 | myelin protein zero like 2 |
| *VWA5A* | -4.327 | 1.005E-03 | 0.000 | 4013 | von Willebrand factor A domain containing 5A |
| *LCP1* | -4.343 | 9.432E-05 | 0.000 | 3936 | lymphocyte cytosolic protein 1 |
| *SH2D1B* | -5.535 | 7.752E-04 | 0.000 | 117157 | SH2 domain containing 1B |
| *NXT2* | -3.906 | 1.194E-03 | 0.000 | 55916 | nuclear transport factor 2 like export factor 2 |
| *TNFRSF8* | -3.163 | 5.380E-04 | 0.000 | 943 | TNF receptor superfamily member 8 |
| *RCAN2* | -26.869 | 2.117E-03 | 0.003 | 10231 | regulator of calcineurin 2 |
| *SPRN* | -3.246 | 1.230E-03 | 0.000 | 503542 | shadow of prion protein homolog (zebrafish) |
| *RAB26* | -4.350 | 2.420E-04 | 0.000 | 25837 | RAB26. member RAS oncogene family |
| *SH3BGRL* | -4.401 | 2.538E-04 | 0.000 | 6451 | SH3 domain binding glutamate rich protein like |
| *GALNT9* | -4.314 | 1.297E-04 | 0.000 | 50614 | polypeptide N-acetylgalactosaminyltransferase 9 |
| *CEMIP* | -3.861 | 5.792E-04 | 0.000 | 57214 | cell migration inducing hyaluronan binding protein |
| *ALDH1A3* | -4.291 | 2.358E-05 | 0.000 | 220 | aldehyde dehydrogenase 1 family member A3 |
| *KIT* | -3.472 | 3.128E-04 | 0.000 | 3815 | KIT proto-oncogene receptor tyrosine kinase |
| *MT1F* | -3.526 | 6.448E-04 | 0.000 | 4494 | metallothionein 1F |
| *TJP3* | -3.323 | 1.023E-03 | 0.000 | 27134 | tight junction protein 3 |
| *FAXDC2* | -6.270 | 1.598E-03 | 0.003 | 10826 | fatty acid hydroxylase domain containing 2 |
| *MMP9* | -3.238 | 1.153E-03 | 0.000 | 4318 | matrix metallopeptidase 9 |
| *ZDHHC20* | -3.792 | 1.061E-04 | 0.000 | 253832 | zinc finger DHHC-type containing 20 |
| *MAP2K6* | -3.535 | 1.348E-03 | 0.000 | 5608 | mitogen-activated protein kinase kinase 6 |
| *CD300C* | -6.129 | 1.966E-03 | 0.003 | 10871 | CD300c molecule |
| *NYNRIN* | -3.642 | 1.536E-04 | 0.000 | 57523 | NYN domain and retroviral integrase containing |
| *AQP11* | -4.466 | 1.883E-03 | 0.003 | 282679 | aquaporin 11 |
| *SLC16A4* | -3.717 | 5.895E-06 | 0.000 | 9122 | solute carrier family 16 member 4 |
| *IL34* | -6.167 | 1.996E-03 | 0.003 | 146433 | interleukin 34 |
| *ICA1* | -3.127 | 1.254E-03 | 0.000 | 3382 | islet cell autoantigen 1 |
| *LINC00324* | -3.300 | 1.123E-03 | 0.000 | 284029 | long intergenic non-protein coding RNA 324 |
| *TTLL1* | -3.167 | 5.910E-04 | 0.000 | 25809 | tubulin tyrosine ligase like 1 |
| *P2RX6* | -3.561 | 2.008E-04 | 0.000 | 9127 | purinergic receptor P2X 6 |
| *ERICH5* | -2.878 | 5.321E-04 | 0.000 | 203111 | glutamate rich 5 |
| *SEMA3B* | -3.507 | 3.599E-04 | 0.000 | 7869 | semaphorin 3B |
| *ANK1* | -2.996 | 3.246E-04 | 0.000 | 286 | ankyrin 1 |
| *OPRL1* | -3.501 | 8.109E-04 | 0.000 | 4987 | opioid related nociceptin receptor 1 |
| *PTPN22* | -3.871 | 1.420E-03 | 0.003 | 26191 | protein tyrosine phosphatase. non-receptor type 22 |
| *PCDH7* | -3.333 | 9.406E-04 | 0.000 | 5099 | protocadherin 7 |
| *CPXM1* | -2.802 | 1.319E-03 | 0.000 | 56265 | carboxypeptidase X. M14 family member 1 |
| *ITGA11* | -3.459 | 5.262E-04 | 0.000 | 22801 | integrin subunit alpha 11 |
| *SERPINF1* | -4.736 | 1.462E-03 | 0.003 | 5176 | serpin family F member 1 |
| *NINJ2* | -4.767 | 1.563E-03 | 0.003 | 4815 | ninjurin 2 |
| *CXCL8* | -2.878 | 5.439E-04 | 0.000 | 3576 | C-X-C motif chemokine ligand 8 |
| *CCL2* | -4.748 | 1.841E-03 | 0.003 | 6347 | C-C motif chemokine ligand 2 |
| *UBA7* | -3.158 | 7.221E-04 | 0.000 | 7318 | ubiquitin like modifier activating enzyme 7 |
| *IL1R1* | -3.214 | 1.182E-03 | 0.000 | 3554 | interleukin 1 receptor type 1 |
| *CRISPLD1* | -3.120 | 6.389E-04 | 0.000 | 83690 | cysteine rich secretory protein LCCL domain containing 1 |
| *OLFML2B* | -4.017 | 1.907E-03 | 0.003 | 25903 | olfactomedin like 2B |
| *CYS1* | -4.036 | 1.681E-03 | 0.003 | 192668 | cystin 1 |
| *ACE* | -4.299 | 1.675E-03 | 0.003 | 1636 | angiotensin I converting enzyme |
| *ITGB4* | -3.126 | 1.076E-03 | 0.000 | 3691 | integrin subunit beta 4 |
| *CDH10* | -4.812 | 8.174E-03 | 0.004 | 1008 | cadherin 10 |
| *HIST1H2BK* | -3.081 | 2.067E-04 | 0.000 | 85236 | histone cluster 1 H2B family member k |
| *CD52* | -7.066 | 8.813E-03 | 0.004 | 1043 | CD52 molecule |
| *FCRLA* | -2.790 | 1.017E-03 | 0.000 | 84824 | Fc receptor like A |
| *ORAI3* | -3.213 | 6.146E-04 | 0.000 | 93129 | ORAI calcium release-activated calcium modulator 3 |
| *TRANK1* | -3.950 | 1.557E-03 | 0.003 | 9881 | tetratricopeptide repeat and ankyrin repeat containing 1 |
| *ID1* | -3.115 | 2.302E-04 | 0.000 | 3397 | inhibitor of DNA binding 1. HLH protein |
| *THBS2* | -2.682 | 7.516E-04 | 0.000 | 7058 | thrombospondin 2 |
| *LDB3* | -3.390 | 1.723E-03 | 0.003 | 11155 | LIM domain binding 3 |
| *RBM47* | -3.528 | 1.919E-03 | 0.003 | 54502 | RNA binding motif protein 47 |
| *IGFBP6* | -2.727 | 7.339E-04 | 0.000 | 3489 | insulin like growth factor binding protein 6 |
| *OLFML2A* | -3.639 | 1.633E-03 | 0.003 | 169611 | olfactomedin like 2A |
| *LTBP1* | -2.645 | 9.288E-04 | 0.000 | 4052 | latent transforming growth factor beta binding protein 1 |
| *CACNG6* | -2.614 | 1.207E-03 | 0.000 | 59285 | calcium voltage-gated channel auxiliary subunit gamma 6 |
| *HLA-DRB1* | -2.882 | 1.070E-03 | 0.000 | 3123 | major histocompatibility complex. class II. DR beta 1 |
| *TRIP13* | -3.025 | 1.831E-04 | 0.000 | 9319 | thyroid hormone receptor interactor 13 |
| *LOC100130093* | -3.011 | 7.634E-04 | 0.000 | NA | NA |
| *AIG1* | -2.600 | 3.953E-04 | 0.000 | 51390 | androgen induced 1 |
| *TMEM144* | -3.559 | 1.960E-03 | 0.003 | 55314 | transmembrane protein 144 |
| *RAG1* | -2.894 | 1.117E-03 | 0.000 | 5896 | recombination activating 1 |
| *GNG2* | -2.685 | 8.253E-05 | 0.000 | 54331 | G protein subunit gamma 2 |
| *SEPT4* | -2.982 | 1.824E-03 | 0.003 | 5414 | septin 4 |
| *PLLP* | -3.793 | 8.808E-03 | 0.004 | 51090 | plasmolipin |
| *TMX1* | -2.479 | 4.896E-04 | 0.000 | 81542 | thioredoxin related transmembrane protein 1 |
| *HAS3* | -2.739 | 4.126E-05 | 0.000 | 3038 | hyaluronan synthase 3 |
| *C1RL* | -2.817 | 1.159E-03 | 0.000 | 51279 | complement C1r subcomponent like |
| *AQP3* | -2.825 | 4.719E-04 | 0.000 | 360 | aquaporin 3 (Gill blood group) |
| *IL1A* | -2.488 | 5.498E-04 | 0.000 | 3552 | interleukin 1 alpha |
| *TUBA1A* | -2.559 | 1.236E-03 | 0.000 | 7846 | tubulin alpha 1a |
| *RHOJ* | -2.807 | 3.658E-04 | 0.000 | 57381 | ras homolog family member J |
| *ACTG2* | -3.304 | 7.534E-03 | 0.004 | 72 | actin. gamma 2. smooth muscle. enteric |
| *PCDHAC2* | -2.751 | 5.615E-04 | 0.000 | 56134 | protocadherin alpha subfamily C. 2 |
| *SUSD1* | -2.572 | 8.639E-04 | 0.000 | 64420 | sushi domain containing 1 |
| *CTSO* | -2.828 | 1.504E-03 | 0.003 | 1519 | cathepsin O |
| *OAS1* | -16.597 | 5.485E-03 | 0.004 | 4938 | 2'-5'-oligoadenylate synthetase 1 |
| *CEACAM1* | -6.279 | 5.112E-03 | 0.004 | 634 | carcinoembryonic antigen related cell adhesion molecule 1 |
| *LPAR5* | -3.652 | 9.634E-03 | 0.004 | 57121 | lysophosphatidic acid receptor 5 |
| *GATSL3* | -2.934 | 1.610E-03 | 0.003 | 652968 | GATS protein like 3 |
| *OLFML3* | -2.702 | 3.187E-04 | 0.000 | 56944 | olfactomedin like 3 |
| *SFRP1* | -2.606 | 6.867E-04 | 0.000 | 6422 | secreted frizzled related protein 1 |
| *FAM46C* | -2.388 | 1.271E-03 | 0.000 | 54855 | family with sequence similarity 46 member C |
| *MMP11* | -2.505 | 1.378E-03 | 0.000 | 4320 | matrix metallopeptidase 11 |
| *HGSNAT* | -2.543 | 1.654E-04 | 0.000 | 138050 | heparan-alpha-glucosaminide N-acetyltransferase |
| *NACAD* | -2.400 | 1.477E-04 | 0.000 | 23148 | NAC alpha domain containing |
| *ITGB3* | -2.418 | 9.936E-04 | 0.000 | 3690 | integrin subunit beta 3 |
| *WFDC21P* | -2.773 | 1.865E-03 | 0.003 | 645638 | WAP four-disulfide core domain 21. pseudogene |
| *ASPH* | -2.569 | 4.778E-04 | 0.000 | 444 | aspartate beta-hydroxylase |
| *COLEC12* | -2.658 | 1.806E-03 | 0.003 | 81035 | collectin subfamily member 12 |
| *GPR137C* | -2.667 | 1.651E-03 | 0.003 | 283554 | G protein-coupled receptor 137C |
| *CNTD1* | -3.359 | 3.935E-03 | 0.004 | 124817 | cyclin N-terminal domain containing 1 |
| *SLCO2A1* | -2.809 | 1.515E-03 | 0.003 | 6578 | solute carrier organic anion transporter family member 2A1 |
| *ADAMTSL4* | -2.418 | 1.047E-03 | 0.000 | 54507 | ADAMTS like 4 |
| *BCL3* | -2.381 | 3.363E-04 | 0.000 | 602 | B-cell CLL/lymphoma 3 |
| *C15orf48* | -4.004 | 3.062E-03 | 0.004 | 84419 | chromosome 15 open reading frame 48 |
| *ADAMTS2* | -2.304 | 7.044E-04 | 0.000 | 9509 | ADAM metallopeptidase with thrombospondin type 1 motif 2 |
| *ABAT* | -2.996 | 2.919E-03 | 0.004 | 18 | 4-aminobutyrate aminotransferase |
| *SSTR5* | -4.839 | 8.549E-03 | 0.004 | 6755 | somatostatin receptor 5 |
| *HSD3B7* | -2.774 | 1.706E-03 | 0.003 | 80270 | hydroxy-delta-5-steroid dehydrogenase. 3 beta- and steroid delta-isomerase 7 |
| *ODF3L2* | -4.556 | 6.286E-03 | 0.004 | 284451 | outer dense fiber of sperm tails 3 like 2 |
| *FAM20C* | -2.315 | 2.125E-04 | 0.000 | 56975 | FAM20C. golgi associated secretory pathway kinase |
| *DPY19L4* | -2.501 | 1.112E-03 | 0.000 | 286148 | dpy-19 like 4 (C. elegans) |
| *APCDD1L* | -2.464 | 1.058E-03 | 0.000 | 164284 | APC down-regulated 1 like |
| *OGDHL* | -2.408 | 2.243E-04 | 0.000 | 55753 | oxoglutarate dehydrogenase-like |
| *GALC* | -2.486 | 1.330E-03 | 0.000 | 2581 | galactosylceramidase |
| *ENTPD8* | -3.465 | 2.722E-03 | 0.004 | 377841 | ectonucleoside triphosphate diphosphohydrolase 8 |
| *LOC102724094* | -3.416 | 7.802E-03 | 0.004 | 102724094 | uncharacterized LOC102724094 |
| *PTP4A3* | -2.371 | 7.870E-04 | 0.000 | 11156 | protein tyrosine phosphatase type IVA. member 3 |
| *TLR3* | -2.535 | 1.445E-03 | 0.003 | 7098 | toll like receptor 3 |
| *SLC22A17* | -2.471 | 6.507E-04 | 0.000 | 51310 | solute carrier family 22 member 17 |
| *CRIP2* | -2.279 | 6.625E-04 | 0.000 | 1397 | cysteine rich protein 2 |
| *ACVRL1* | -3.571 | 9.110E-03 | 0.004 | 94 | activin A receptor like type 1 |
| *SCUBE2* | -2.501 | 9.995E-04 | 0.000 | 57758 | signal peptide. CUB domain and EGF like domain containing 2 |
| *KLHL22* | -2.354 | 8.842E-05 | 0.000 | 84861 | kelch like family member 22 |
| *MIR635* | -4.901 | 2.466E-03 | 0.004 | 693220 | microRNA 635 |
| *YPEL1* | -2.679 | 1.657E-03 | 0.003 | 29799 | yippee like 1 |
| *MAB21L2* | -2.447 | 8.344E-04 | 0.000 | 10586 | mab-21 like 2 |
| *PTGFRN* | -2.398 | 4.307E-04 | 0.000 | 5738 | prostaglandin F2 receptor inhibitor |
| *DENND6B* | -2.620 | 1.604E-03 | 0.003 | 414918 | DENN domain containing 6B |
| *PDE6G* | -2.633 | 1.408E-03 | 0.003 | 5148 | phosphodiesterase 6G |
| *SAMD14* | -2.492 | 2.008E-03 | 0.003 | 201191 | sterile alpha motif domain containing 14 |
| *FAM131B* | -2.485 | 2.033E-03 | 0.003 | 9715 | family with sequence similarity 131 member B |
| *LOC101927934* | -3.812 | 6.797E-03 | 0.004 | 101927934 | uncharacterized LOC101927934 |
| *FBXO2* | -2.492 | 1.396E-03 | 0.003 | 26232 | F-box protein 2 |
| *PCDHB14* | -2.530 | 1.645E-03 | 0.003 | 56122 | protocadherin beta 14 |
| *CXCL6* | -2.299 | 6.985E-04 | 0.000 | 6372 | C-X-C motif chemokine ligand 6 |
| *ADGRB2* | -2.436 | 1.510E-03 | 0.003 | 576 | adhesion G protein-coupled receptor B2 |
| *ACTA2* | -2.582 | 2.002E-03 | 0.003 | 59 | actin. alpha 2. smooth muscle. aorta |
| *IGLON5* | -4.516 | 7.572E-03 | 0.004 | 402665 | IgLON family member 5 |
| *PLA2R1* | -2.848 | 8.920E-03 | 0.004 | 22925 | phospholipase A2 receptor 1 |
| *ELF3* | -13.919 | 3.558E-03 | 0.004 | 1999 | E74 like ETS transcription factor 3 |
| *CPE* | -2.425 | 1.800E-03 | 0.003 | 1363 | carboxypeptidase E |
| *APOBEC3H* | -9.290 | 7.004E-03 | 0.004 | 164668 | apolipoprotein B mRNA editing enzyme catalytic subunit 3H |
| *SCN4B* | -2.805 | 4.351E-03 | 0.004 | 6330 | sodium voltage-gated channel beta subunit 4 |
| *CACNA2D2* | -2.449 | 1.574E-03 | 0.003 | 9254 | calcium voltage-gated channel auxiliary subunit alpha2delta 2 |
| *SMPD1* | -2.163 | 4.424E-04 | 0.000 | 6609 | sphingomyelin phosphodiesterase 1 |
| *GAPLINC* | -3.440 | 8.638E-03 | 0.004 | 100505592 | gastric adenocarcinoma associated. positive CD44 regulator. long intergenic non-coding RNA |
| *ZCCHC24* | -2.744 | 4.327E-03 | 0.004 | 219654 | zinc finger CCHC-type containing 24 |
| *SNCG* | -2.222 | 9.641E-04 | 0.000 | 6623 | synuclein gamma |
| *ANPEP* | -2.304 | 1.188E-03 | 0.000 | 290 | alanyl aminopeptidase. membrane |
| *TMEM173* | -2.165 | 3.481E-04 | 0.000 | 340061 | transmembrane protein 173 |
| *CYP1B1* | -3.585 | 2.196E-03 | 0.004 | 1545 | cytochrome P450 family 1 subfamily B member 1 |
| *HAND1* | -2.634 | 3.976E-03 | 0.004 | 9421 | heart and neural crest derivatives expressed 1 |
| *ALDOC* | -2.352 | 1.717E-03 | 0.003 | 230 | aldolase. fructose-bisphosphate C |
| *SMIM14* | -2.397 | 1.972E-03 | 0.003 | 201895 | small integral membrane protein 14 |
| *PIANP* | -2.109 | 1.141E-03 | 0.000 | 196500 | PILR alpha associated neural protein |
| *TSPAN15* | -2.177 | 8.227E-04 | 0.000 | 23555 | tetraspanin 15 |
| *PBX1* | -2.690 | 2.991E-03 | 0.004 | 5087 | PBX homeobox 1 |
| *SULF2* | -2.268 | 1.694E-03 | 0.003 | 55959 | sulfatase 2 |
| *SAMD9L* | -4.376 | 4.303E-03 | 0.004 | 219285 | sterile alpha motif domain containing 9 like |
| *CLCN3* | -2.187 | 9.582E-04 | 0.000 | 1182 | chloride voltage-gated channel 3 |
| *ADAMTSL3* | -2.114 | 6.749E-04 | 0.000 | 57188 | ADAMTS like 3 |
| *LDHD* | -3.315 | 3.735E-03 | 0.004 | 197257 | lactate dehydrogenase D |
| *PROM2* | -2.127 | 8.934E-04 | 0.000 | 150696 | prominin 2 |
| *C1S* | -3.521 | 2.545E-03 | 0.004 | 716 | complement C1s |
| *SEMA4B* | -2.256 | 1.871E-03 | 0.003 | 10509 | semaphorin 4B |
| *ST3GAL5* | -2.109 | 9.818E-04 | 0.000 | 8869 | ST3 beta-galactoside alpha-2.3-sialyltransferase 5 |
| *STRA6* | -2.401 | 4.841E-03 | 0.004 | 64220 | stimulated by retinoic acid 6 |
| *THBD* | -2.338 | 1.527E-03 | 0.003 | 7056 | thrombomodulin |
| *SHISA2* | -2.192 | 1.088E-03 | 0.000 | 387914 | shisa family member 2 |
| *PSMG3-AS1* | -2.594 | 3.994E-03 | 0.004 | 114796 | PSMG3 antisense RNA 1 (head to head) |
| *CAPN14* | -2.928 | 3.145E-03 | 0.004 | 440854 | calpain 14 |
| *RGS9* | -2.080 | 9.700E-04 | 0.000 | 8787 | regulator of G-protein signaling 9 |
| *BST2* | -2.849 | 4.805E-03 | 0.004 | 684 | bone marrow stromal cell antigen 2 |
| *APP* | -2.185 | 9.052E-04 | 0.000 | 351 | amyloid beta precursor protein |
| *CA9* | -2.978 | 2.640E-03 | 0.004 | 768 | carbonic anhydrase 9 |
| *UBAC2-AS1* | -2.189 | 1.064E-03 | 0.000 | 100289373 | UBAC2 antisense RNA 1 |
| *CD82* | -2.278 | 2.129E-03 | 0.003 | 3732 | CD82 molecule |
| *SNX10* | -2.221 | 1.521E-03 | 0.003 | 29887 | sorting nexin 10 |
| *TMEM151A* | -2.195 | 5.203E-04 | 0.000 | 256472 | transmembrane protein 151A |
| *CLSTN3* | -2.096 | 1.011E-03 | 0.000 | 9746 | calsyntenin 3 |
| *MRC2* | -2.182 | 7.103E-04 | 0.000 | 9902 | mannose receptor C type 2 |
| *PARP10* | -2.495 | 2.319E-03 | 0.004 | 84875 | poly(ADP-ribose) polymerase family member 10 |
| *SARDH* | -2.333 | 3.900E-03 | 0.004 | 1757 | sarcosine dehydrogenase |
| *BTN3A3* | -2.536 | 6.856E-03 | 0.004 | 10384 | butyrophilin subfamily 3 member A3 |
| *PLCD4* | -2.558 | 5.278E-03 | 0.004 | 84812 | phospholipase C delta 4 |
| *RBKS* | -2.635 | 9.657E-03 | 0.004 | 64080 | ribokinase |
| *TLL2* | -2.320 | 9.182E-03 | 0.004 | 7093 | tolloid like 2 |
| *LOC401052* | -4.000 | 5.989E-03 | 0.004 | 401052 | uncharacterized LOC401052 |
| *PCDHB5* | -2.100 | 1.266E-03 | 0.000 | 26167 | protocadherin beta 5 |
| *CCL7* | -6.106 | 2.478E-03 | 0.004 | 6354 | C-C motif chemokine ligand 7 |
| *BACE1* | -2.171 | 3.304E-04 | 0.000 | 23621 | beta-secretase 1 |
| *TMOD1* | -2.415 | 3.352E-03 | 0.004 | 7111 | tropomodulin 1 |
| *ISM2* | -2.252 | 2.081E-03 | 0.003 | 145501 | isthmin 2 |
| *KATNAL2* | -2.291 | 4.203E-03 | 0.004 | 83473 | katanin catalytic subunit A1 like 2 |
| *ACP5* | -2.550 | 5.342E-03 | 0.004 | 54 | acid phosphatase 5. tartrate resistant |
| *MVP* | -2.143 | 8.875E-04 | 0.000 | 9961 | major vault protein |
| *ECHDC3* | -2.388 | 7.080E-03 | 0.004 | 79746 | enoyl-CoA hydratase domain containing 3 |
| *MFI2* | -2.131 | 4.366E-04 | 0.000 | NA | NA |
| *ITGAX* | -4.503 | 3.694E-03 | 0.004 | 3687 | integrin subunit alpha X |
| *FGFR3* | -2.165 | 1.936E-03 | 0.003 | 2261 | fibroblast growth factor receptor 3 |
| *PBXIP1* | -2.016 | 1.248E-03 | 0.000 | 57326 | PBX homeobox interacting protein 1 |
| *IFIT2* | -3.929 | 4.941E-03 | 0.004 | 3433 | interferon induced protein with tetratricopeptide repeats 2 |
| *HELZ2* | -2.250 | 8.080E-03 | 0.004 | 85441 | helicase with zinc finger 2 |
| *RAET1G* | -2.972 | 5.555E-03 | 0.004 | 353091 | retinoic acid early transcript 1G |
| *SLC2A10* | -2.403 | 4.362E-03 | 0.004 | 81031 | solute carrier family 2 member 10 |
| *ARHGEF6* | -2.253 | 7.613E-03 | 0.004 | 9459 | Rac/Cdc42 guanine nucleotide exchange factor 6 |
| *IGSF10* | -4.101 | 9.947E-03 | 0.004 | 285313 | immunoglobulin superfamily member 10 |
| *SLC35D2* | -2.154 | 1.771E-03 | 0.003 | 11046 | solute carrier family 35 member D2 |
| *GALNT12* | -2.555 | 2.202E-03 | 0.004 | 79695 | polypeptide N-acetylgalactosaminyltransferase 12 |
| *COL9A3* | -2.738 | 4.144E-03 | 0.004 | 1299 | collagen type IX alpha 3 chain |
| *RNF11* | -2.046 | 8.816E-04 | 0.000 | 26994 | ring finger protein 11 |
| *MXD3* | -2.145 | 1.663E-03 | 0.003 | 83463 | MAX dimerization protein 3 |
| *ALDH1L1* | -2.520 | 2.675E-03 | 0.004 | 10840 | aldehyde dehydrogenase 1 family member L1 |
| *CLDN4* | -2.669 | 7.015E-03 | 0.004 | 1364 | claudin 4 |
| *SLC7A8* | -2.383 | 4.545E-03 | 0.004 | 23428 | solute carrier family 7 member 8 |
| *IL1B* | -2.091 | 9.877E-04 | 0.000 | 3553 | interleukin 1 beta |
| *DDO* | -3.183 | 2.460E-03 | 0.004 | 8528 | D-aspartate oxidase |
| *HAPLN3* | -2.429 | 2.425E-03 | 0.004 | 145864 | hyaluronan and proteoglycan link protein 3 |
| *NCSTN* | -2.022 | 6.087E-04 | 0.000 | 23385 | nicastrin |
| *TMEM150C* | -2.158 | 2.014E-03 | 0.003 | 441027 | transmembrane protein 150C |
| *CACNB3* | -2.038 | 7.280E-04 | 0.000 | 784 | calcium voltage-gated channel auxiliary subunit beta 3 |
| *SLC37A2* | -2.135 | 1.782E-03 | 0.003 | 219855 | solute carrier family 37 member 2 |
| *SLC22A18* | -2.782 | 2.604E-03 | 0.004 | 5002 | solute carrier family 22 member 18 |
| *LXN* | -2.543 | 7.808E-03 | 0.004 | 56925 | latexin |
| *RBM43* | -2.434 | 2.307E-03 | 0.004 | 375287 | RNA binding motif protein 43 |
| *SMAD6* | -2.774 | 2.407E-03 | 0.004 | 4091 | SMAD family member 6 |
| *PIGZ* | -6.632 | 4.723E-03 | 0.004 | 80235 | phosphatidylinositol glycan anchor biosynthesis class Z |
| *PTK2B* | -2.474 | 6.226E-03 | 0.004 | 2185 | protein tyrosine kinase 2 beta |
| *TMEM102* | -2.299 | 6.208E-03 | 0.004 | 284114 | transmembrane protein 102 |
| *BID* | -2.335 | 3.847E-03 | 0.004 | 637 | BH3 interacting domain death agonist |
| *PLAU* | -2.002 | 1.094E-03 | 0.000 | 5328 | plasminogen activator. urokinase |
| *PXMP4* | -2.075 | 1.639E-03 | 0.003 | 11264 | peroxisomal membrane protein 4 |
| *XKR9* | -4.366 | 6.196E-03 | 0.004 | 389668 | XK related 9 |
| *CXCL12* | -2.211 | 3.794E-03 | 0.004 | 6387 | C-X-C motif chemokine ligand 12 |
| *JUP* | -2.054 | 1.859E-03 | 0.003 | 3728 | junction plakoglobin |
| *ST6GALNAC2* | -10.941 | 3.180E-03 | 0.004 | 10610 | ST6 N-acetylgalactosaminide alpha-2.6-sialyltransferase 2 |
| *TXN2* | -2.118 | 3.358E-03 | 0.004 | 25828 | thioredoxin 2 |
| *TRPM4* | -2.131 | 2.354E-03 | 0.004 | 54795 | transient receptor potential cation channel subfamily M member 4 |
| *COL3A1* | -2.829 | 2.148E-03 | 0.004 | 1281 | collagen type III alpha 1 chain |
| *ZNF362* | -2.053 | 1.569E-03 | 0.003 | 149076 | zinc finger protein 362 |
| *NOTCH3* | -2.738 | 2.789E-03 | 0.004 | 4854 | notch 3 |
| *IL2RB* | -4.263 | 3.640E-03 | 0.004 | 3560 | interleukin 2 receptor subunit beta |
| *CRYL1* | -2.408 | 3.859E-03 | 0.004 | 51084 | crystallin lambda 1 |
| *TRPV4* | -2.010 | 1.336E-03 | 0.000 | 59341 | transient receptor potential cation channel subfamily V member 4 |
| *ABHD1* | -2.160 | 2.502E-03 | 0.004 | 84696 | abhydrolase domain containing 1 |
| *ZNF467* | -4.126 | 2.801E-03 | 0.004 | 168544 | zinc finger protein 467 |
| *CLDN5* | -5.933 | 5.147E-03 | 0.004 | 7122 | claudin 5 |
| *CD22* | -2.089 | 5.502E-03 | 0.004 | 933 | CD22 molecule |
| *SYCE2* | -2.301 | 5.812E-03 | 0.004 | 256126 | synaptonemal complex central element protein 2 |
| *SULT1A1* | -2.235 | 5.248E-03 | 0.004 | 6817 | sulfotransferase family 1A member 1 |
| *HTRA1* | -2.232 | 8.681E-03 | 0.004 | 5654 | HtrA serine peptidase 1 |
| *TINAGL1* | -2.103 | 6.083E-03 | 0.004 | 64129 | tubulointerstitial nephritis antigen like 1 |
| *SYNPO* | -2.001 | 1.622E-03 | 0.003 | 11346 | synaptopodin |
| *FANK1* | -2.697 | 2.907E-03 | 0.004 | 92565 | fibronectin type III and ankyrin repeat domains 1 |
| *OTOF* | -2.085 | 4.262E-03 | 0.004 | 9381 | otoferlin |
| *CACNG4* | -2.943 | 4.516E-03 | 0.004 | 27092 | calcium voltage-gated channel auxiliary subunit gamma 4 |
| *ADGRG1* | -2.013 | 1.402E-03 | 0.003 | 9289 | adhesion G protein-coupled receptor G1 |
| *PAQR8* | -2.439 | 4.297E-03 | 0.004 | 85315 | progestin and adipoQ receptor family member 8 |
| *PADI2* | -3.210 | 6.161E-03 | 0.004 | 11240 | peptidyl arginine deiminase 2 |
| *ITFG3* | -2.096 | 2.634E-03 | 0.004 | NA | NA |
| *ATOH8* | -4.051 | 3.499E-03 | 0.004 | 84913 | atonal bHLH transcription factor 8 |
| *TM7SF2* | -2.390 | 2.716E-03 | 0.004 | 7108 | transmembrane 7 superfamily member 2 |
| *SLITRK4* | -2.226 | 3.139E-03 | 0.004 | 139065 | SLIT and NTRK like family member 4 |
| *CLUL1* | -6.608 | 7.346E-03 | 0.004 | 27098 | clusterin like 1 |
| *PCSK9* | -4.040 | 9.098E-03 | 0.004 | 255738 | proprotein convertase subtilisin/kexin type 9 |
| *PLEKHG4B* | -2.539 | 4.150E-03 | 0.004 | 153478 | pleckstrin homology and RhoGEF domain containing G4B |
| *EPSTI1* | -2.359 | 5.313E-03 | 0.004 | 94240 | epithelial stromal interaction 1 (breast) |
| *GABRD* | -2.117 | 7.643E-03 | 0.004 | 2563 | gamma-aminobutyric acid type A receptor delta subunit |
| *H6PD* | -2.074 | 5.029E-03 | 0.004 | 9563 | hexose-6-phosphate dehydrogenase/glucose 1-dehydrogenase |
| *KIAA1683* | -3.490 | 8.168E-03 | 0.004 | 80726 | KIAA1683 |
| *SRPX* | -3.237 | 3.251E-03 | 0.004 | 8406 | sushi repeat containing protein. X-linked |
| *SLC40A1* | -2.594 | 4.421E-03 | 0.004 | 30061 | solute carrier family 40 member 1 |
| *SAMD9* | -2.755 | 2.783E-03 | 0.004 | 54809 | sterile alpha motif domain containing 9 |
| *KCNAB2* | -2.136 | 2.490E-03 | 0.004 | 8514 | potassium voltage-gated channel subfamily A regulatory beta subunit 2 |
| *CTBS* | -2.045 | 4.345E-03 | 0.004 | 1486 | chitobiase |
| *PCDH18* | -2.054 | 9.199E-03 | 0.004 | 54510 | protocadherin 18 |
| *EBI3* | -2.231 | 2.753E-03 | 0.004 | 10148 | Epstein-Barr virus induced 3 |
| *IGFBP5* | -2.588 | 2.431E-03 | 0.004 | 3488 | insulin like growth factor binding protein 5 |
| *ATP1B2* | -2.229 | 9.087E-03 | 0.004 | 482 | ATPase Na+/K+ transporting subunit beta 2 |
| *FBXL2* | -2.070 | 8.305E-03 | 0.004 | 25827 | F-box and leucine rich repeat protein 2 |
| *MCF2L* | -2.740 | 6.149E-03 | 0.004 | 23263 | MCF.2 cell line derived transforming sequence like |
| *IDH2* | -2.094 | 4.758E-03 | 0.004 | 3418 | isocitrate dehydrogenase (NADP(+)) 2. mitochondrial |
| *RIPK4* | -3.455 | 3.428E-03 | 0.004 | 54101 | receptor interacting serine/threonine kinase 4 |
| *SCNN1A* | -4.318 | 4.551E-03 | 0.004 | 6337 | sodium channel epithelial 1 alpha subunit |
| *GSN* | -2.172 | 2.166E-03 | 0.004 | 2934 | gelsolin |
| *HLA-DMA* | -2.496 | 2.272E-03 | 0.004 | 3108 | major histocompatibility complex. class II. DM alpha |
| *ZNF837* | -3.298 | 6.762E-03 | 0.004 | 116412 | zinc finger protein 837 |
| *ANKEF1* | -2.090 | 8.992E-03 | 0.004 | 63926 | ankyrin repeat and EF-hand domain containing 1 |
| *NOX5* | -2.774 | 4.227E-03 | 0.004 | 79400 | NADPH oxidase 5 |
| *MAT1A* | -4.214 | 4.888E-03 | 0.004 | 4143 | methionine adenosyltransferase 1A |
| *YPEL3* | -2.059 | 7.897E-03 | 0.004 | 83719 | yippee like 3 |
| *ISG15* | -2.182 | 3.286E-03 | 0.004 | 9636 | ISG15 ubiquitin-like modifier |
| *SRGAP3* | -2.187 | 3.481E-03 | 0.004 | 9901 | SLIT-ROBO Rho GTPase activating protein 3 |
| *BFSP1* | -2.199 | 5.818E-03 | 0.004 | 631 | beaded filament structural protein 1 |
| *BLACAT1* | -2.442 | 9.521E-03 | 0.004 | 101669762 | bladder cancer associated transcript 1 (non-protein coding) |
| *CD24* | -2.396 | 3.163E-03 | 0.004 | 100133941 | CD24 molecule |
| *MFAP4* | -2.217 | 7.210E-03 | 0.004 | 4239 | microfibrillar associated protein 4 |
| *KIAA1217* | -2.054 | 3.369E-03 | 0.004 | 56243 | KIAA1217 |
| *CTNNBIP1* | -2.063 | 2.669E-03 | 0.004 | 56998 | catenin beta interacting protein 1 |
| *CLIP3* | -2.037 | 2.185E-03 | 0.004 | 25999 | CAP-Gly domain containing linker protein 3 |
| *PTN* | -3.744 | 4.598E-03 | 0.004 | 5764 | pleiotrophin |
| *ADSSL1* | -2.287 | 7.637E-03 | 0.004 | 122622 | adenylosuccinate synthase like 1 |
| *CTF1* | -2.024 | 9.864E-03 | 0.004 | 1489 | cardiotrophin 1 |
| *CHST13* | -3.024 | 2.728E-03 | 0.004 | 166012 | carbohydrate sulfotransferase 13 |
| *HOXC4* | -2.015 | 1.009E-02 | 0.004 | 3221 | homeobox C4 |
| *THBS3* | -2.296 | 4.976E-03 | 0.004 | 7059 | thrombospondin 3 |
| *FMO5* | -3.004 | 6.738E-03 | 0.004 | 2330 | flavin containing monooxygenase 5 |
| *BTN3A2* | -2.264 | 4.097E-03 | 0.004 | 11118 | butyrophilin subfamily 3 member A2 |
| *WNT11* | -3.483 | 5.775E-03 | 0.004 | 7481 | Wnt family member 11 |
| *TMEM53* | -2.060 | 9.104E-03 | 0.004 | 79639 | transmembrane protein 53 |
| *RGS9BP* | -2.005 | 3.074E-03 | 0.004 | 388531 | regulator of G-protein signaling 9 binding protein |
| *EGF* | -4.088 | 5.420E-03 | 0.004 | 1950 | epidermal growth factor |
| *C20orf197* | -2.116 | 9.122E-03 | 0.004 | 284756 | chromosome 20 open reading frame 197 |
| *TMEM169* | -2.062 | 9.971E-03 | 0.004 | 92691 | transmembrane protein 169 |
| *OLFM1* | -2.453 | 9.491E-03 | 0.004 | 10439 | olfactomedin 1 |
| *PCDHGB7* | -2.088 | 6.691E-03 | 0.004 | 56099 | protocadherin gamma subfamily B. 7 |
| *DES* | -3.167 | 4.835E-03 | 0.004 | 1674 | desmin |
| *PEX11G* | -2.158 | 7.363E-03 | 0.004 | 92960 | peroxisomal biogenesis factor 11 gamma |
| *SNAP25* | -2.630 | 5.396E-03 | 0.004 | 6616 | synaptosome associated protein 25 |
| *CCDC88B* | -2.545 | 4.817E-03 | 0.004 | 283234 | coiled-coil domain containing 88B |
| *LOXL1* | -2.061 | 3.570E-03 | 0.004 | 4016 | lysyl oxidase like 1 |
| *FPR1* | -2.981 | 5.443E-03 | 0.004 | 2357 | formyl peptide receptor 1 |
| *ADCY5* | -2.171 | 6.377E-03 | 0.004 | 111 | adenylate cyclase 5 |
| *NPTX1* | -2.168 | 3.540E-03 | 0.004 | 4884 | neuronal pentraxin 1 |
| *CDC42BPG* | -2.669 | 8.322E-03 | 0.004 | 55561 | CDC42 binding protein kinase gamma |
| *BCKDHB* | -2.492 | 5.367E-03 | 0.004 | 594 | branched chain keto acid dehydrogenase E1 subunit beta |
| *RNASE4* | -2.128 | 4.055E-03 | 0.004 | 6038 | ribonuclease A family member 4 |
| *FXYD6* | -2.370 | 5.053E-03 | 0.004 | 53826 | FXYD domain containing ion transport regulator 6 |
| *PRKCDBP* | -2.125 | 2.598E-03 | 0.004 | 112464 | protein kinase C delta binding protein |
| *FBN1* | -2.093 | 5.632E-03 | 0.004 | 2200 | fibrillin 1 |
| *C1orf233* | -2.078 | 5.017E-03 | 0.004 | NA | NA |
| *ARHGAP9* | -2.921 | 5.047E-03 | 0.004 | 64333 | Rho GTPase activating protein 9 |
| *PAQR9* | -2.169 | 8.281E-03 | 0.004 | 344838 | progestin and adipoQ receptor family member 9 |
| *RARA-AS1* | -2.253 | 7.499E-03 | 0.004 | 101929693 | RARA antisense RNA 1 |
| *SERPINA5* | -5.678 | 1.124E-02 | 0.005 | 5104 | serpin family A member 5 |
| *TPPP3* | -2.030 | 6.406E-03 | 0.004 | 51673 | tubulin polymerization promoting protein family member 3 |
| *SYNC* | -2.084 | 3.039E-03 | 0.004 | 81493 | syncoilin. intermediate filament protein |
| *FBLN2* | -2.098 | 2.610E-03 | 0.004 | 2199 | fibulin 2 |
| *TMEM229B* | -2.378 | 3.853E-03 | 0.004 | 161145 | transmembrane protein 229B |
| *LOC100506476* | -4.122 | 1.100E-02 | 0.005 | 100506476 | uncharacterized LOC100506476 |
| *NOXA1* | -2.735 | 4.799E-03 | 0.004 | 10811 | NADPH oxidase activator 1 |
| *REEP6* | -2.052 | 3.800E-03 | 0.004 | 92840 | receptor accessory protein 6 |
| *MMP19* | -2.262 | 5.183E-03 | 0.004 | 4327 | matrix metallopeptidase 19 |
| *SHC4* | -2.016 | 7.122E-03 | 0.004 | 399694 | SHC adaptor protein 4 |
| *KNDC1* | -2.658 | 3.841E-03 | 0.004 | 85442 | kinase non-catalytic C-lobe domain containing 1 |
| *GNG7* | -2.028 | 7.908E-03 | 0.004 | 2788 | G protein subunit gamma 7 |
| *BCO2* | -2.912 | 4.292E-03 | 0.004 | 83875 | beta-carotene oxygenase 2 |
| *LOC100129550* | -2.079 | 5.289E-03 | 0.004 | 100129550 | uncharacterized LOC100129550 |
| *PRRT2* | -2.104 | 7.104E-03 | 0.004 | 112476 | proline rich transmembrane protein 2 |
| *ALDH6A1* | -2.057 | 4.280E-03 | 0.004 | 4329 | aldehyde dehydrogenase 6 family member A1 |
| *DLGAP3* | -2.262 | 8.328E-03 | 0.004 | 58512 | DLG associated protein 3 |
| *CTSH* | -2.038 | 7.926E-03 | 0.004 | 1512 | cathepsin H |
| *NFE2* | -3.624 | 1.047E-02 | 0.005 | 4778 | nuclear factor. erythroid 2 |
| *EFNA1* | -2.368 | 3.729E-03 | 0.004 | 1942 | ephrin A1 |
| *SYTL2* | -2.552 | 5.663E-03 | 0.004 | 54843 | synaptotagmin like 2 |
| *BMF* | -2.671 | 3.387E-03 | 0.004 | 90427 | Bcl2 modifying factor |
| *PCYOX1L* | -2.017 | 7.187E-03 | 0.004 | 78991 | prenylcysteine oxidase 1 like |
| *PRSS56* | -2.873 | 1.037E-02 | 0.005 | 646960 | protease. serine 56 |
| *LOC100129434* | -2.097 | 4.309E-03 | 0.004 | 100129434 | uncharacterized LOC100129434 |
| *FLVCR1-AS1* | -2.185 | 4.658E-03 | 0.004 | 642946 | FLVCR1 antisense RNA 1 (head to head) |
| *RUNX2* | -2.362 | 5.035E-03 | 0.004 | 860 | runt related transcription factor 2 |
| *GPAT3* | -2.098 | 2.496E-03 | 0.004 | 84803 | glycerol-3-phosphate acyltransferase 3 |
| *LAPTM5* | -2.811 | 2.747E-03 | 0.004 | 7805 | lysosomal protein transmembrane 5 |
| *GPX3* | -2.192 | 5.918E-03 | 0.004 | 2878 | glutathione peroxidase 3 |
| *NTSR1* | -2.101 | 6.214E-03 | 0.004 | 4923 | neurotensin receptor 1 |
| *CHKB-AS1* | -2.006 | 7.607E-03 | 0.004 | 100144603 | CHKB antisense RNA 1 (head to head) |
| *KCNF1* | -2.576 | 4.239E-03 | 0.004 | 3754 | potassium voltage-gated channel modifier subfamily F member 1 |
| *WDR63* | -2.223 | 9.817E-03 | 0.004 | 126820 | WD repeat domain 63 |
| *RASSF4* | -2.331 | 6.066E-03 | 0.004 | 83937 | Ras association domain family member 4 |
| *ZNF365* | -2.101 | 9.734E-03 | 0.004 | 22891 | zinc finger protein 365 |
| *TMEM63C* | -2.167 | 8.085E-03 | 0.004 | 57156 | transmembrane protein 63C |
| *TOB1-AS1* | -2.127 | 6.418E-03 | 0.004 | 400604 | TOB1 antisense RNA 1 |
| *PAX2* | -2.215 | 3.823E-03 | 0.004 | 5076 | paired box 2 |
| *SPEF1* | -2.157 | 3.133E-03 | 0.004 | 25876 | sperm flagellar 1 |
| *EFCAB12* | -2.694 | 7.590E-03 | 0.004 | 90288 | EF-hand calcium binding domain 12 |
| *LOC389247* | -3.400 | 1.115E-02 | 0.005 | 389247 | uncharacterized LOC389247 |
| *MAL* | -2.794 | 1.032E-02 | 0.005 | 4118 | mal. T-cell differentiation protein |
| *C1R* | -2.311 | 3.487E-03 | 0.004 | 715 | complement C1r |
| *BTN3A1* | -2.498 | 5.479E-03 | 0.004 | 11119 | butyrophilin subfamily 3 member A1 |
| *HSD11B2* | -2.861 | 1.082E-02 | 0.005 | 3291 | hydroxysteroid 11-beta dehydrogenase 2 |
| *BAIAP3* | -2.179 | 6.974E-03 | 0.004 | 8938 | BAI1 associated protein 3 |
| *SYNGR3* | -2.454 | 7.128E-03 | 0.004 | 9143 | synaptogyrin 3 |
| *ASB9* | -2.718 | 1.036E-02 | 0.005 | 140462 | ankyrin repeat and SOCS box containing 9 |
| *IQSEC2* | -2.102 | 6.054E-03 | 0.004 | 23096 | IQ motif and Sec7 domain 2 |
| *KLF9* | -2.036 | 3.929E-03 | 0.004 | 687 | Kruppel like factor 9 |
| *ARSI* | -2.107 | 6.614E-03 | 0.004 | 340075 | arylsulfatase family member I |
| *PLD1* | -2.117 | 4.451E-03 | 0.004 | 5337 | phospholipase D1 |
| *IFIT3* | -2.182 | 3.216E-03 | 0.004 | 3437 | interferon induced protein with tetratricopeptide repeats 3 |
| *ID2-AS1* | -2.134 | 6.584E-03 | 0.004 | 100506299 | ID2 antisense RNA 1 (head to head) |
| *TGFA* | -2.072 | 2.313E-03 | 0.004 | 7039 | transforming growth factor alpha |
| *TPGS1* | -2.018 | 8.490E-03 | 0.004 | 91978 | tubulin polyglutamylase complex subunit 1 |
| *EPS8L2* | -2.018 | 2.877E-03 | 0.004 | 64787 | EPS8 like 2 |
| *PRSS35* | -3.395 | 1.208E-02 | 0.005 | 167681 | protease. serine 35 |
| *HOGA1* | -2.230 | 8.246E-03 | 0.004 | 112817 | 4-hydroxy-2-oxoglutarate aldolase 1 |
| *FAM167B* | -2.844 | 1.065E-02 | 0.005 | 84734 | family with sequence similarity 167 member B |
| *LOC728743* | -2.068 | 6.874E-03 | 0.004 | 728743 | zinc finger protein pseudogene |
| *PCDHB9* | -2.257 | 4.480E-03 | 0.004 | 56127 | protocadherin beta 9 |
| *ZNF554* | -2.050 | 8.127E-03 | 0.004 | 115196 | zinc finger protein 554 |
| *GPER1* | -2.131 | 4.174E-03 | 0.004 | 2852 | G protein-coupled estrogen receptor 1 |
| *CSF3* | -4.102 | 1.372E-02 | 0.005 | 1440 | colony stimulating factor 3 |
| *CNTNAP3* | -2.180 | 8.252E-03 | 0.004 | 79937 | contactin associated protein-like 3 |
| *PCBP3* | -2.801 | 1.085E-02 | 0.005 | 54039 | poly(rC) binding protein 3 |
| *SLFN5* | -2.086 | 2.913E-03 | 0.004 | 162394 | schlafen family member 5 |
| *PCDHGA6* | -2.294 | 7.222E-03 | 0.004 | 56109 | protocadherin gamma subfamily A. 6 |
| *ZNF836* | -2.038 | 7.985E-03 | 0.004 | 162962 | zinc finger protein 836 |
| *FGFBP1* | -13.877 | 1.411E-02 | 0.005 | 9982 | fibroblast growth factor binding protein 1 |
| *OASL* | -3.117 | 1.196E-02 | 0.005 | 8638 | 2'-5'-oligoadenylate synthetase like |
| *SLC5A3* | -2.015 | 6.400E-03 | 0.004 | 6526 | solute carrier family 5 member 3 |
| *TP53INP1* | -2.352 | 3.864E-03 | 0.004 | 94241 | tumor protein p53 inducible nuclear protein 1 |
| *PODNL1* | -2.334 | 3.652E-03 | 0.004 | 79883 | podocan like 1 |
| *FRY* | -2.784 | 1.204E-02 | 0.005 | 10129 | FRY microtubule binding protein |
| *FHL1* | -2.190 | 2.437E-03 | 0.004 | 2273 | four and a half LIM domains 1 |
| *CTHRC1* | -2.111 | 6.703E-03 | 0.004 | 115908 | collagen triple helix repeat containing 1 |
| *MGST2* | -2.246 | 4.770E-03 | 0.004 | 4258 | microsomal glutathione S-transferase 2 |
| *ZDHHC22* | -2.357 | 1.012E-02 | 0.005 | 283576 | zinc finger DHHC-type containing 22 |
| *CHST1* | -2.220 | 5.219E-03 | 0.004 | 8534 | carbohydrate sulfotransferase 1 |
| *FBXO27* | -2.758 | 1.248E-02 | 0.005 | 126433 | F-box protein 27 |
| *ROGDI* | -2.047 | 3.393E-03 | 0.004 | 79641 | rogdi homolog |
| *CPZ* | -2.100 | 5.824E-03 | 0.004 | 8532 | carboxypeptidase Z |
| *MANSC1* | -2.998 | 1.182E-02 | 0.005 | 54682 | MANSC domain containing 1 |
| *CCT6B* | -2.351 | 1.028E-02 | 0.005 | 10693 | chaperonin containing TCP1 subunit 6B |
| *FGD5* | -2.423 | 1.153E-02 | 0.005 | 152273 | FYVE. RhoGEF and PH domain containing 5 |
| *KIAA1161* | -2.140 | 2.396E-03 | 0.004 | 57462 | KIAA1161 |
| *MILR1* | -2.840 | 1.253E-02 | 0.005 | 284021 | mast cell immunoglobulin like receptor 1 |
| *SAMD11* | -2.094 | 7.181E-03 | 0.004 | 148398 | sterile alpha motif domain containing 11 |
| *PDGFRA* | -2.001 | 4.670E-03 | 0.004 | 5156 | platelet derived growth factor receptor alpha |
| *LOC101927100* | -2.633 | 1.281E-02 | 0.005 | 101927100 | uncharacterized LOC101927100 |
| *CPM* | -2.165 | 3.682E-03 | 0.004 | 1368 | carboxypeptidase M |
| *NR5A2* | -2.645 | 1.268E-02 | 0.005 | 2494 | nuclear receptor subfamily 5 group A member 2 |
| *PLCXD3* | -2.422 | 1.076E-02 | 0.005 | 345557 | phosphatidylinositol specific phospholipase C X domain containing 3 |
| *ZCWPW2* | -3.324 | 1.295E-02 | 0.005 | 152098 | zinc finger CW-type and PWWP domain containing 2 |
| *LOC101927755* | -2.385 | 1.138E-02 | 0.005 | 101927755 | uncharacterized LOC101927755 |
| *ANG* | -2.257 | 1.030E-02 | 0.005 | 283 | angiogenin |
| *ZMYND10* | -2.029 | 5.331E-03 | 0.004 | 51364 | zinc finger MYND-type containing 10 |
| *ADAMTS15* | -2.136 | 3.988E-03 | 0.004 | 170689 | ADAM metallopeptidase with thrombospondin type 1 motif 15 |
| *VAMP5* | -2.248 | 6.727E-03 | 0.004 | 10791 | vesicle associated membrane protein 5 |
| *DCDC1* | -4.789 | 1.433E-02 | 0.006 | 341019 | doublecortin domain containing 1 |
| *SHC2* | -2.277 | 1.014E-02 | 0.005 | 25759 | SHC adaptor protein 2 |
| *SLC45A1* | -2.605 | 1.280E-02 | 0.005 | 50651 | solute carrier family 45 member 1 |
| *LRRIQ1* | -2.114 | 9.480E-03 | 0.004 | 84125 | leucine rich repeats and IQ motif containing 1 |
| *LPAR6* | -3.008 | 1.327E-02 | 0.005 | 10161 | lysophosphatidic acid receptor 6 |
| *PYROXD2* | -2.242 | 1.036E-02 | 0.005 | 84795 | pyridine nucleotide-disulphide oxidoreductase domain 2 |
| *CLDN3* | -2.275 | 1.134E-02 | 0.005 | 1365 | claudin 3 |
| *TSPYL5* | -2.084 | 6.514E-03 | 0.004 | 85453 | TSPY like 5 |
| *JMJD1C-AS1* | -2.767 | 1.199E-02 | 0.005 | 84989 | JMJD1C antisense RNA 1 |
| *FBLN7* | -2.478 | 1.211E-02 | 0.005 | 129804 | fibulin 7 |
| *MEOX1* | -3.097 | 1.328E-02 | 0.005 | 4222 | mesenchyme homeobox 1 |
| *TRAPPC6A* | -2.000 | 8.376E-03 | 0.004 | 79090 | trafficking protein particle complex 6A |
| *IGFBP7* | -2.083 | 4.616E-03 | 0.004 | 3490 | insulin like growth factor binding protein 7 |
| *NPY* | -2.879 | 1.286E-02 | 0.005 | 4852 | neuropeptide Y |
| *ARL14* | -4.308 | 1.586E-02 | 0.006 | 80117 | ADP ribosylation factor like GTPase 14 |
| *FADS2* | -2.025 | 7.944E-03 | 0.004 | 9415 | fatty acid desaturase 2 |
| *CGREF1* | -2.214 | 1.129E-02 | 0.005 | 10669 | cell growth regulator with EF-hand domain 1 |
| *LOC105373383* | -2.469 | 1.166E-02 | 0.005 | 105373383 | uncharacterized LOC105373383 |
| *SMPDL3B* | -2.851 | 1.333E-02 | 0.005 | 27293 | sphingomyelin phosphodiesterase acid like 3B |
| *METTL7A* | -14.143 | 1.601E-02 | 0.006 | 25840 | methyltransferase like 7A |
| *PAPLN* | -2.024 | 5.907E-03 | 0.004 | 89932 | papilin. proteoglycan like sulfated glycoprotein |
| *SQRDL* | -2.030 | 3.717E-03 | 0.004 | 58472 | sulfide quinone reductase-like (yeast) |
| *JUND* | -2.032 | 2.657E-03 | 0.004 | 3727 | JunD proto-oncogene. AP-1 transcription factor subunit |
| *LRRN2* | -2.058 | 2.710E-03 | 0.004 | 10446 | leucine rich repeat neuronal 2 |
| *MIR4653* | -2.565 | 1.359E-02 | 0.005 | 100616117 | microRNA 4653 |
| *TMEM130* | -3.350 | 1.481E-02 | 0.006 | 222865 | transmembrane protein 130 |
| *CAPS* | -2.102 | 1.025E-02 | 0.005 | 828 | calcyphosine |
| *PCDHB11* | -2.308 | 1.228E-02 | 0.005 | 56125 | protocadherin beta 11 |
| *CDC42EP5* | -2.808 | 1.395E-02 | 0.005 | 148170 | CDC42 effector protein 5 |
| *RNASEL* | -2.341 | 1.273E-02 | 0.005 | 6041 | ribonuclease L |
| *IFITM1* | -2.238 | 1.234E-02 | 0.005 | 8519 | interferon induced transmembrane protein 1 |
| *FAM26E* | -2.885 | 1.492E-02 | 0.006 | 254228 | family with sequence similarity 26 member E |
| *LINC00663* | -2.035 | 1.037E-02 | 0.005 | 284440 | long intergenic non-protein coding RNA 663 |
| *HRH2* | -2.137 | 1.123E-02 | 0.005 | 3274 | histamine receptor H2 |
| *SCN1B* | -2.018 | 3.617E-03 | 0.004 | 6324 | sodium voltage-gated channel beta subunit 1 |
| *CARD6* | -2.260 | 1.237E-02 | 0.005 | 84674 | caspase recruitment domain family member 6 |
| *GATS* | -2.025 | 1.029E-02 | 0.005 | 352954 | GATS. stromal antigen 3 opposite strand |
| *ADAMTS7* | -2.011 | 1.027E-02 | 0.005 | 11173 | ADAM metallopeptidase with thrombospondin type 1 motif 7 |
| *DKKL1* | -2.478 | 1.387E-02 | 0.005 | 27120 | dickkopf like acrosomal protein 1 |
| *LRP1* | -2.021 | 1.070E-02 | 0.005 | 4035 | LDL receptor related protein 1 |
| *ELFN1* | -2.030 | 1.051E-02 | 0.005 | 392617 | extracellular leucine rich repeat and fibronectin type III domain containing 1 |
| *MEGF6* | -2.254 | 1.351E-02 | 0.005 | 1953 | multiple EGF like domains 6 |
| *HVCN1* | -2.071 | 1.147E-02 | 0.005 | 84329 | hydrogen voltage gated channel 1 |
| *MLC1* | -5.577 | 1.639E-02 | 0.006 | 23209 | megalencephalic leukoencephalopathy with subcortical cysts 1 |
| *PHYHIP* | -2.434 | 1.434E-02 | 0.006 | 9796 | phytanoyl-CoA 2-hydroxylase interacting protein |
| *LRRTM2* | -2.281 | 1.318E-02 | 0.005 | 26045 | leucine rich repeat transmembrane neuronal 2 |
| *HES2* | -2.277 | 1.299E-02 | 0.005 | 54626 | hes family bHLH transcription factor 2 |
| *TMEM37* | -3.798 | 1.671E-02 | 0.006 | 140738 | transmembrane protein 37 |
| *ALOX12B* | -2.844 | 1.498E-02 | 0.006 | 242 | arachidonate 12-lipoxygenase. 12R type |
| *NMRK1* | -2.609 | 1.514E-02 | 0.006 | 54981 | nicotinamide riboside kinase 1 |
| *MYOZ1* | -3.014 | 1.701E-02 | 0.006 | 58529 | myozenin 1 |
| *HTR6* | -2.471 | 1.468E-02 | 0.006 | 3362 | 5-hydroxytryptamine receptor 6 |
| *LRRC26* | -2.227 | 1.410E-02 | 0.005 | 389816 | leucine rich repeat containing 26 |
| *FLJ46906* | -2.552 | 1.489E-02 | 0.006 | 441172 | uncharacterized LOC441172 |
| *TMEM92* | -2.091 | 1.367E-02 | 0.005 | 162461 | transmembrane protein 92 |
| *GBP1* | -2.314 | 1.532E-02 | 0.006 | 2633 | guanylate binding protein 1 |
| *PLXNB3* | -2.175 | 1.386E-02 | 0.005 | 5365 | plexin B3 |
| *RTCA-AS1* | -3.550 | 1.858E-02 | 0.006 | 100506007 | RTCA antisense RNA 1 |
| *LOC100505942* | -2.768 | 1.718E-02 | 0.006 | 100505942 | uncharacterized LOC100505942 |
| *C6orf165* | -2.070 | 1.363E-02 | 0.005 | NA | NA |
| *CXCL2* | -2.333 | 1.550E-02 | 0.006 | 2920 | C-X-C motif chemokine ligand 2 |
| *PPP1R3G* | -2.474 | 1.798E-02 | 0.006 | 648791 | protein phosphatase 1 regulatory subunit 3G |
| *TNFSF15* | -2.803 | 1.711E-02 | 0.006 | 9966 | tumor necrosis factor superfamily member 15 |
| *ESRP1* | -3.003 | 1.823E-02 | 0.006 | 54845 | epithelial splicing regulatory protein 1 |
| *MR1* | -2.128 | 1.445E-02 | 0.006 | 3140 | major histocompatibility complex. class I-related |
| *MGC16275* | -3.251 | 1.947E-02 | 0.007 | 85001 | uncharacterized protein MGC16275 |
| *TLE6* | -2.394 | 1.793E-02 | 0.006 | 79816 | transducin like enhancer of split 6 |
| *ADAMTS14* | -2.935 | 1.882E-02 | 0.007 | 140766 | ADAM metallopeptidase with thrombospondin type 1 motif 14 |
| *MIR210HG* | -2.500 | 1.756E-02 | 0.006 | 100506211 | MIR210 host gene |
| *DGKG* | -3.458 | 2.020E-02 | 0.007 | 1608 | diacylglycerol kinase gamma |
| *KCNJ10* | -2.232 | 1.557E-02 | 0.006 | 3766 | potassium voltage-gated channel subfamily J member 10 |
| *PRSS16* | -2.168 | 1.481E-02 | 0.006 | 10279 | protease. serine 16 |
| *CMPK2* | -2.082 | 1.479E-02 | 0.006 | 129607 | cytidine/uridine monophosphate kinase 2 |
| *CASP1* | -2.132 | 1.484E-02 | 0.006 | 834 | caspase 1 |
| *MYO15B* | -3.190 | 2.026E-02 | 0.007 | 80022 | myosin XVB |
| *NEURL3* | -3.101 | 1.969E-02 | 0.007 | 93082 | neuralized E3 ubiquitin protein ligase 3 |
| *PAXIP1-AS2* | -2.163 | 1.516E-02 | 0.006 | 100132707 | PAXIP1 antisense RNA 2 |
| *FGF8* | -3.305 | 1.957E-02 | 0.007 | 2253 | fibroblast growth factor 8 |
| *DHX58* | -2.617 | 1.860E-02 | 0.006 | 79132 | DEXH-box helicase 58 |
| *LKAAEAR1* | -5.744 | 2.187E-02 | 0.007 | 198437 | LKAAEAR motif containing 1 |
| *CD14* | -2.473 | 1.654E-02 | 0.006 | 929 | CD14 molecule |
| *CLDN23* | -2.161 | 1.531E-02 | 0.006 | 137075 | claudin 23 |
| *SNAI3* | -2.402 | 1.673E-02 | 0.006 | 333929 | snail family transcriptional repressor 3 |
| *KIAA1107* | -2.014 | 1.427E-02 | 0.006 | 23285 | KIAA1107 |
| *PTGS2* | -2.436 | 1.852E-02 | 0.006 | 5743 | prostaglandin-endoperoxide synthase 2 |
| *RARB* | -2.439 | 1.866E-02 | 0.006 | 5915 | retinoic acid receptor beta |
| *MX1* | -2.218 | 1.760E-02 | 0.006 | 4599 | MX dynamin like GTPase 1 |
| *KCNQ1* | -2.024 | 1.554E-02 | 0.006 | 3784 | potassium voltage-gated channel subfamily Q member 1 |
| *LOC100130238* | -2.544 | 2.011E-02 | 0.007 | 100130238 | uncharacterized LOC100130238 |
| *PPFIA4* | -2.071 | 1.749E-02 | 0.006 | 8497 | PTPRF interacting protein alpha 4 |
| *HLA-DQB1* | -2.872 | 2.060E-02 | 0.007 | 3119 | major histocompatibility complex. class II. DQ beta 1 |
| *LCA5L* | -2.204 | 1.628E-02 | 0.006 | 150082 | LCA5L. lebercilin like |
| *TMEM255A* | -2.956 | 2.036E-02 | 0.007 | 55026 | transmembrane protein 255A |
| *CCDC170* | -2.187 | 1.663E-02 | 0.006 | 80129 | coiled-coil domain containing 170 |
| *RAD9B* | -2.095 | 1.762E-02 | 0.006 | 144715 | RAD9 checkpoint clamp component B |
| *C15orf65* | -2.302 | 1.843E-02 | 0.006 | 145788 | chromosome 15 open reading frame 65 |
| *PGAP3* | -2.159 | 1.696E-02 | 0.006 | 93210 | post-GPI attachment to proteins 3 |
| *FAM19A3* | -2.535 | 1.954E-02 | 0.007 | 284467 | family with sequence similarity 19 member A3. C-C motif chemokine like |
| *LOC100996455* | -2.572 | 1.983E-02 | 0.007 | 100996455 | uncharacterized LOC100996455 |
| *B3GNT7* | -2.364 | 1.915E-02 | 0.007 | 93010 | UDP-GlcNAc:betaGal beta-1.3-N-acetylglucosaminyltransferase 7 |
| *LINC01023* | -3.295 | 2.199E-02 | 0.007 | 100652853 | long intergenic non-protein coding RNA 1023 |
| *PPP1R16B* | -2.138 | 1.787E-02 | 0.006 | 26051 | protein phosphatase 1 regulatory subunit 16B |
| *GPR146* | -2.198 | 1.869E-02 | 0.006 | 115330 | G protein-coupled receptor 146 |
| *LINC00649* | -2.125 | 1.782E-02 | 0.006 | 100506334 | long intergenic non-protein coding RNA 649 |
| *IDNK* | -2.038 | 1.698E-02 | 0.006 | 414328 | IDNK. gluconokinase |
| *AZU1* | -2.231 | 1.923E-02 | 0.007 | 566 | azurocidin 1 |
| *ADGRG2* | -2.044 | 1.774E-02 | 0.006 | 10149 | adhesion G protein-coupled receptor G2 |
| *MX2* | -2.121 | 1.825E-02 | 0.006 | 4600 | MX dynamin like GTPase 2 |
| *WAS* | -3.213 | 2.345E-02 | 0.008 | 7454 | Wiskott-Aldrich syndrome |
| *NAP1L3* | -2.157 | 1.907E-02 | 0.007 | 4675 | nucleosome assembly protein 1 like 3 |
| *MCF2L-AS1* | -2.073 | 1.832E-02 | 0.006 | 100289410 | MCF2L antisense RNA 1 |
| *NTF4* | -2.894 | 2.324E-02 | 0.007 | 4909 | neurotrophin 4 |
| *LOC101929427* | -2.154 | 2.025E-02 | 0.007 | 101929427 | uncharacterized LOC101929427 |
| *PKIB* | -3.497 | 2.483E-02 | 0.008 | 5570 | protein kinase (cAMP-dependent. catalytic) inhibitor beta |
| *DIRAS3* | -2.058 | 1.901E-02 | 0.007 | 9077 | DIRAS family GTPase 3 |
| *SH2D3A* | -3.100 | 2.424E-02 | 0.008 | 10045 | SH2 domain containing 3A |
| *HOXA-AS2* | -2.093 | 1.928E-02 | 0.007 | 285943 | HOXA cluster antisense RNA 2 |
| *RGMA* | -2.053 | 1.873E-02 | 0.007 | 56963 | repulsive guidance molecule family member a |
| *ANXA9* | -2.244 | 2.249E-02 | 0.007 | 8416 | annexin A9 |
| *PDGFRL* | -2.078 | 1.973E-02 | 0.007 | 5157 | platelet derived growth factor receptor like |
| *HIST2H2AC* | -2.423 | 2.276E-02 | 0.007 | 8338 | histone cluster 2 H2A family member c |
| *PLSCR4* | -2.389 | 2.293E-02 | 0.007 | 57088 | phospholipid scramblase 4 |
| *NSG1* | -2.029 | 1.965E-02 | 0.007 | 27065 | neuron specific gene family member 1 |
| *TSPAN1* | -2.388 | 2.288E-02 | 0.007 | 10103 | tetraspanin 1 |
| *TPD52L1* | -3.355 | 2.657E-02 | 0.009 | 7164 | tumor protein D52-like 1 |
| *GRAP* | -2.215 | 2.053E-02 | 0.007 | 10750 | GRB2-related adaptor protein |
| *APOL1* | -2.679 | 2.460E-02 | 0.008 | 8542 | apolipoprotein L1 |
| *LMNTD2* | -2.040 | 2.235E-02 | 0.007 | 256329 | lamin tail domain containing 2 |
| *HHIPL2* | -3.805 | 2.665E-02 | 0.009 | 79802 | HHIP like 2 |
| *PPL* | -2.619 | 2.462E-02 | 0.008 | 5493 | periplakin |
| *TTLL6* | -2.404 | 2.366E-02 | 0.008 | 284076 | tubulin tyrosine ligase like 6 |
| *LMOD1* | -2.191 | 2.126E-02 | 0.007 | 25802 | leiomodin 1 |
| *EXOC3L2* | -2.637 | 2.536E-02 | 0.008 | 90332 | exocyst complex component 3 like 2 |
| *FBXO15* | -2.747 | 2.561E-02 | 0.009 | 201456 | F-box protein 15 |
| *KLHL35* | -2.372 | 2.405E-02 | 0.008 | 283212 | kelch like family member 35 |
| *FAM198B* | -2.896 | 2.587E-02 | 0.009 | 51313 | family with sequence similarity 198 member B |
| *ADGRF4* | -2.312 | 2.365E-02 | 0.008 | 221393 | adhesion G protein-coupled receptor F4 |
| *ZP4* | -2.147 | 2.140E-02 | 0.007 | 57829 | zona pellucida glycoprotein 4 |
| *TCP11L2* | -2.527 | 2.553E-02 | 0.008 | 255394 | t-complex 11 like 2 |
| *CD200* | -2.295 | 2.388E-02 | 0.008 | 4345 | CD200 molecule |
| *ECEL1* | -3.085 | 2.754E-02 | 0.009 | 9427 | endothelin converting enzyme like 1 |
| *C18orf32* | -2.514 | 2.572E-02 | 0.009 | 497661 | chromosome 18 open reading frame 32 |
| *FBXL13* | -2.028 | 2.109E-02 | 0.007 | 222235 | F-box and leucine rich repeat protein 13 |
| *EPHA5-AS1* | -2.201 | 2.437E-02 | 0.008 | 100144602 | EPHA5 antisense RNA 1 |
| *LMO2* | -3.064 | 2.800E-02 | 0.010 | 4005 | LIM domain only 2 |
| *LOC100129518* | -2.636 | 2.692E-02 | 0.009 | 100129518 | uncharacterized LOC100129518 |
| *ADRA2B* | -2.073 | 2.409E-02 | 0.008 | 151 | adrenoceptor alpha 2B |
| *TEX21P* | -2.712 | 2.796E-02 | 0.010 | 441687 | testis expressed 21. pseudogene |
| *PCDHB12* | -2.148 | 2.540E-02 | 0.008 | 56124 | protocadherin beta 12 |
| *DOCK9-AS2* | -2.463 | 2.810E-02 | 0.010 | 100861541 | DOCK9 antisense RNA 2 (head to head) |
| *ATP1A1-AS1* | -2.122 | 2.502E-02 | 0.008 | 84852 | ATP1A1 antisense RNA 1 |
| *PLBD1* | -2.300 | 2.662E-02 | 0.009 | 79887 | phospholipase B domain containing 1 |
| *NES* | -2.364 | 2.738E-02 | 0.009 | 10763 | nestin |
| *CMTM1* | -2.266 | 2.686E-02 | 0.009 | 113540 | CKLF like MARVEL transmembrane domain containing 1 |
| *ARHGEF37* | -2.340 | 2.776E-02 | 0.009 | 389337 | Rho guanine nucleotide exchange factor 37 |
| *OR7E14P* | -2.301 | 2.807E-02 | 0.010 | 10819 | olfactory receptor family 7 subfamily E member 14 pseudogene |
| *LOH12CR2* | -2.027 | 2.655E-02 | 0.009 | 503693 | loss of heterozygosity. 12. chromosomal region 2 (non-protein coding) |

^a^Expression ratio (fold change) between compared sample groups. Comparison between *ROBO1-*silenced cells and negative-control cells.

^b^*t*-test *p* value for comparison between sample groups (*ROBO1-*silenced and negative-control cells).

^c^FDR-adjusted *p* value.
