# Supplementary material for: Risk of spontaneous preterm birth and fetal growth associates with fetal SLIT2
Source: PLoS Genet. 2019 Jun 13;15(6):e1008107. doi: 10.1371/journal.pgen.1008107 (PMC6563950; doi:10.1371/journal.pgen.1008107)
Supplement: S14 Table — (DOCX) [file pgen.1008107.s018.docx]

| KEGG ID^a^ | Term | Total amount of  annotated  genes | The amount of significant genes | P.Value^b^ |
| --- | --- | --- | --- | --- |
| 5323 | Rheumatoid arthritis | 60 | 2 | 9,13E-03 |
| 5146 | Amoebiasis | 80 | 2 | 1,59E-02 |
| 920 | Sulfur metabolism | 9 | 1 | 2,22E-02 |
| 4060 | Cytokine-cytokine receptor interaction | 119 | 2 | 3,35E-02 |
| 5150 | Staphylococcus aureus infection | 20 | 1 | 4,87E-02 |
| 532 | Glycosaminoglycan biosynthesis - chondroitin sulfate / dermatan sulfate | 20 | 1 | 4,87E-02 |

^a^Functional analysis of differentially expressed genes was conducted against KEGG database.

^b^Pathways with *p* < 0.05 are shown.
