# Supplementary material for: Risk of spontaneous preterm birth and fetal growth associates with fetal SLIT2
Source: PLoS Genet. 2019 Jun 13;15(6):e1008107. doi: 10.1371/journal.pgen.1008107 (PMC6563950; doi:10.1371/journal.pgen.1008107)
Supplement: S15 Table — (DOCX) [file pgen.1008107.s019.docx]

| KEGG ID^a^ | Term | Total amount of  annotated  genes | The amount of significant genes | P.Value^b^ |
| --- | --- | --- | --- | --- |
| 4640 | Hematopoietic cell lineage | 42 | 14 | 6,57E-08 |
| 4060 | Cytokine-cytokine receptor interaction | 119 | 22 | 1,48E-06 |
| 5150 | Staphylococcus aureus infection | 20 | 8 | 1,03E-05 |
| 4940 | Type I diabetes mellitus | 21 | 8 | 1,58E-05 |
| 4610 | Complement and coagulation cascades | 29 | 9 | 3,10E-05 |
| 4614 | Renin-angiotensin system | 10 | 5 | 1,49E-04 |
| 5323 | Rheumatoid arthritis | 60 | 12 | 1,81E-04 |
| 5144 | Malaria | 29 | 8 | 2,18E-04 |
| 4080 | Neuroactive ligand-receptor interaction | 95 | 15 | 4,58E-04 |
| 5332 | Graft-versus-host disease | 15 | 5 | 1,38E-03 |
| 4145 | Phagosome | 106 | 15 | 1,48E-03 |
| 5140 | Leishmaniasis | 49 | 9 | 2,20E-03 |
| 360 | Phenylalanine metabolism | 11 | 4 | 3,02E-03 |
| 4974 | Protein digestion and absorption | 42 | 8 | 3,03E-03 |
| 5412 | Arrhythmogenic right ventricular cardiomyopathy (ARVC) | 55 | 9 | 4,96E-03 |
| 4621 | NOD-like receptor signaling pathway | 47 | 8 | 6,23E-03 |
| 5320 | Autoimmune thyroid disease | 14 | 4 | 7,94E-03 |
| 5330 | Allograft rejection | 14 | 4 | 7,94E-03 |
| 5310 | Asthma | 8 | 3 | 9,62E-03 |
| 5410 | Hypertrophic cardiomyopathy (HCM) | 61 | 9 | 9,88E-03 |
| 350 | Tyrosine metabolism | 23 | 5 | 1,04E-02 |
| 5322 | Systemic lupus erythematosus | 74 | 10 | 1,23E-02 |
| 5414 | Dilated cardiomyopathy | 64 | 9 | 1,34E-02 |
| 4062 | Chemokine signaling pathway | 120 | 13 | 2,69E-02 |
| 5219 | Bladder cancer | 39 | 6 | 2,73E-02 |
| 982 | Drug metabolism - cytochrome P450 | 20 | 4 | 2,89E-02 |
| 590 | Arachidonic acid metabolism | 20 | 4 | 2,89E-02 |
| 4670 | Leukocyte transendothelial migration | 85 | 10 | 3,01E-02 |
| 4512 | ECM-receptor interaction | 63 | 8 | 3,36E-02 |
| 4672 | Intestinal immune network for IgA production | 21 | 4 | 3,41E-02 |
| 980 | Metabolism of xenobiotics by cytochrome P450 | 23 | 4 | 4,59E-02 |
| 5160 | Hepatitis C | 104 | 11 | 4,65E-02 |
| 4514 | Cell adhesion molecules (CAMs) | 68 | 8 | 4,97E-02 |

^a^Functional analysis of differentially expressed genes was conducted against KEGG database.

^b^Pathways with *p* < 0.05 are shown.
